# A New Series of Cytotoxic Pyrazoline Derivatives as Potential Anticancer Agents that Induce Cell Cycle Arrest and Apoptosis

**DOI:** 10.3390/molecules22101635

**Published:** 2017-09-29

**Authors:** Hong Wang, Jinhong Zheng, Weijie Xu, Cheng Chen, Duncan Wei, Wenxiu Ni, Ying Pan

**Affiliations:** 1 Department of Chemistry, Shantou University Medical College, Shantou 515041, Guangdong, China; 13592851552@163.com (H.W.); jhzheng@stu.edu.cn (J.Z.); 13536920534@163.com (W.X.); 13411963192@163.com (C.C.); 2 Department of Pharmacy, The First Affiliated Hospital of Shantou University Medical College, Shantou 515041, Guangdong, China; weiduncan2012@163.com

**Keywords:** pyrazoline, antiproliferative, cytotoxicity, cell cycle arrest, apoptosis

## Abstract

A new series of pyrazoline derivatives **1b**–**12b** was designed, synthesized and evaluated for antiproliferative activity against three cancer cell lines (HepG-2, Hela and A549). Additionally, NIH/3T3 cell cytotoxicity were tested and the structure activity relationships (SARs) were also determined. Among these new derivatives, the compounds 3-(4-fluorophenyl)-5-(3,4,5-trimethoxythiophenyl)-4,5-dihydro-1*H*-pyrazole-1-carbothioamide (**1b**) and 3-(4-chlorophenyl)-5-(3,4,5-trimethoxythiphenyl)-4,5-dihydro-1*H*-pyrazole-1-carbothioamide (**2b**) showed the best activity against HepG-2 cells, with IC_50_ values of 6.78 μM and 16.02 μM, respectively. They also displayed potent activity against Hela cells; meanwhile, 3-(4-chlorophenyl)-5-(3-bromo-4-hydroxy-5-methoxythiophenyl)-4,5-dihydro-1*H*-pyrazole-1-carbothioamide (**5b**) and 3-(4-bromo-phenyl)-5-(3-bromo-4-hydroxy-5-methoxythiophenyl)-4,5-dihydro-1*H*-pyrazole-1-carbothioamide (**6b**) were also identified as promising anticancer agents against A549 cells owing to their notable inhibitory effect, compared with cisplatin (IC_50_ = 29.48 μM). Furthermore, it was also found that compounds **1b** and **2b** had low cytotoxicity against NIH/3T3 cells and further mechanistic studies revealed that **1b** arrested HepG-2 cells cycle at the G2/M phase at high concentrations and induced apoptosis in HepG-2 cells. Moreover, **1b** upregulated protein expression level of cleaved caspase-3, cleaved PARP, Bax and p53 and downregulated protein expression level of Bcl-2 in dose-dependent way in HepG-2 cells. Thus, this study indicates that compound **1b** might be a promising antitumor drug candidate.

## 1. Introduction

Cancer is considered to be one of the most serious health problems worldwide and is also one of the leading causes of mortality [1,2,3]. Still, the successful treatment of cancer remains a challenge in the 21st century, and there is a need to search for newer and safer anticancer agents that possess a broader spectrum of cytotoxicity to tumor cells [4]. In the past few years, apart from the utility of surgical operations and irradiation, chemotherapy still remains an important option to treat cancer in clinical settings [5].

A potential solution is to explore innovative natural scaffolds to treat cancer. Medicinal chemists have carried out considerable research on pyrazoline derivatives due to their diverse therapeutic applications, extending from central nervous system activity to antimicrobial applications. The most predominant biological activity is observed for a class of antimicrobial agents [6,7,8]. Furthermore, a considerable amount of research has reported that pyrazole-based heterocycles show promising activity against cancer cell lines, including A549 human lung adenocarcinoma cell lines [9,10,11,12,13].

Recently, 2*H*-pyrazoles have been identified as a novel class of potent antitumor agents. A series of 2*H*-pyrazolyl-1-carbothioamides showed good inhibitory activity against the proliferation of HepG-2 cells, Hela cells and A549 cancer cells. Structure-activity relationship (SAR) studies revealed that the activity of pyrazoline and its derivatives is dependent on the presence of the nitrogen heterocyclic moiety [14], which is a structural subunit that is known to have a variety of pharmacological properties and widespread medical biological activities [15]. On the other hand, most 2*H*-pyrazole derivatives are chiral molecules, providing a greater degree of variability in conformation and substitutions, leading to better biological activity [16].

SAR analysis showed that the 2*H*-pyrazole scaffold is the crucial pharmacophore for achieving good inhibitory activity, and the substitutions at the 3, 4 and 5-positions of the phenyl ring significantly increased inhibitory activity [17]. Various pyrazole and pyrazoline derivatives have been identified as inhibitors of cyclin-dependent kinase [18] heat shock proteins [19], vascular endothelium growth factors [20] and P-glycoprotein [21]. Pyrazoloacridine was identified as a DNA topoisomerase inhibitor via high-throughput screening in clinical research and inhibited malignancy, induced apoptosis in myeloma and leukemia cells and displayed preclinical activity in myeloma and leukemia cells both in vitro and in vivo [22]*.*

Based on the knowledge above, we developed a series of new pyrazoline derivatives that exhibited promising antitumor activity. It was envisioned that the substitutions of these pharmacophores might lead to the development of some novel compounds with effective antitumor activity. Herein, we report the synthesis of pyrazoline derivatives and evaluation of these compounds as potent antiproliferative and apoptosis inducing agents.

## 2. Results and Discussion

### 2.1. Chemistry

**Scheme 1:**
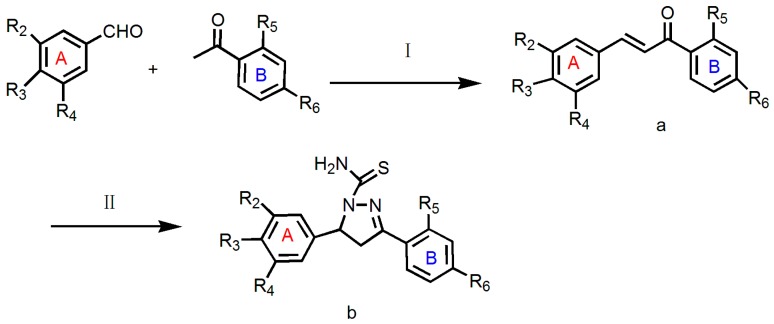
Synthetic route to compounds **1b**–**12b**. *Reagents and Conditions*: (I) absolute ethanol, sodium hydroxide, room temperature 24–48 h, yield 60–80%; (II) absolute ethanol, sodium hydroxide, thiosemicarbazide, reflux 12 h, yield 30–70%.

The general strategies for the synthesis of the target compounds are illustrated in Scheme 1. Intermediate **a** was prepared via a Claisen-Schmidt condensation reaction [23]. NaOH was used as a catalyst in the reaction to obtain 1,3-diaryl prop-2-en-1-ones (chalcones) [24]. Their further condensation with thiosemicarbazides in alcoholic media led to the formation of compounds **1b**–**12b**.

All of the new compounds were characterized on the basis of complementary spectroscopic (^1^H-NMR, ^13^C-NMR, IR and HRMS) and analytical (HPLC) data. The physicochemical properties and spectral data of the synthesized compounds are presented in Table 1.

All of the synthetic compounds gave satisfactory mass spectroscopic data, which were in full accordance with their depicted structures.

### 2.2. Antiproliferative Activity

**Figure 1:**
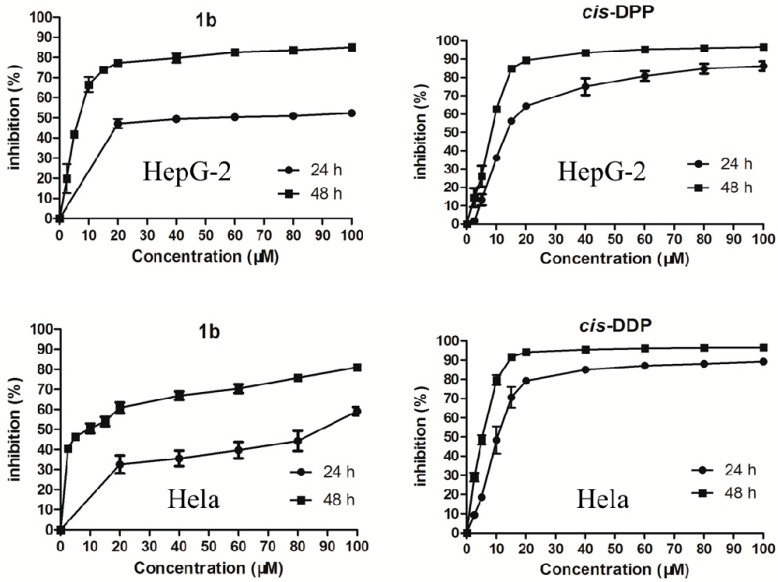
Inhibitory effects of compound **1b** and *cis*-DDP on HepG-2 cells and Hela cells after 24 h and 48 h.

The antiproliferative activity of **1b**–**12b** against three human cancer cell lines (include HepG-2 human hepatoma cell line, Hela human cervical carcinoma cell line and A549 human lung adenocarcinoma cell line) and NIH/3T3 mouse embryonic fibroblast cells were evaluated by the MTT assay (Table 2). Some of them displayed a potent inhibitory activity against HepG-2 cells and Hela cells. The most potent was compound **1b**, with an IC_50_ of 6.78 μM, which is lower than that of *cis*-DPP. However, the compounds were not very sensitive to A549 cells, with IC_50_ values ranging from 40 μM to 100 μM. For Hela cells, the antiproliferative activity of some of the derivatives were similar to that of *cis*-DDP. The pyrazoline derivatives also displayed time-dependent and dose-dependent trends. We selected compound **1b** and *cis*-DDP to generate growth-inhibitory curves (Figure 1) against HepG-2 cells and Hela cells. Most pyrazoline derivatives showed cytotoxic selectivity between tumor and normal mouse embryonic fibroblast cells to some extent, with IC_50_ values ranging from 23.52 μM to 100 μM against NIH/3T3 normal cells. These results confirmed previous reports [25] that some pyrazoline derivatives have less cytotoxicity to normal cells.

In some cases against the selected tumor cell lines, among the derivatives **1b**–**12b**, when the substitutions of R_2_, R_3_ and R_4_ were the same (**1b**, **2b**, **3b**, **4b**, **10b** and **11b**) and R_6_ was changed between F, Cl, Br, OCH_3_ and CH_3_ in each group or R_5_ was replaced with OH, the inhibitory activity of these compounds with different substituents on the B ring increased in the following order: **1b** > **2b** > **3b** > **4b**, **10b** > **11b**. Compounds **5b**, **6b**, **7b** and **12b** with a R_2_ group Br, R_3_ of OH and R_4_ of OCH_3_ showed stronger activity than the corresponding pyrazoline with a R_3_ and R_5_ of OH groups (**8b**). Between alkyl substituents, electron-withdrawing and electron-donating groups at R_6_, the former one (**1b**, **2b**, **3b** and **10b**) was favorable. For example, **1b** has the same substituents at R_2_, R_3_ and R_4_ (OCH_3_) as **2b**, **3b**, **4b**, **10b** and **11b**, but a different R_6_ group. Compound **1b** has an R_6_ of F, a strong electron withdrawing group and showed slightly lower IC_50_ values of 6.78 and 7.63 μM against HepG-2 and Hela tumor cells, respectively. On the other hand, **1b** exhibited low cytotoxicity against A549 cell line and less cytotoxicity to normal cells. A patent has been filed our new compounds [26].

In terms of their anticancer potential, compounds **1b**, **2b** and **3b** can be considered to be selective cytotoxic agents against HepG-2 and Hela cells due to their low cytotoxicity against NIH/3T3 cells.

Among these compounds, compound **9b**, which bears ethyoxyl groups on its phenyl moieties, showed the highest cytotoxicity against Hela cells, with an IC_50_ value of 7.74 μM, which was very similar to the positive control cisplatin (IC_50_ = 5.28 μM). However, compound **7b**, which carries a methoxyl substituent at R_4_ on phenyl ring A, showed low cytotoxicity against HepG-2 cells (IC_50_ > 50 μM) and Hela cells (IC_50_ = 11.44 μM). These results noted the importance of the ethyoxyl group at R_4_ for anticancer activity against HepG-2 and Hela cell lines. It was also found that with a Br group at substituent R_2_, OH at R_3_ and OCH_3_ at R_4_ on phenyl ring A, the selective antiproliferative activities of most compounds were lower. For example, the selective antiproliferative activities of compounds **5b**, **6b**, **7b** and **12b** are all moderate. From these compounds, we also determined that when the substituent R_6_ on the phenyl ring B is an electron-donating group, such as OCH_3_, the antiproliferative activity of the agents can be improved.

Our comparative study demonstrated that compounds **1b** and **2b** can be identified as the most promising anticancer agents owing to their antiproliferative effect on HepG-2 and Hela cancer cells and non-toxic potential against NIH/3T3 cells. Further studies are required to evaluate the mechanism for the anticancer activities of compounds **1b** and **2b**.

**Figure 2:**
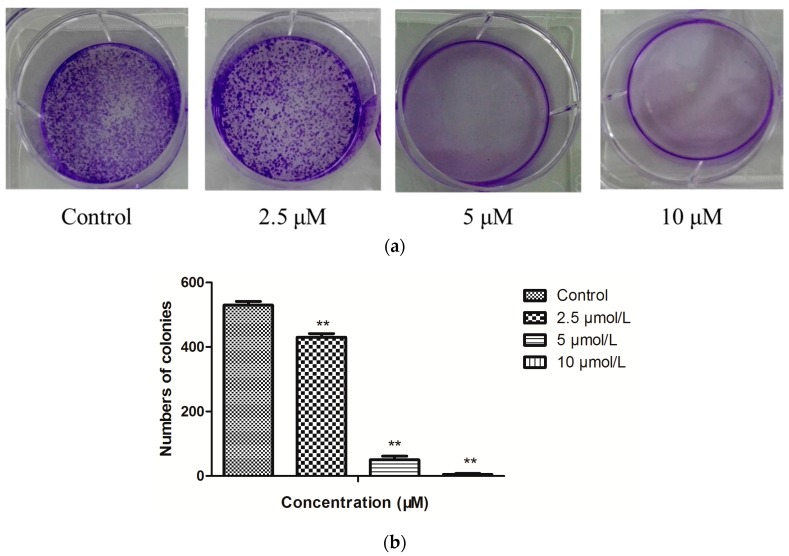
(**a**) Representative images of plate colony formation of HepG-2 cells after treatment with **1b** in different concentrations. (**b**) The quantitative analysis of plate colony formation of HepG-2 cells. *n* = 3; ** *p* < 0.01.

To assess the antiproliferative effect of compound **1b** (Figure 2), we analyzed clonogenicity of HepG-2 cells after the treatment with **1b** for 48 h. As shown in Figure 2, treatment with **1b** significantly reduced cell viability in a dose-dependent fashion in HepG-2 cells.

### 2.3. Influence of **1b** on the HepG-2 Cell Cycle

**Figure 3:**
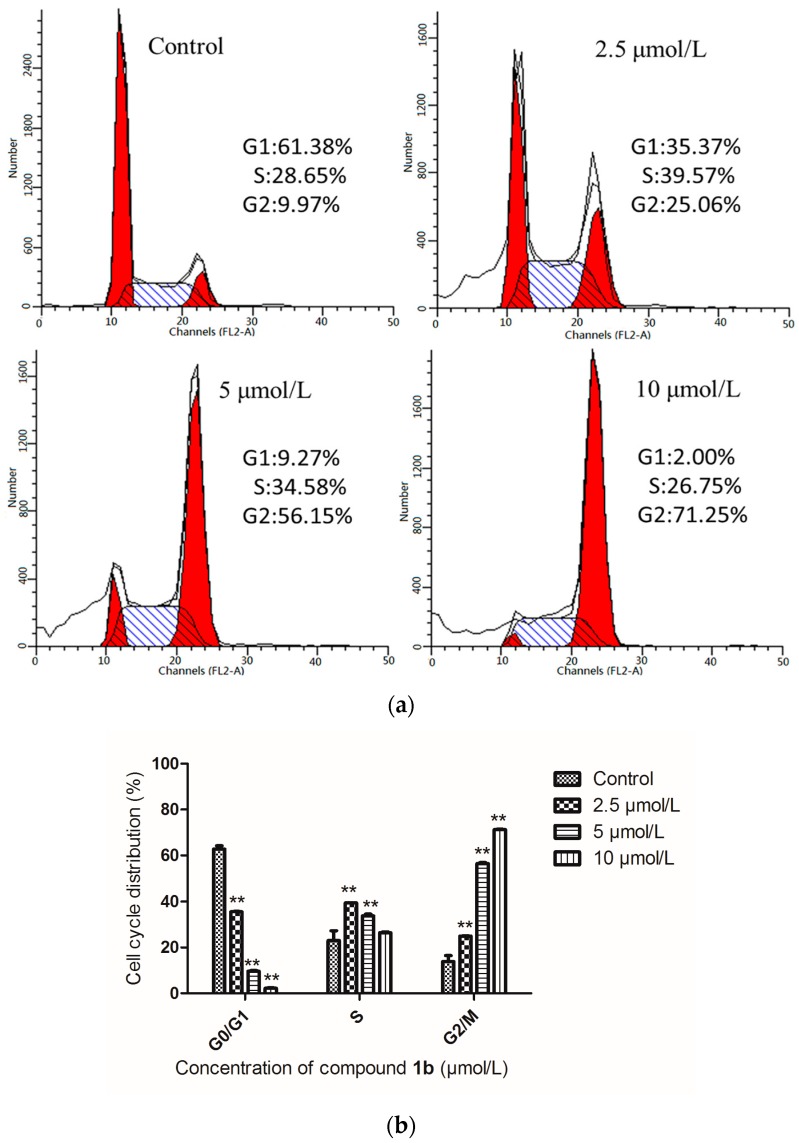
(**a**) Effects of compound **1b** on the cell cycle progression of HepG-2 cells were determined by flow cytometry analysis. HepG-2 cells were treated with different concentrations of compound **1b** for 24 h. The percentage of cells in each cycle phase is indicated (left red for G1, center shadow for S, light red for G2); (**b**) Cell cycle distribution of HepG-2 cells. Compared with the corresponding control group, ** *p* < 0.01, (*n* = 3).

Cell cycle arrest is an important sign for inhibition of proliferation and the series of events that take place in a cell leading to its division and replication. Some pyrazoline derivatives exhibited cell cycle arrest properties [27]. Compound **1b** was chosen for intensive mechanism study on HepG-2 cells because of its highest selectivity index (SI) value of 14.5. After the inhibitory effect of **1b** on cell proliferation was observed, we next assessed the effect on the cell cycle distribution of HepG-2 cells by flow cytometry (Figure 3). Treatment of HepG-2 cells with **1b** at 2.5, 5 and 10 μmol/L concentrations resulted in an increase of the percentage of cells at the G2/M phase to 25.06%, 56.15% and 71.25%, respectively. This represented a remarkable difference from 9.97% in the control group (*p* < 0.05). There was a concomitant decrease of cells in the other phases of the cell cycle (G1 and S), as shown in Figure 3. These findings confirmed that compound **1b** could influence HepG-2 cell cycle progression at low micromolar concentrations in a dose-dependent manner.

### 2.4. Induction of Apoptosis by ***1b***

**Figure 4:**
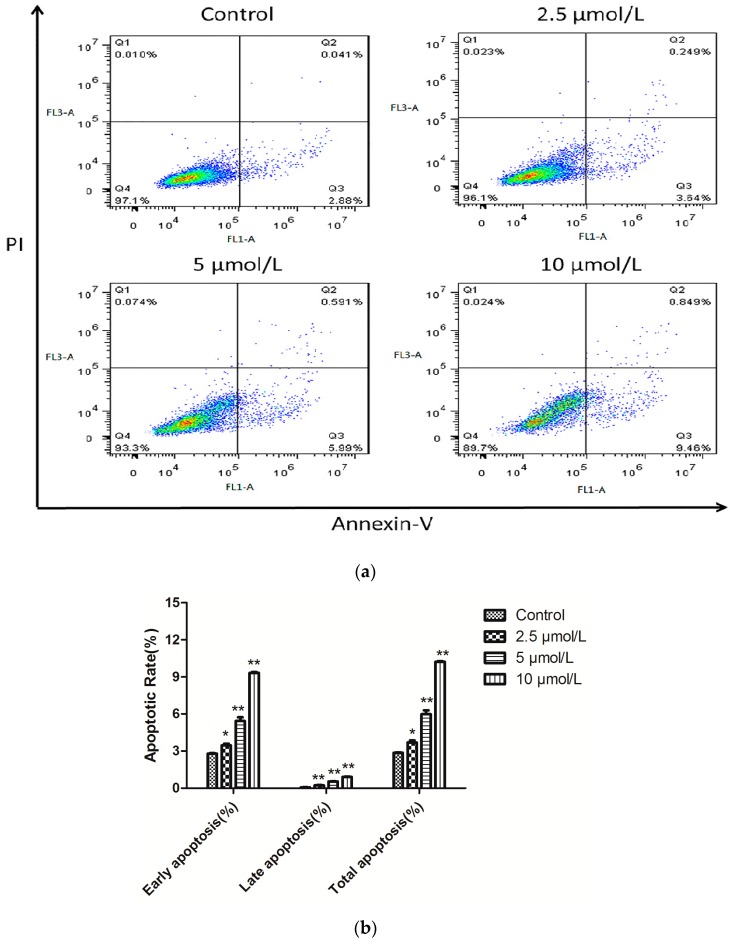
(**a**) Representative flow cytometric histograms of apoptotic HepG-2 cells after a 24 h treatment with compound **1b**. The cells were harvested and labeled with Annexin-V-FITC and PI and then analyzed by flow cytometry; (**b**) Quantitative analysis of the number of apoptotic cells. Compared with the corresponding control group, * *p* < 0.05, ** *p* < 0.01, (*n* = 3).

To examine the potency of the cancer cell apoptosis effect of **1b** on HepG-2 cells, the number of apoptotic cells was monitored using flow cytometry. A biparametric cytofluorimetric analysis was performed using propidium iodide (PI), which stains DNA and only enters dead cells, and fluorescent immunolabeling of the protein annexin-V, which binds to phosphatidyl serine (PS) in a highly selective manner [28]. The total percentage of apoptotic cells (early and late, Q2 + Q3) was 2.921% when treated with vehicle alone. In comparison with the control group, 1.33-, 2.26-, and 3.53-fold percentages of apoptotic cells were observed when different concentrations (2.5, 5 and 10 μmol/L) of **1b** were added to HepG-2 cells. As shown in Figure 4, compound **1b** caused significant induction of apoptosis in a dose-dependent manner in HepG-2 cells and resulted in 3.889%, 6.581% and 10.309% apoptotic cells.

### 2.5. Analysis of Targeted Cellular Pathways

**Figure 5:**
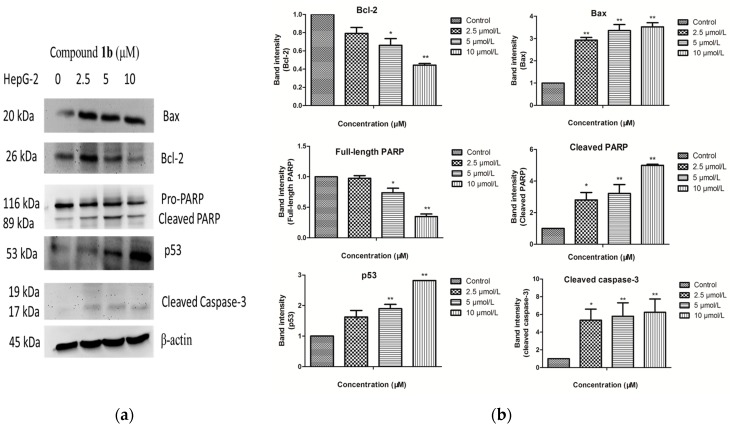
(**a**) HepG-2 cells were treated with compound **1b** at the indicated concentrations (0, 2.5, 5 and 10 μM) for 24 h. The levels of Bcl-2, Bax, PARP, p53 and cleaved caspase-3 were determined by western blot analysis; (**b**) Data are showed as the mean values ± SD from three independent experiments. Data were analyzed by Student’s t-test. * *p* < 0.05, ** *p* < 0.01 vs. control cells.

To explore the mechanisms underlying apoptosis of compound **1b** in HepG-2 cells, the characteristics of apoptosis including p53, caspase 3 and PARP cleavage were examined. As shown in Figure 4, compound **1b** dramatically increased the levels of p53 and cleaved Casp-3, which can cleave essential structural proteins and liberates the DNase to digest chromosomal DNA and cause cell death. Consistently, PARP was cleaved into fragmentation of 89 kDa. The features of apoptosis appeared to occur in a dose-dependent manner. Furthermore, we measured the effect of **1b** on expression of anti-apoptotic protein Bcl-2 and pro-apoptotic protein Bax in HepG-2 cells. Western blot analysis showed an increase in expression of protein Bax, and a decrease in expression of protein Bcl-2 (Figure 5).

## 3. Materials and Methods

### 3.1. General Information

All reagents were commercially available and used without further purification unless otherwise noted. The melting points were determined using a WRS-1B digital melting point apparatus (Weiss-Gallenkamp, Loughborough, UK) by an open capillary method and are reported uncorrected. Thin-layer chromatography (TLC) was performed on silica gel F_254_ plates with visualization by UV light or iodine vapor. The ^1^H-NMR spectra of DMSO-*d*_6_ solutions (tetramethylsilane as an internal standard) were recorded on an AVANCE Ⅱ-400 (400 MHz) spectrometer (Bruker, Karlsruhe, Germany). The ^13^C-NMR spectra were measured at 150 MHz on a Bruker AVANCE III spectrometer. The purity of all compounds were confirmed to be higher than 95% through analytical HPLC performed with a 1200 HPLC System (Agilent, Palo alto, CA, USA) (Supporting Information, Table S5). The IR spectra were recorded on an iS5 FT-IR spectrophotometer (Nicolet, Thermo, Waltham, MA, USA) as KBr pellets or thin films. Mass spectra were obtained with an API 4000 Spectrometer (SCIEX, Los Angeles, CA, USA). High resolution mass spectra (HRMS) were obtained on a Q Exactive^TM^ (Thermo Scientific^TM^, Waltham, MA, USA). Chalcones were prepared from substituted aldehydes and substituted acetophenones according to the procedure reported in the literature [24].

### 3.2. Chemistry: General Procedure for the Synthesis of Chalcones

A mixture of substituted aldehyde (0.05 mol), substituted acetophenone (0.05 mol) and 10% aqueous sodium hydroxide (10 mL) in ethanol (30 mL) was stirred at room temperature for 24–48 h. The progress of the reaction was monitored by TLC (petroleum ether: ethyl acetate (3:1 *v*/*v*) as eluents). Upon completion, the reaction mixture was poured onto crushed ice. The precipitated solid was filtered, washed with water and dried. The products were crystallized from ethanol.

### 3.3. General Procedure for the Synthesis of 3,5-Disubstituted-4,5-dihydro-1H-pyrazole-1-carbothioamides ***1b***–***12b***

A mixture of thiosemicarbazide (0.01 mol), chalcone (0.01 mol) and sodium hydroxide (0.025 mol) was added in ethanol (25 mL). After the mixture was refluxed for 12 h, and after completion of the reaction, the solution was poured into ice water. The resulting precipitate was filtered and recrystallized from ethanol.

*3-(4-Fluorophenyl)-5-(34,5-trimethoxythiophenyl)-4,5-dihydro-1H-pyrazole-1-carbothioamide* (**1b**). Light yellow solid (yield 24.91%), m.p.: 154.2–154.5 °C. IR (KBr, ν in cm^−1^): 3389.05 (N-H), 3264.36 (N-H), 3162.87 (ArC-H), 2938.20 (C-H), 1595.34 (C=N), 1508.34 (C=C), 1234.15 (C-O), 1125.87 (C=S). ^1^H-NMR (ppm) δ: 8.08 (s, 1H, NH_2_), 8.01 (s, 1H, NH_2_), 7.95 (dd, 2H, *J* = 4.0 and 4.0 Hz, Ar-H), 7.31 (t, 2H, *J* = 8.0 Hz, Ar-H), 6.42 (s, 2H, Ar-H), 5.87 (dd, 1H, *J* = 4.0 and 12.0 Hz, pyrazoline 5-H), 3.87 (dd, 1H, *J* = 12.0 and 16.0 Hz, pyrazoline 4-H*_cis_*), 3.19 (dd, 1H, *J* = 3.6 and 16.0 Hz, pyrazoline 4-H*_trans_*), 3.71 (s, 6H, OCH_3_), 3.63 (s, 3H, OCH_3_). ^13^C-NMR (ppm) δ: 176.45, 164.23, 162.58, 154.18, 152.92, 138.66, 136.36, 129.56, 129.50, 127.54, 127.52, 115.80, 115.66, 102.52, 63.06, 59.87 (2C), 55.79, 42.48. MS (ESI) (*m*/*z*): 390.1 [M + H]^+^. HRMS: *m*/*z* [M + H]^+^ calcd for C_19_H_21_N_3_O_3_FS: 390.1282; found: 390.1277.

*3-(4-Chlorophenyl)-5-(3,4,5-trimethoxythiophenyl)-4,5-dihydro-1H-pyrazole-1-carbothioamide* (**2b**). Light white solid (yield 29.81%), m.p.: 169.3–172.1 °C. IR (KBr, ν in cm^−1^): 3412.80 (N-H), 3264.79 (N-H), 3145.37 (ArC-H), 2930.39 (C-H), 1588.19 (C=N), 1459.93 (C=C), 1242.58 (C-O), 1126.07 (C=S). ^1^H-NMR (ppm) δ: 8.11 (s, 1H, NH_2_), 8.00 (s, 1H, NH_2_), 7.90 (d, 2H, *J* = 8.0 Hz, Ar-H), 7.53 (d, 2H, *J* = 8.0 Hz, Ar-H), 6.42 (s, 2H, Ar-H), 5.87 (dd, 1H, *J* = 4.0 and 12.0 Hz, pyrazoline 5-H), 3.86 (dd, 1H, *J* = 8.0 and 16.0 Hz, pyrazoline 4-H*_cis_*), 3.19 (dd, 1H, *J* = 4.0 and 16.0 Hz, pyrazoline 4-H*_trans_*), 3.71 (s, 6H, OCH_3_), 3.62 (s, 3H, OCH_3_). ^13^C-NMR (ppm) δ: 176.54, 154.01, 152.92 (2C), 138.61, 136.36, 135.11, 129.84, 128.84 (2C), 128.72 (2C), 102.52 (2C), 63.13, 59.87 (2C), 55.80, 42.31. MS (ESI) (*m*/*z*): 406.1, 408.1 [M + H]^+^. HRMS: *m*/*z* [M + H]^+^ calcd for C_19_H_21_N_3_O_3_ClS: 406.0987; found: 406.0979.

*3-(4-Bromophenyl)-5-(3,4,5-trimethoxythiophenyl)-4,5-dihydro-1H-pyrazole-1-carbothioamide* (**3b**). White solid (yield 24.55%), m.p.: 271.2–273.0 °C. IR (KBr, ν in cm^−1^): 3411.01 (N-H), 3266.14 (N-H), 3145.76 (ArC-H), 1587.83 (C=N), 1462.14 (C=C), 1247.93 (C-O), 1123.65 (C=S). ^1^H-NMR (ppm) δ: 8.11 (s, 1H, NH_2_), 8.00 (s, 1H, NH_2_), 7.83 (d, 2H, *J* = 8.0 Hz, Ar-H), 7.67 (d, 2H, *J* = 8.0 Hz, Ar-H), 6.41 (s, 2H, Ar-H), 5.87 (dd, 1H, *J* = 4.0 and 12.0 Hz, pyrazoline 5-H), 3.86 (dd, 1H, *J* = 8.0 and 16.0 Hz, pyrazoline 4-H*_cis_*), 3.71 (s, 6H, OCH_3_), 3.62 (s, 3H, OCH_3_), 3.19 (dd, 1H, *J* = 4.0 and 16.0 Hz, pyrazoline 4-H*_trans_*). ^13^C-NMR (ppm) δ: 177.06, 154.61, 153.41 (2C), 132.14 (2C), 130.69 (2C), 129.56 (2C), 124.45 (2C), 103.09 (2C), 63.63, 60.39 (2C), 56.34, 42.77. MS (ESI) (*m*/*z*): 450.1, 452.2 [M + H]^+^. HRMS: *m*/*z* [M + H]^+^ calcd for C_19_H_2__0_N_3_O_3_BrS: 450.0487; found: 450.0468.

*3-(4-Methylphenyl)-5-(3,4,5-trimethoxythiophenyl)-4,5-dihydro-1H-pyrazole-1-carbothioamide* (**4b**). Light yellow solid (yield 62.75%), m.p.: 182.5–184.3 °C. IR (KBr, ν in cm^−1^): 3431.26 (N-H), 3264.45 (N-H), 3137.44 (ArC-H), 1584.57 (C=N), 1466.08 (C=C), 1241.98 (C-O), 1127.06 (C=S). ^1^H-NMR (ppm) δ: 8.05 (s, 1H, NH_2_), 7.89 (s, 1H, NH_2_), 7.78 (d, 2H, *J* = 8.0 Hz, Ar-H), 7.21 (d, 2H, *J* = 8.0 Hz, Ar-H), 6.47 (s, 2H, Ar-H), 5.86 (dd, 1H, *J* = 4.0 and 12.0 Hz, pyrazoline 5-H), 3.85 (dd, 1H, *J* = 12.0 and 16.0 Hz, pyrazoline 4-H*_cis_*), 3.18 (dd, 1H, *J* = 4.0 and 20.0 Hz, pyrazoline 4-H*_trans_*), 3.71 (s, 6H, OCH_3_), 3.62 (s, 3H, OCH_3_), 2.35 (s, 3H, CH_3_). ^13^C-NMR (ppm) δ: 176.77, 155.70, 153.40 (2C), 141.00 (2C), 139.21 (2C), 129.74 (2C), 127.66 (2C), 103.05 (2C), 63.34, 60.39 (2C), 56.32, 42.95, 21.53. MS (ESI) (*m*/*z*): 386.0 [M + H]^+^. HRMS: *m*/*z* [M + Na]^+^ calcd for C_20_H_2__3_N_3_O_3_S: 408.1358; found: 408.1338.

*3-(4-Chlorophenyl)-5-(3-bromo-4-hydroxy-5-methoxythiophenyl)-4,5-dihydro-1H-pyrazole-1-carbothioamide* (**5b**). White solid (yield 14.40%), m.p.: 214.0–217.8 °C. IR (KBr, ν in cm^−1^): 3422.81 (O-H), 3405.51 (N-H), 3253.05 (N-H), 3152.44 (ArC-H), 1595.04 (C=N), 1473.05 (C=C), 1235.36 (C-O), 1176.17 (C=S). ^1^H-NMR (ppm) δ: 9.41 (s, 1H, OH), 8.11 (s, 1H, NH_2_), 8.00 (s, 1H, NH_2_), 7.90 (d, 2H, *J* = 8.0 Hz, Ar-H), 7.54 (d, 2H, *J* = 12.0 Hz, Ar-H), 6.79 (s, 1H, Ar-H), 6.71 (s, 1H, Ar-H), 5.84 (dd, 1H, *J* = 4.0 and 12.0 Hz, pyrazoline 5-H), 3.84 (dd, 1H, *J* = 12.0 and 20.0 Hz, pyrazoline 4-H*_cis_*), 3.19 (dd, 1H, *J* = 4.0 and 20.0 Hz, pyrazoline 4-H*_trans_*), 3.78 (s, 3H, OCH_3_). ^13^C-NMR (DMSO-*d*_6_, ppm) δ: 176.89, 154.46, 148.95, 143.20, 135.65, 135.33, 130.34 (2C), 129.36, 129.25, 120.97 (2C), 109.62, 109.48, 62.91, 56.65, 42.71. MS (ESI) (*m*/*z*): 440.3, 441.9 [M + H]^+^. HRMS: *m*/*z* [M + Na]^+^ calcd for C_17_H_15_ClN_3_O_2_S: 461.9634; found: 461.9631.

*3-(4-Bromophenyl)-5-(3-bromo-4-hydroxy-5-methoxythiophenyl)-4,5-dihydro-1H-pyrazole-1-carbothioamide* (**6b**). White solid (yield 22.46%), m.p.: 228.2–228.6 °C. IR (KBr, ν in cm^−1^): 3422.81 (O-H), 3408.02 (N-H), 3252.81 (N-H), 3152.79 (ArC-H), 1592.59 (C=N), 1496.38 (C=C), 1275.39 (C-O), 1173.55 (C=S). ^1^H-NMR (ppm) δ: 9.39 (s, 1H, OH), 8.11 (s, 1H, NH_2_), 7.99 (s, 1H, NH_2_), 7.83 (d, 2H, *J* = 8.0 Hz, Ar-H), 7.67 (d, 2H, *J* = 8.0 Hz, Ar-H), 6.79 (d, 1H, *J* = 4.0 Hz, Ar-H), 6.71 (d, 1H, *J* = 4.0 Hz, Ar-H), 5.83 (dd, 1H, *J* = 4.0 and 12.0 Hz, pyrazoline 5-H), 3.84 (dd, 1H, *J* = 12.0 and 20.0 Hz, pyrazoline 4-H*_cis_*), 3.78 (s, 3H, OCH_3_), 3.19 (dd, 1H, *J* = 4.0 and 20.0 Hz, pyrazoline 4-H*_trans_*). ^13^C-NMR (ppm) δ: 176.79, 155.60, 148.79, 144.20, 138.22, 132.12(2C), 131.24(2C), 130.25, 125.46, 124.19, 113.16, 111.17, 62.62, 56.67, 42.81. MS (ESI) (*m*/*z*): 484.1, 485.9 [M + H]^+^. HRMS: *m*/*z* [M + Na]^+^ calcd for C_17_H_15_Br_2_N_3_O_2_S: 507.9107; found: 507.9097.

*3-(2-Hydroxyphenyl)-5-(3-bromo-4-hydroxy-5-methoxythiophenyl)-4,5-dihydro-1H-pyrazole-1-carbothioamide* (**7b**). White solid (yield 47.24%), m.p.: 263.9–264.5 °C. IR (KBr, ν in cm^−1^): 3414.47 (N-H), 3315.95 (N-H), 3187.99 (ArC-H), 1609.38 (C=N), 1485.98 (C=C), 1249.05 (C-O), 1165.33 (C=S). ^1^H-NMR (ppm) δ: 9.76 (s, 1H, OH), 9.41 (s, 1H, OH), 8.11 (s, 1H, NH_2_), 8.06 (s, 1H, NH_2_), 7.65 (d, 1H, *J* = 8.0 Hz, Ar-H), 7.34 (t, 1H, *J* = 8.0 Hz, Ar-H), 6.93 (m, 2H, Ar-H), 6.81 (s, 1H, Ar-H), 6.72 (s, 1H, Ar-H), 5.83 (dd, 1H, *J* = 4.0 and 12.0 Hz, pyrazoline 5-H), 3.93 (dd, 1H, *J* = 12.0 and 20.0 Hz, pyrazoline 4-H*_cis_*), 3.28 (dd, 1H, *J* = 4.0 and 20.0 Hz, pyrazoline 4-H*_trans_*), 3.79 (s, 3H, OCH_3_). ^13^C-NMR (ppm) δ: 176.46, 157.03, 156.86, 148.96, 143.17, 135.36, 132.58, 130.06, 120.83, 120.00, 117.34, 116.63, 109.62, 109.42, 61.75, 56.64, 44.57. MS (ESI) (*m*/*z*): 422.2, 424.1 [M + H]^+^. HRMS: *m*/*z* [M + Na]^+^ calcd for C_17_H_16_BrN_3_O_3_S: 443.9973; found: 443.9968.

*3-(2-Hydroxyphenyl)-5-(3-methoxy-4-hydroxy-5-methoxythiophenyl)-4,5-dihydro-1H-pyrazole-1-carbothioamide* (**8b**). Yellow solid (yield 21.78%), m.p.: 230.2–230.6 °C. IR (KBr, ν in cm^−1^): 3430.11 (N-H), 3322.13 (N-H), 3178.46 (ArC-H), 2991.86 (C-H), 1609.15 (C=N), 1589.30 (C=C), 1248.53 (C-O), 1118.29 (C=S). ^1^H-NMR (ppm) δ: 9.77 (s, 1H, OH), 8.29 (s, 1H, OH), 8.06 (s, 1H, NH_2_), 8.03 (s, 1H, NH_2_), 7.64 (d, 1H, *J* = 8.0 Hz, Ar-H), 7.33 (t, 1H, *J* = 8.0 Hz, Ar-H), 6.93 (m, 2 H, Ar-H), 6.39 (s, 2H, Ar-H), 5.82 (dd, 1H, *J* = 4.0 and 12.0 Hz, pyrazoline 5-H), 3.91 (dd, 1H, *J* = 12.0 and 20.0 Hz, pyrazoline 4-H*_cis_*), 3.27 (dd, 1H, *J* = 4.0 and 20.0 Hz, pyrazoline 4-H*_trans_*), 3.69 (s, 6H, OCH_3_). ^13^C-NMR (ppm) δ: 176.61, 157.02, 156.97, 148.47, 135.23, 133.49, 132.49, 130.03 (2C), 119.98 (2C), 117.29, 116.73, 103.55, 62.41 (2C), 56.53, 44.69. MS (ESI) (*m*/*z*): 374.1[M + H]^+^. HRMS: *m*/*z* [M + Na]^+^ calcd for C_18_H_19_N_3_O_4_S: 396.0984; found: 396.0972.

*3-(2-Hydroxyphenyl)-5-(3-ethyoxyl-4-hydroxy-5-bromothiophenyl)-4,5-dihydro-1H-pyrazole-1-carbothioamide* (**9b**). White solid (yield 12.49%), m.p.: 240.9–241.1 °C. IR (KBr, ν in cm^−1^): 3425.40 (N-H), 3320.10 (N-H), 3176.22 (ArC-H), 1598.08 (C=N), 1476.57 (C=C), 1251.80 (C-O), 1131.23 (C=S). ^1^H-NMR (ppm) δ: 9.75 (s, 1H, OH), 9.20 (s, 1H, OH), 8.11 (s, 1H, NH_2_), 8.05 (s, 1H, NH_2_), 7.65 (d, 1H, *J* = 4.0 Hz, Ar-H), 7.33 (m, 1H, Ar-H), 6.92 (m, 2H, Ar-H), 6.78 (s, 1H, Ar-H), 6.73 (s, 1H, Ar-H) , 5.81 (dd, 1H, *J* = 4.0 and 12.0 Hz, pyrazoline 5-H), 4.03 (q, 2H, *J* = 8.0Hz, CH_2_), 3.92 (dd, 1H, *J* = 12.0 and 20.0 Hz, pyrazoline 4-H*_cis_*), 3.27 (dd, 1H, *J* = 4.0 and 16.0 Hz, pyrazoline 4-H*_trans_*), 1.33 (t, 3H, *J* = 8.0 Hz, CH_3_). ^13^C-NMR (ppm) δ: 176.44, 157.02, 156.87, 147.96, 143.44, 135.38, 132.57, 130.07, 120.91, 120.00, 117.34, 116.62, 110.67, 109.72, 65.05, 61.72, 44.54, 14.99. MS (ESI) (*m*/*z*): 436.1, 438.0 [M + H]^+^. HRMS: *m*/*z* [M + Na]^+^ calcd for C_18_H_18_BrN_3_O_3_S: 460.0129; found: 460.0108.

*3-(2-Hydroxyphenyl)-5-(3,4,5-trimethoxythiophenyl)-4,5-dihydro-1H-pyrazole-1-carbothioamide* (**10b**). Yellow solid (yield 23.86%), m.p.: 204.8 °C. IR (KBr, ν in cm^−1^): 3436.42 (N-H), 3331.30 (N-H), 3181.19 (ArC-H), 2995.29 (C-H), 2927.28 (C-H), 1594.63 (C=N), 1481.56 (C=C), 1239.70 (C-O), 1121.91 (C=S). ^1^H-NMR (ppm) δ: 9.77 (s, 1H, OH), 8.11 (s, 1H, NH_2_), 8.07 (s, 1H, NH_2_), 7,65 (d, 1H, *J* = 8.0 Hz, Ar-H), 7.33 (t, 1H, *J* = 8.0 Hz), 6.92 (m, 2H, Ar-H), 6.43 (s, 2H, Ar-H), 5.86 (dd, 1H, *J* = 4.0 and 8.0 Hz, pyrazoline 5-H), 3.95 (dd, 1H, *J* = 12.0 and 20.0 Hz, pyrazoline 4-H*_cis_*), 3.27 (dd, 1H, *J* = 4.0 and 16.0 Hz, pyrazoline 4-H*_trans_*), 3.71 (s, 6H, OCH_3_), 3.63 (s, 3H, OCH_3_). ^13^C-NMR (ppm) δ: 176.67, 157.03, 156.93, 153.43, 139.10, 136.91, 132.53, 130.05 (2C), 119.98 (2C), 117.30, 116.69, 103.04, 62.49, 60.40 (2C), 56.33, 44.66. MS (ESI) (*m*/*z*): 388.0 [M + H]^+^. HRMS: *m*/*z* [M + Na]^+^ calcd for C_19_H_2__1_N_3_O_4_S: 410.1140; found: 410.1124.

*3-(2-Methoxyphenyl)-5-(3,4,5-trimethoxythiophenyl)-4,5-dihydro-1H-pyrazole-1-carbothioamide* (**11b**). White solid (yield 44.44%), m.p.: 226.1–228.9 °C. IR (KBr, ν in cm^−1^): 3500.60 (N-H), 3282.19 (N-H), 3113.39 (ArC-H), 2994.50 (C-H), 2934.30 (C-H), 1598.93 (C=N), 1571.10 (C=C), 1241.93 (C-O), 1126.11 (C=S). ^1^H-NMR (ppm) δ: 7.99 (s, 1H, NH_2_), 7.86 (s, 1H, NH_2_), 7.82 (d, 2H, *J* = 8.0 Hz, Ar-H), 7.01 (d, 2H, *J* = 8.0 Hz, Ar-H), 6.42 (s, 2H, Ar-H), 5.85 (dd, 1H, *J* = 4.0 and 12.0 Hz, pyrazoline 5-H), 3.86 (dd, 1H, *J* = 4.0 and 16.0 Hz, pyrazoline 4-H*_cis_*), 3.17 (dd, 1H, *J* = 4.0 and 20.0 Hz, pyrazoline 4-H*_trans_*), 3.81 (s, 3H, OCH_3_), 3.71 (s, 6H, OCH_3_), 3.62 (s, 3H, OCH_3_). ^13^C-NMR (ppm) δ: 176.56, 161.69, 155.54, 153.40, 139.25, 136.87, 129.38 (2C), 123.82 (2C), 114.63 (2C), 103.07 (2C), 63.29, 60.39 (2C), 56.32, 55.87, 43.01. MS(ESI) (*m*/*z*): 402.1 [M + H]^+^. HRMS: *m*/*z* [M + Na]^+^ calcd for C_20_H_2__3_N_3_O_4_S: 424.1301; found: 424.1281.

*3-(4-Methylphenyl)-5-(3-bromo-4-hydroxy-5-methoxythiophenyl)-4,5-dihydro-1H-pyrazole-1-carbothioamide* (**12b**). White solid (yield 23.02%), m.p.: 195.1–196.3 °C. IR (KBr, ν in cm^−1^): 3424.54 (O-H), 3409.33 (N-H), 3252.12 (N-H), 3153.99 (ArC-H), 2934.30 (C-H), 1598.93 (C=N), 1591.93 (C=C), 1275.77 (C-O), 1171.64 (C=S). ^1^H-NMR (ppm) δ: 9.44 (s, 1H, OH), 8.01 (s, 1H, NH_2_), 7.87 (s, 1H, NH_2_), 7.76 (d, 2H, *J* = 8.0 Hz, Ar-H), 7.27 (d, 2H, *J* = 8.0 Hz, Ar-H), 6.79 (s, 1H, Ar-H), 6.69 (s, 1H, Ar-H), 5.82 (dd, 1H, *J* = 4.0 and 12.0 Hz, pyrazoline 5-H), 3.83 (dd, 1H, *J* = 8.0 and 16.0 Hz, pyrazoline 4-H*_cis_*), 3.16 (dd, 1H, *J* = 4.0 and 20.0 Hz, pyrazoline 4-H*_trans_*), 3.77 (s, 1H, OCH_3_), 2.34 (s, 3H, CH_3_). ^13^C-NMR (ppm) δ: 176.59, 155.64, 148.94, 143.13, 141.03, 135.43, 129.76, 128.60, 127.62, 120.90 (2C), 109.61, 109.44, 62.61, 56.64, 42.84, 21.53. MS (ESI) (*m*/*z*): 420.0, 422.0 [M + H]^+^. HRMS: *m*/*z* [M + Na]^+^ calcd for C_18_H_18_BrN_3_O_2_S: 444.0170; found: 444.0150.

### 3.4. Biological Assays

#### 3.4.1. MTT Assay and Clonogenic Survival Assay

A MTT assay was developed to monitor mammalian cell survival and proliferation in vitro [29]. HepG-2 cells, Hela and A549 cells from the China Center for Type Culture Collection (CCTCC, Wuhan, China) were cultivated in RPMI-1640 medium supplemented with 10% fetal bovine serum (*ν*/*ν*), 100 U/mL penicillin, and 50 mg/mL streptomycin. Cells (5 × 10^3^ cell/well) at the log phase of their growth cycle were added to each well of a 96-well plate and incubated for 24 h at 37 °C in a humidified atmosphere of 5% CO_2_. Then, cells were treated with or without test compounds at different concentrations. After 48 h, 5 mg/mL MTT solution (20 μL per well) was added. Cells were incubated at 37 °C. After 4 h, the MTT solution was removed and DMSO was added to each well (150 μL). Eight to twelve minutes later, the optical density (OD) values were measured at room temperature at a wavelength of 595 nm on a FilterMax F3/F5 Microplate Reader (Molecular Devices, Sydney, Australia). In this experiment, 5 mg/mL of cisplatin was used as the positive control and 0.1% DMSO was used as the negative reference. Each assay was carried out at least three times. The results are summarized in Table 2.

Additionally, we used plate colony formation assay to evaluate the colony formation ability of HepG-2 cells. The cells were seeded in the complete medium in 6-well plate at 600 cells per well. After attachment, cells were exposed to different concentrations of compound **1b**. After 7 days, the cells were fixed with paraformaldehyde for 20 min and stained with 1% crystal violet solution for 30 min to observe colonies.

#### 3.4.2. Flow Cytometric Analysis of Cell Cycle Distribution

For flow cytometric analysis of the DNA content, HepG-2 cells (2.0 × 10^5^ cells/well) in exponential growth were plated in 6-well plates and incubated for 24 h and then incubated with different concentrations (2.5, 5 and 10 μmol/L) of compound **1b** at 37 °C for 24 h. After 24 h treatment, cells were collected, centrifuged, and fixed with ice-cold ethanol (70%) overnight 4 °C. The cells were washed twice with PBS and then treated with lysis buffer containing RNase A (Sigma, St. Louis, MO, USA), which was kept in a lukewarm bath at 37 °C, for 30 min, sequentially, and stained with PI and kept in the dark for 30 min [30]. Then, the samples were analyzed by a flow cytometer (FACSCalibur, BD Accuri^TM^ C6 BD Instruments Inc., New York, NY, USA). Each experiment was performed in triplicate. DNA histograms were analyzed using Mod Fit (version 3.2) for Windows (Verity Software House, New York, NY, USA).

#### 3.4.3. Cellular Apoptosis

HepG-2 cells (2.0 × 10^5^ cells/well) were plated in a 6-well plate and allowed to adhere. After 24 h, the medium was replaced with fresh culture medium containing compound **1b** at final concentrations of 2.5, 5, and 10 μmol/L at 37 °C. After 24 h, the cells were harvested and staining solution was added (containing 5 μL Annexin V-FITC and 5 μL PI) [31]. The cells were then incubated for 15 min at 20–25 °C in the dark. The samples were then detected in a FACScalibur BD Accuri^TM^ C6 flow cytometer. Analyses were performed by the software supplied with the instrument.

#### 3.4.4. Western Blot Analysis

HepG-2 cells (1.0 × 10^6^ cells/well) were seeded in six-well plates. After treatment with compound **1b** for 24 h, the preparation of total protein samples from the culture cells for immunoblotting was carried out as previously described [32]. For gel electrophoresis (Novex® NuPAGE® SDS-PAG, Thermo, Waltham, MA, USA), 30–50 μg of protein was used per well. Then the proteins were separated by SDS-PAGE and electroblotted to nitrocellulose membrane. The membrane was incubated with the following indicated antibody: Bcl-2, Bax, caspase-3, p53, PARP and β-actin (Cell Signaling Technology, Beverly, MA, USA) for 12 h at 4 °C. The antibodies were used in 500 to 1000 5% BSA-TBS-T. The blots were washed four times and then incubated with horseradish peroxidase-conjugated secondary antibody (Santa Cruz Biotechnology Laboratories. Santa Cruz, CA, USA) for 1.5 h at room temperature. β-Actin antibody was used for equal loading.

## 4. Conclusions

In summary, a series of pyrazoline derivatives **1b**–**12b** was synthesized. Most of the target compounds showed antiproliferative activity to some extent against HepG-2 cells, Hela cells, A549 cells and NIH/3T3 mouse embryonic fibroblast cells. It was found that **1b** and **2b** with the same substitutions at the R_2_, R_3_ and R_4_ positions were the most active against Hela cells, with IC_50_ values of 7.63 and 9.37 μM, respectively. Compound **1b** displayed the most potent activity against HepG-2 cells, with an IC_50_ of 6.78 μM, which was comparable to that of the positive control cisplatin. Furthermore, we also showed that compound **2b** had good selectivity between tumor and normal cells to a certain extent, with IC_50_ values of 16.02, 9.37, 50 and beyond 100 μM against HepG-2, Hela, A549 and NIH/3T3 cells, respectively. A preliminary SAR study was also concluded based on the obtained biological evaluation data. We observed that compound **1b** was a potent inducer of apoptosis in HepG-2 cells and caused an accumulation of cells in the G2/M phase of the cell cycle. At the same time, the promising compound **1b** decreased the level of Bcl-2 and increased the level of p53, cleaved PARP, Bax and active-caspase-3, which was consistent with the result of flow cytometry analysis in inducing apoptosis. Compounds **5b**, **6b** and **12b** also attracted our attention. This suggested that the scaffold of 2,3,4-trisubstituted phenyl ring A and 2*H*-pyrazoline moiety could be used as a lead structure for further optimization to find more potent antitumor agents.

## Figures and Tables

**Table 1:** Characterization data of compounds **1b**–**12b**.

Compounds	R_2_	R_3_	R_4_	R_5_	R_6_	Mol. Formula
**1b**	OCH_3_	OCH_3_	OCH_3_	H	F	C_19_H_20_FN_3_O_3_S
**2b**	OCH_3_	OCH_3_	OCH_3_	H	Cl	C_19_H_20_ClN_3_O_3_S
**3b**	OCH_3_	OCH_3_	OCH_3_	H	Br	C_19_H_20_BrN_3_O_3_S
**4b**	OCH_3_	OCH_3_	OCH_3_	H	CH_3_	C_20_H_23_N_3_O_3_S
**5b**	Br	OH	OCH_3_	H	Cl	C_17_H_15_BrClN_3_O_2_S
**6b**	Br	OH	OCH_3_	H	Br	C_17_H_15_Br_2_N_3_O_2_S
**7b**	Br	OH	OCH_3_	OH	H	C_17_H_16_BrN_3_O_3_S
**8b**	OCH_3_	OH	OCH_3_	OH	H	C_18_H_19_N_3_O_4_S
**9b**	Br	OH	OCH_2_CH_3_	OH	H	C_16_H_15_N_3_OS
**10b**	OCH_3_	OCH_3_	OCH_2_CH_3_	OH	H	C_19_H_21_N_3_O_4_S
**11b**	OCH_3_	OCH_3_	OCH_3_	H	OCH_3_	C_20_H_23_N_3_O_4_S
**12b**	Br	OH	OCH_3_	H	CH_3_	C_18_H_18_BrN_3_O_2_S

**Table 2:** Antiproliferative activity of the synthesized compounds (IC_50_ μM).

Compounds	(IC_50_ μM) ^1^				
	HepG-2	Hela	A549	NIH/3T3 Cell Line	SI ^3^
**1b**	6.78 ± 1.44	7.63 ± 1.35	>100	>100	>14.75
**2b**	16.02 ± 2.55	9.37 ± 1.47	>50	>100	>6.24
**3b**	35.55 ± 2.56	16.93 ± 1.99	>100	>100	>2.81
**4b**	>50	>50	>100	>100	-
**5b**	33.06 ± 3.54	15.78 ± 2.33	44.12 ± 3.02	26.28 ± 1.81	0.79
**6b**	52.99 ± 2.81	20.20 ± 1.54	50.99 ± 2.11	23.52 ± 1.39	0.44
**7b**	>50	11.44 ± 2.64	>50	>100	-
**8b**	>50	>50	>100	>100	-
**9b**	32.25 ± 2.83	7.74 ± 1.63	>100	>100	>3.10
**10b**	>50	22.31 ± 2.78	>100	>100	-
**11b**	>50	>50	>100	>100	-
**12b**	19.69 ± 2.45	14.82 ± 2.33	50.04 ± 3.10	27.11 ± 2.02	1.38
***cis*-DDP ^2^**	7.57 ± 0.98	5.28 ± 0.66	29.48 ± 3.30	4.88 ± 1.93	0.64

^1^ Each experiment was independently performed three times; ^2^ *cis*-DDP stands for cisplatin (Ⅱ), which was used as a positive control; ^3^ SI: selectivity index. It was calculated as: SI = IC_50_, _NIH/3T3_/IC_50, HepG-2._

## Supplementary Materials

The following are available online.
