# Supplementary material for: A New Series of Cytotoxic Pyrazoline Derivatives as Potential Anticancer Agents that Induce Cell Cycle Arrest and Apoptosis
Source: Molecules. 2017 Sep 29;22(10):1635. doi: 10.3390/molecules22101635 (PMC6151802; doi:10.3390/molecules22101635)
Supplement: Supplementary file 1 [file molecules-22-01635-s001.pdf]

Supporting Information

A new series of cytotoxic pyrazoline derivatives as potential anticancer agents induces cell cycle arrest and apoptosis

Hong Wang [1,†], Jinhong Zheng [1,†], Weijie Xu [1], Cheng Chen [1], Duncan Wei [2], Wenxiu Ni [1,*] and Ying Pan [1,*]

[1] Department of Chemistry, Shantou University Medical College, Shantou 515041, Guangdong, China; 13592851552@163.com (H.W.); jhzheng@stu.edu.cn (J.Z.); 13536920534@163.com (W.X.); 13411963192@163.com (C.C.)

[2] Department of pharmacy, The First Affiliated Hospital of Shantou University Medical College, Shantou 515041, Guangdong, China; weiduncan2012@163.com

* Correspondence: wxni@stu.edu.cn; ypan@stu.edu.cn; Tel.: +86-754-8890-0499; Fax: +86-754-8855-7562.

† These authors contributed equally to this work.

Contents

Figure S1. The representative ^{1}H NMR of all compounds **1b-12b**.

^{1}H NMR spectrum of compounds 1b-12b (400 MHz, DMSO)

1b

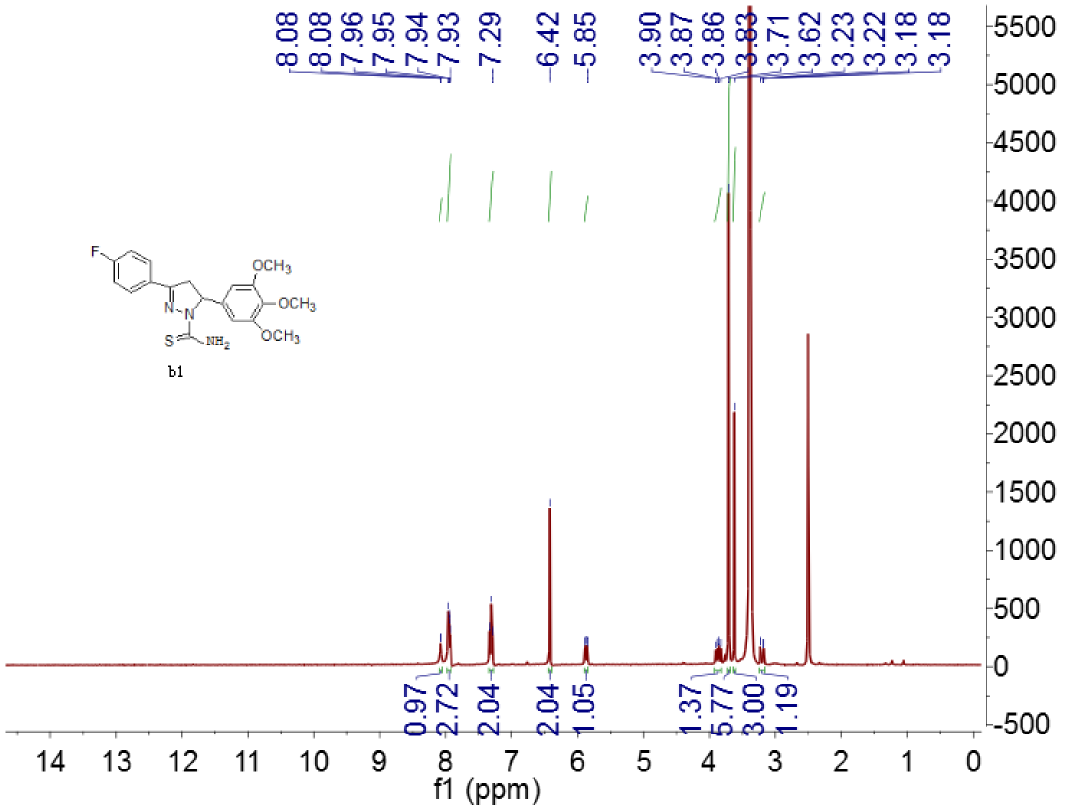

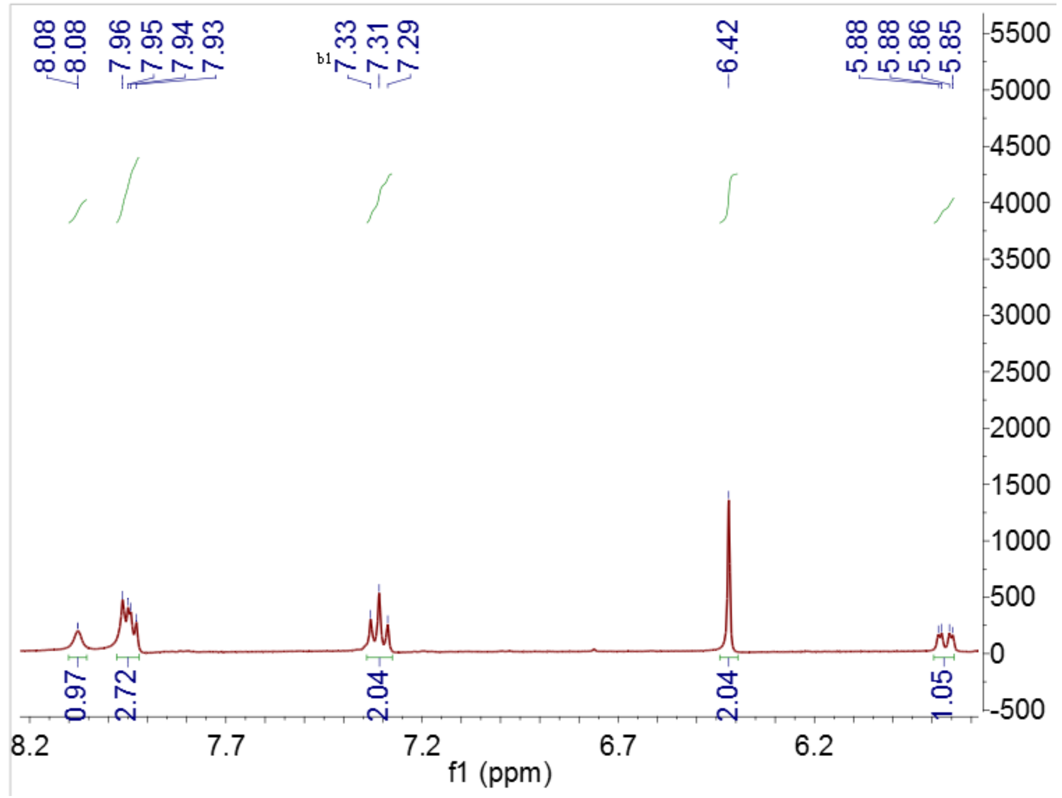

8.08
8.08
7.96
7.95
7.94
7.93
b1
7.33
7.31
7.29
6.42
5.88
5.88
5.86
5.85
0.97
2.72
2.04
2.04
1.05
f1 (ppm)

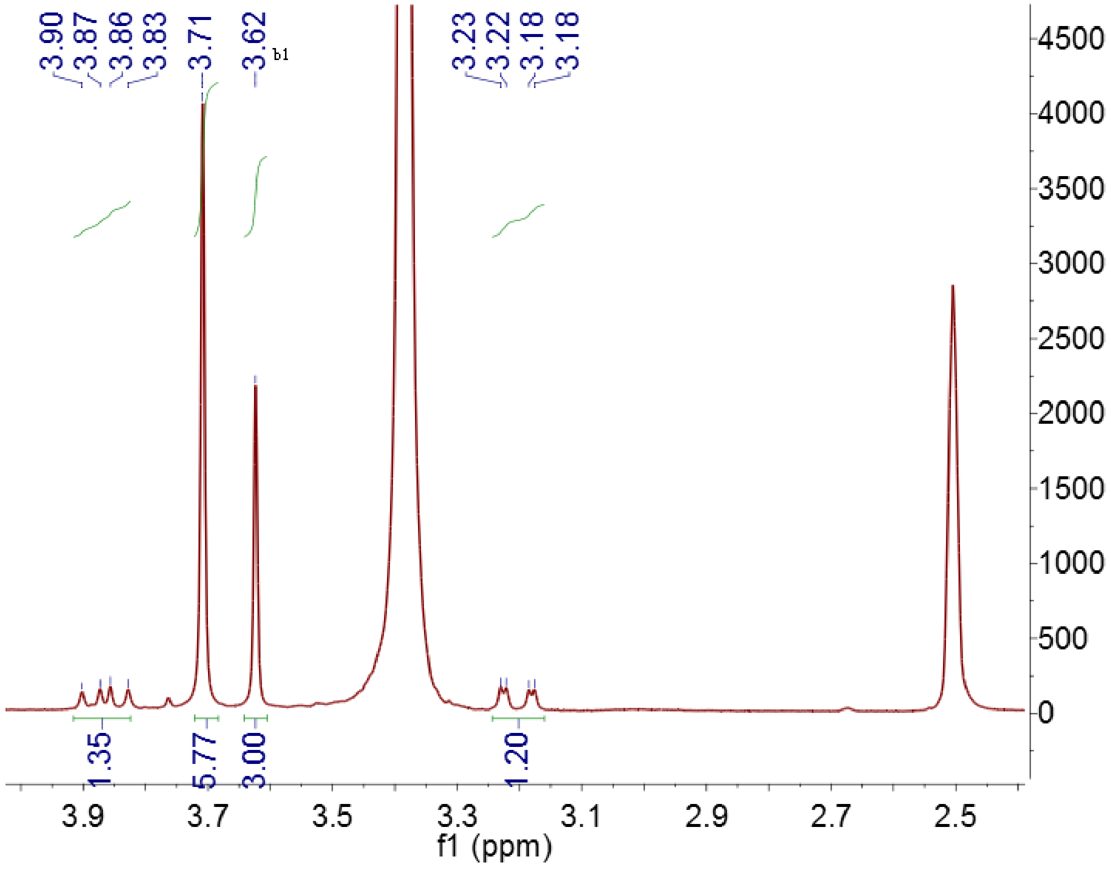

3.90
3.87
3.86
3.83
3.71
3.62
b1
3.23
3.22
3.18
3.18
1.35
5.77
3.00
1.20
f1 (ppm)

2b

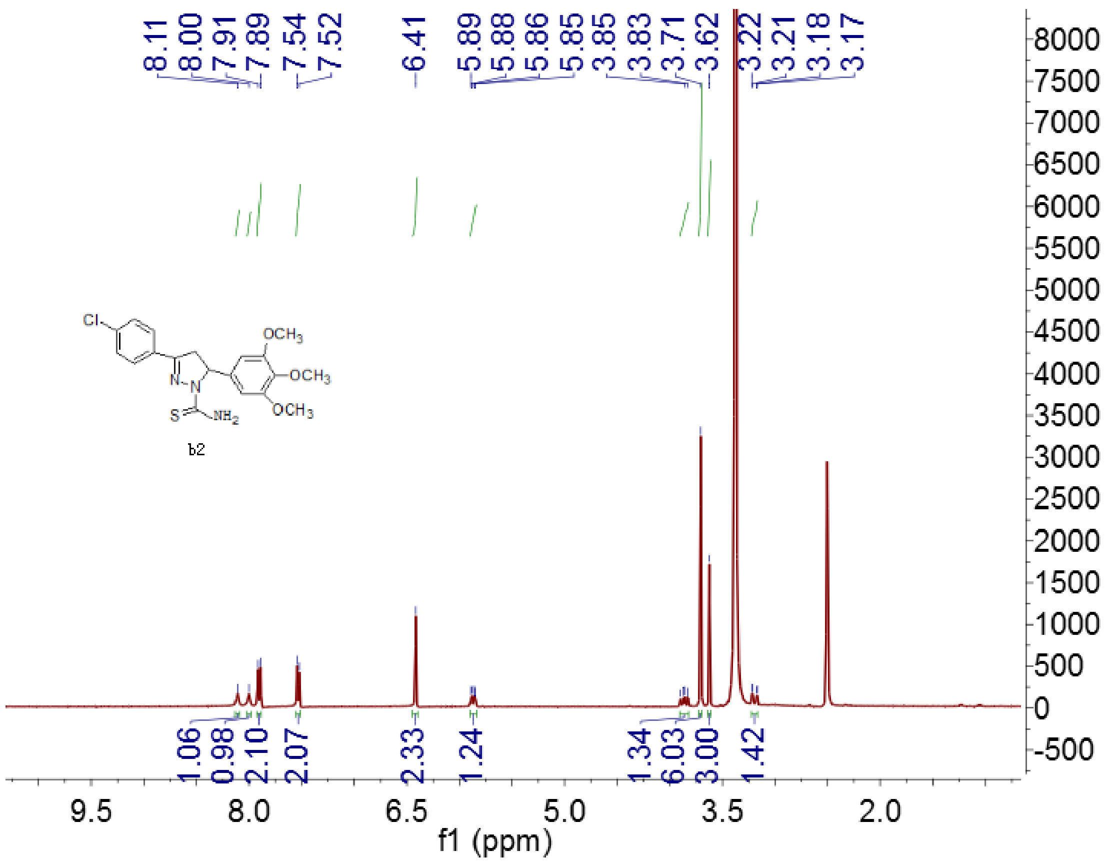
8.11 8.00 7.91 7.89 7.54 7.52 6.41 5.89 5.88 5.86 5.85 3.85 3.83 3.71 3.62 3.22 3.21 3.18 3.17
Cl
N
N
S
NH2
OCH3
OCH3
OCH3
b2
1.06 0.98 2.10 2.07 2.33 1.24 1.34 6.03 3.00 1.42
9.5 8.0 6.5 5.0 3.5 2.0
f1 (ppm)

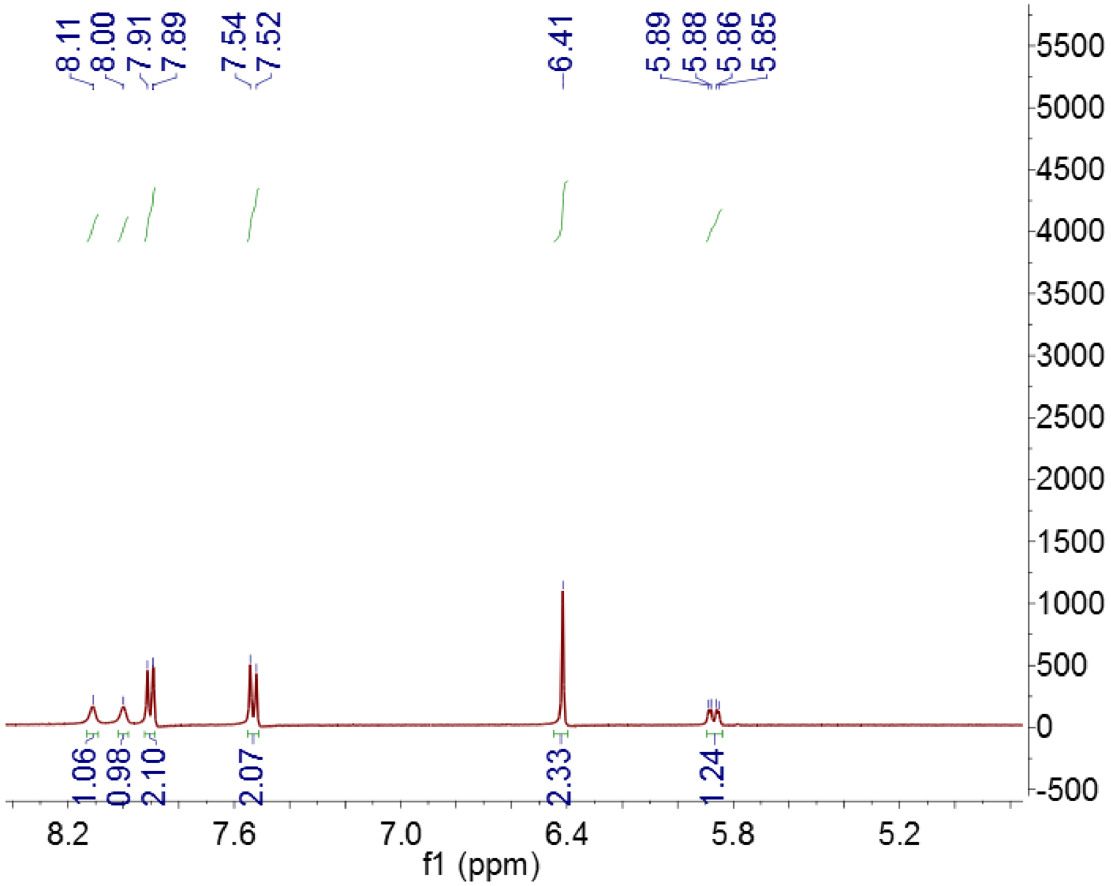
8.11 8.00 7.91 7.89 7.54 7.52 6.41 5.89 5.88 5.86 5.85
1.06 0.98 2.10 2.07 2.33 1.24
8.2 7.6 7.0 6.4 5.8 5.2
f1 (ppm)

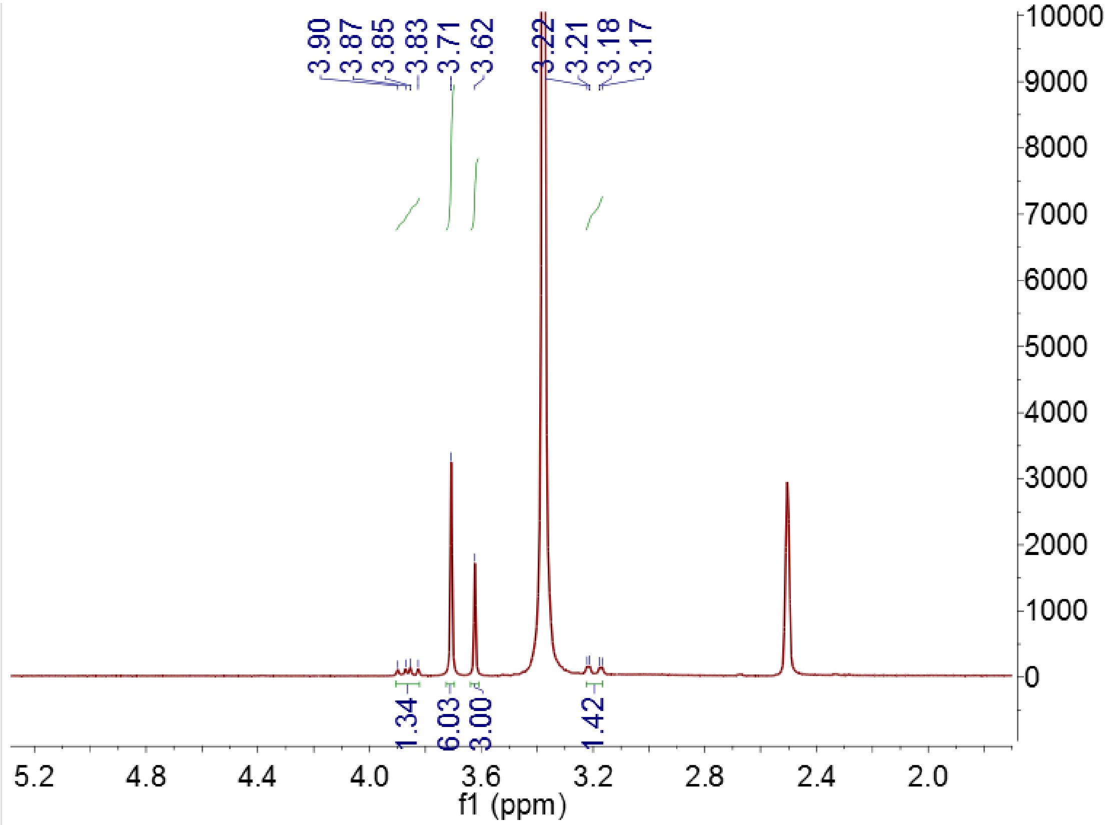

3b

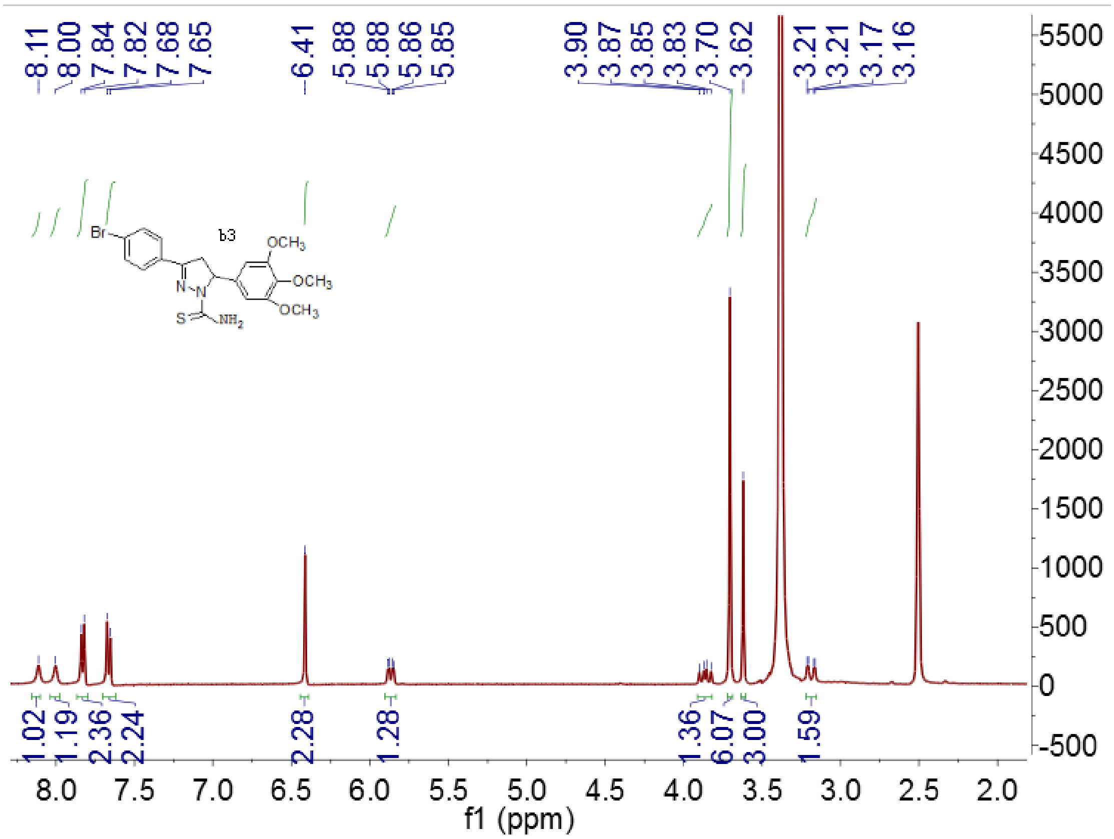

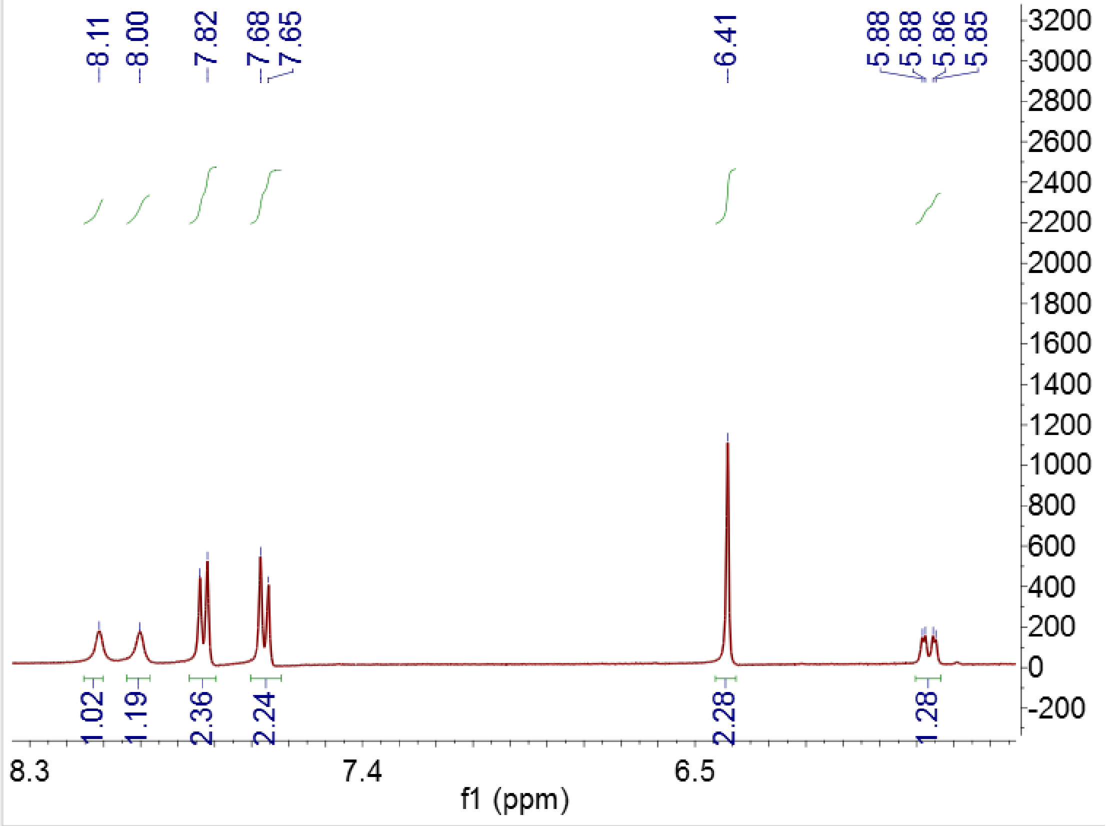

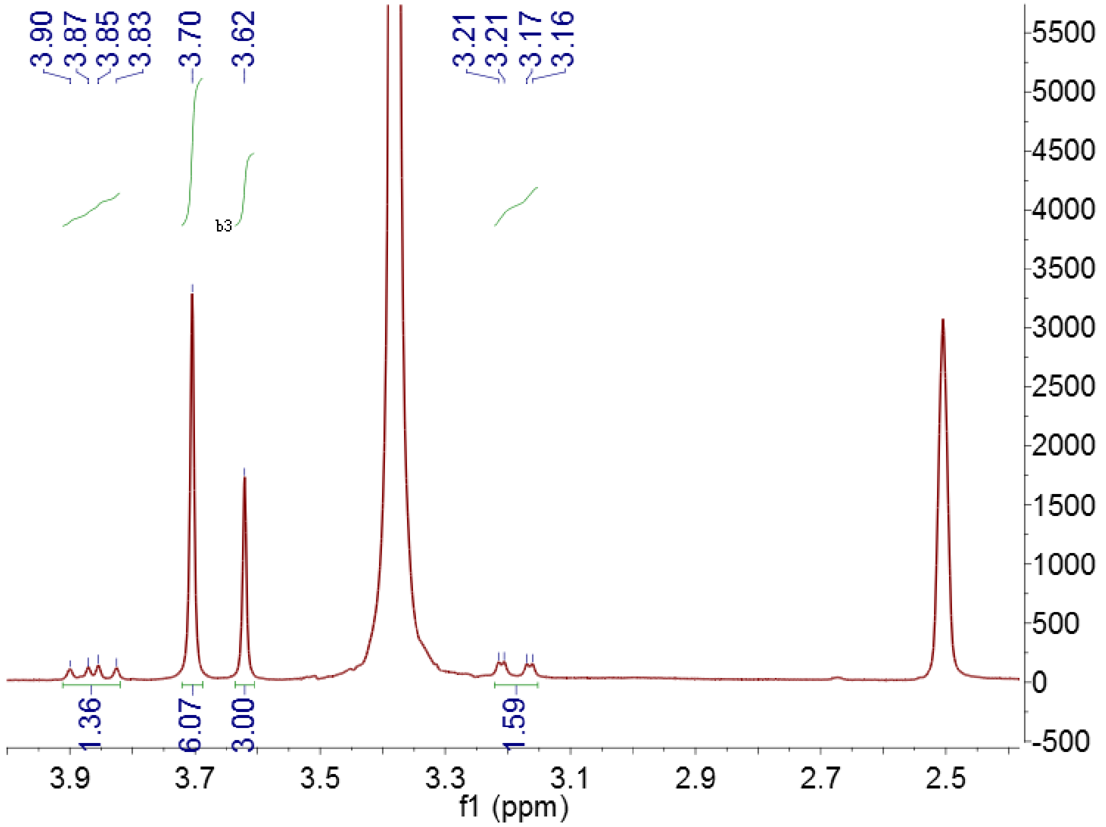

4b

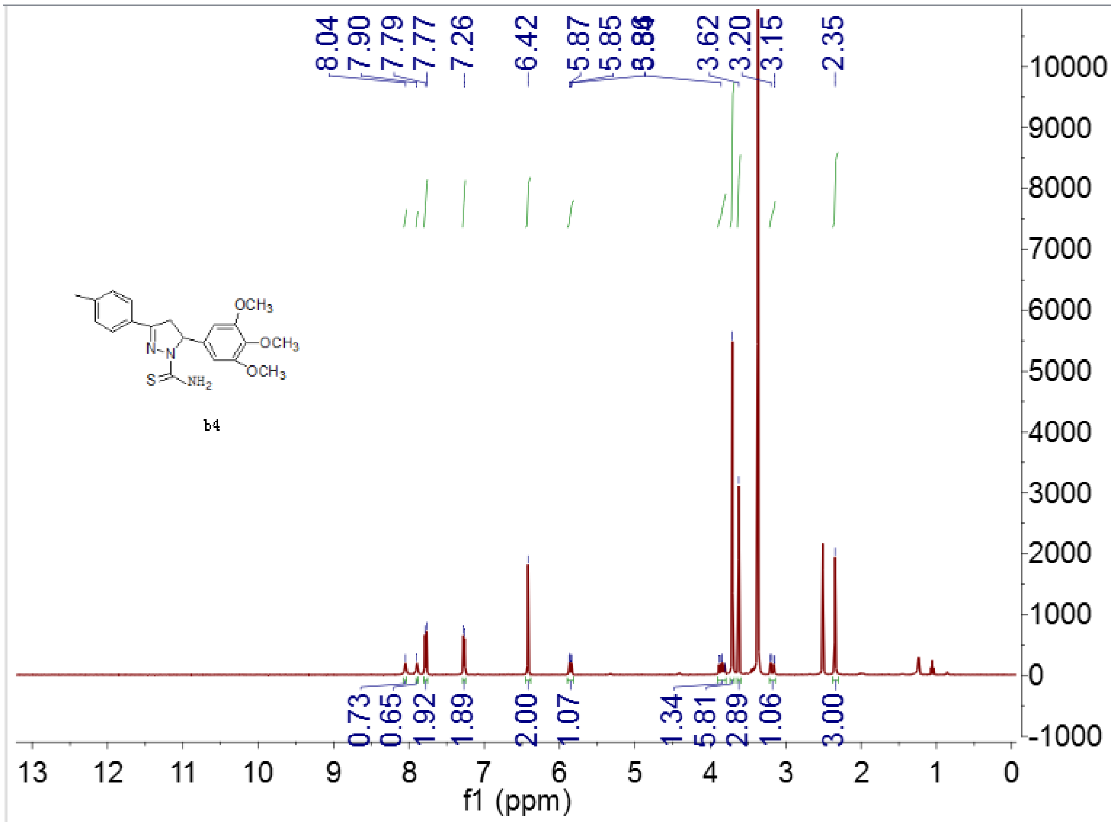
8.04 7.90 7.79 7.77 7.26 6.42 5.87 5.85 5.84 3.62 3.20 3.15 2.35
0.73 0.65 1.92 1.89 2.00 1.07 1.34 5.81 2.89 1.06 3.00
OCH3
OCH3
OCH3
NH2
b4
f1 (ppm)

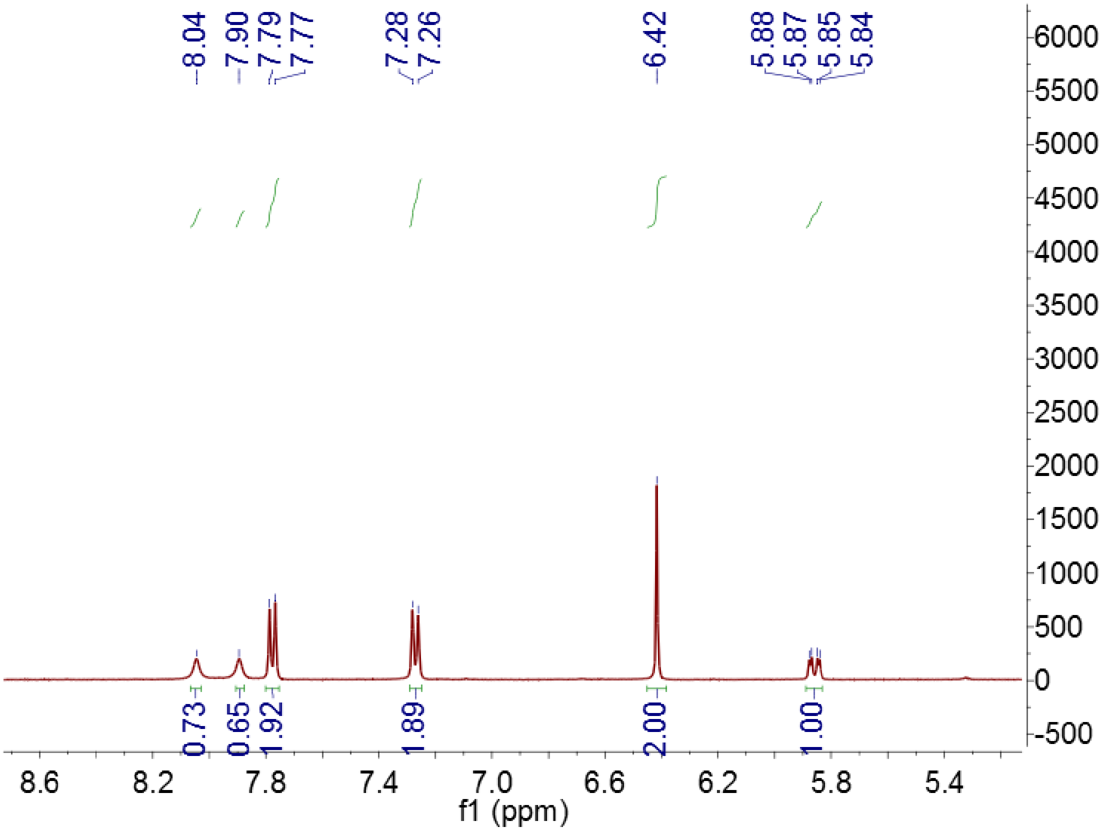
8.04 7.90 7.79 7.77 7.28 7.26 6.42 5.88 5.87 5.85 5.84
0.73 0.65 1.92 1.89 2.00 1.00
f1 (ppm)

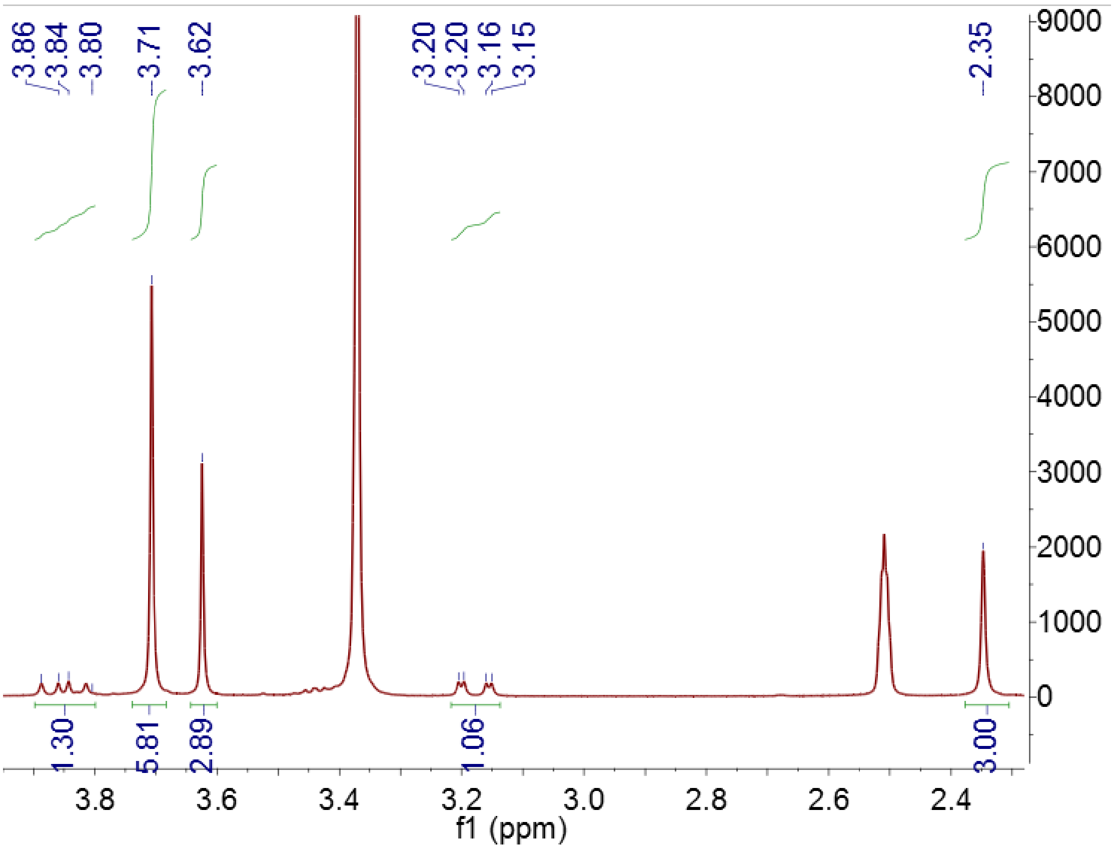
3.86 3.84 3.80 3.71 3.62 3.20 3.20 3.16 3.15 2.35
1.30 5.81 2.89 1.06 3.00
f1 (ppm)

5b

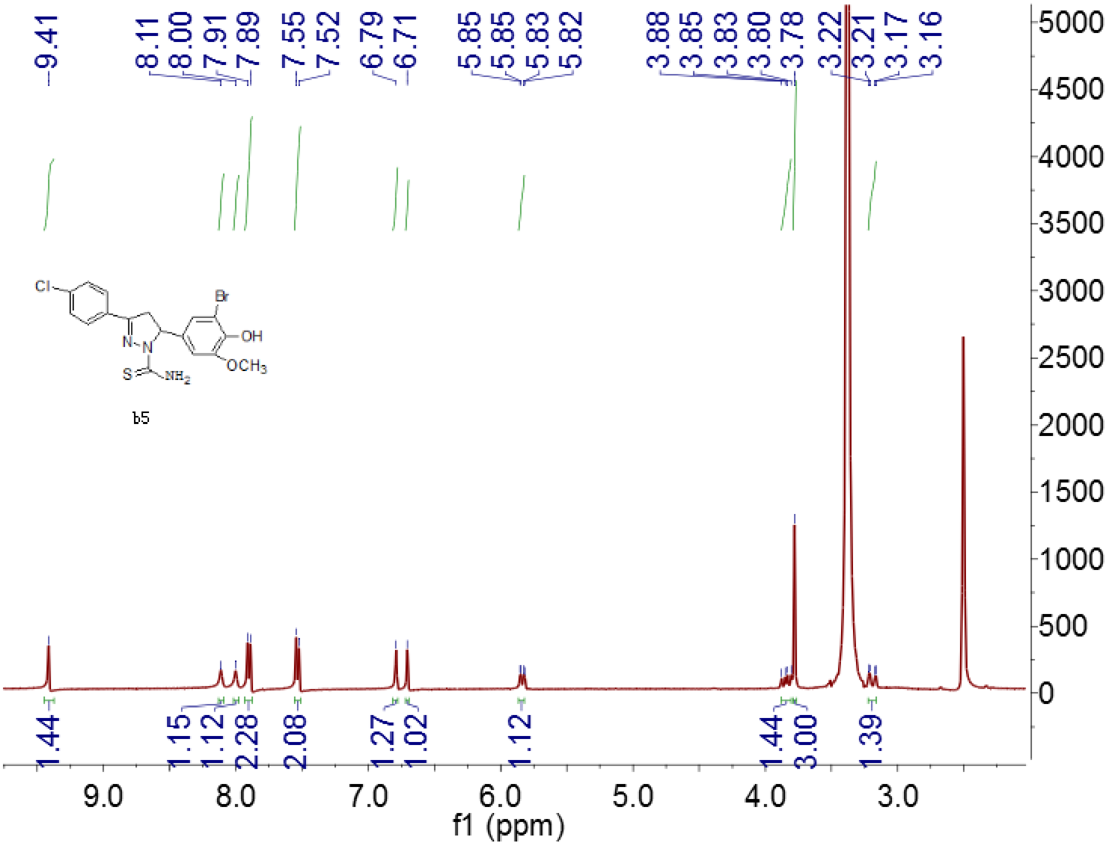
9.41 8.11 8.00 7.91 7.89 7.55 7.52 6.79 6.71 5.85 5.85 5.83 5.82 3.88 3.85 3.83 3.80 3.78 3.22 3.21 3.17 3.16
1.44 1.15 1.12 2.28 2.08 1.27 1.02 1.12 1.44 3.00 1.39
Cl
Br
OH
OCH3
N
S
NH2
b5
f1 (ppm)

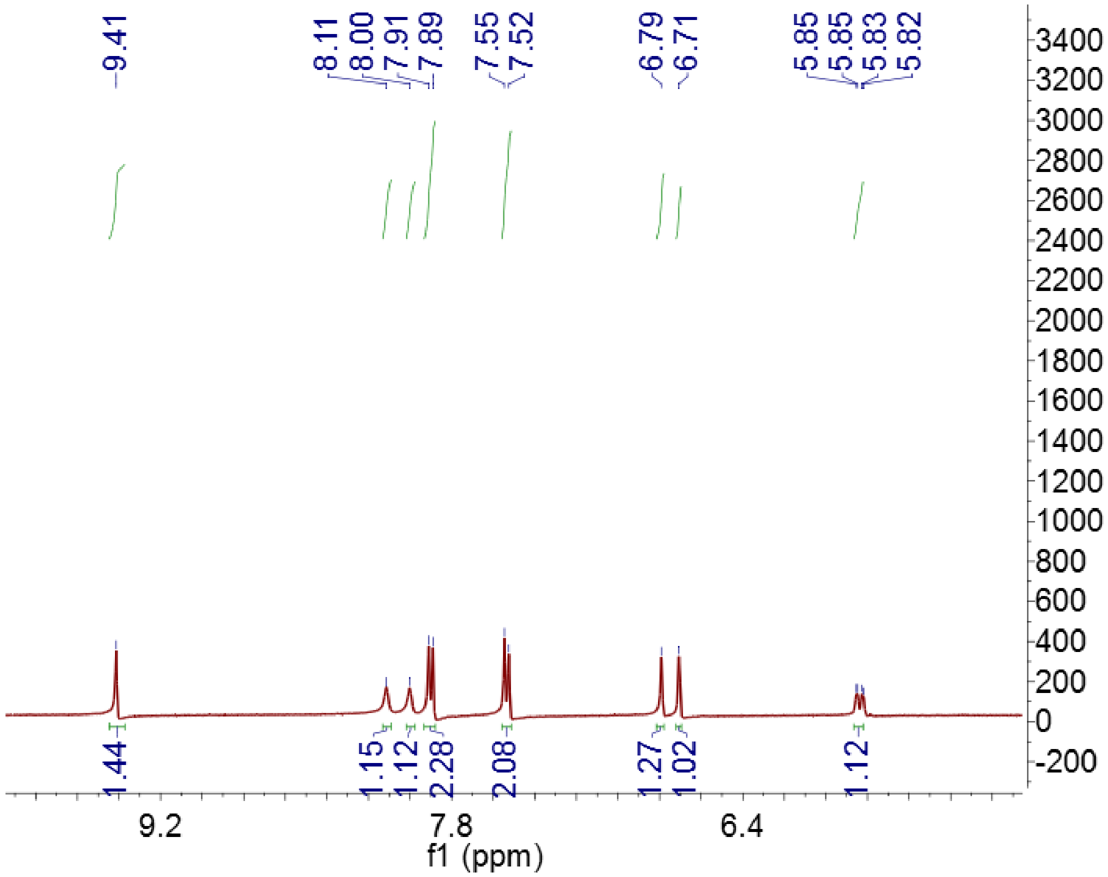
9.41
8.11
8.00
7.91
7.89
7.55
7.52
6.79
6.71
5.85
5.85
5.83
5.82
1.44
1.15
1.12
2.28
2.08
1.27
1.02
1.12
9.2
7.8
6.4
f1 (ppm)

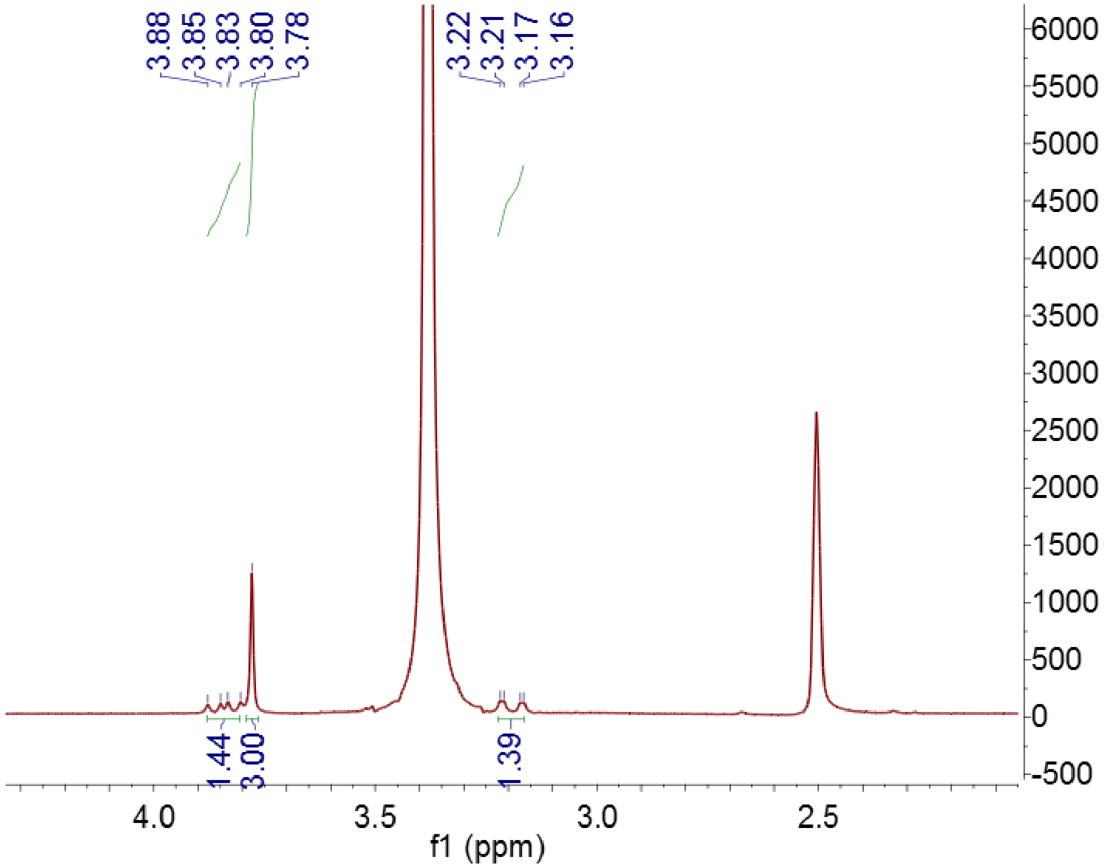
3.88
3.85
3.83
3.80
3.78
3.22
3.21
3.17
3.16
1.44
3.00
1.39
4.0
3.5
3.0
2.5
f1 (ppm)

6b

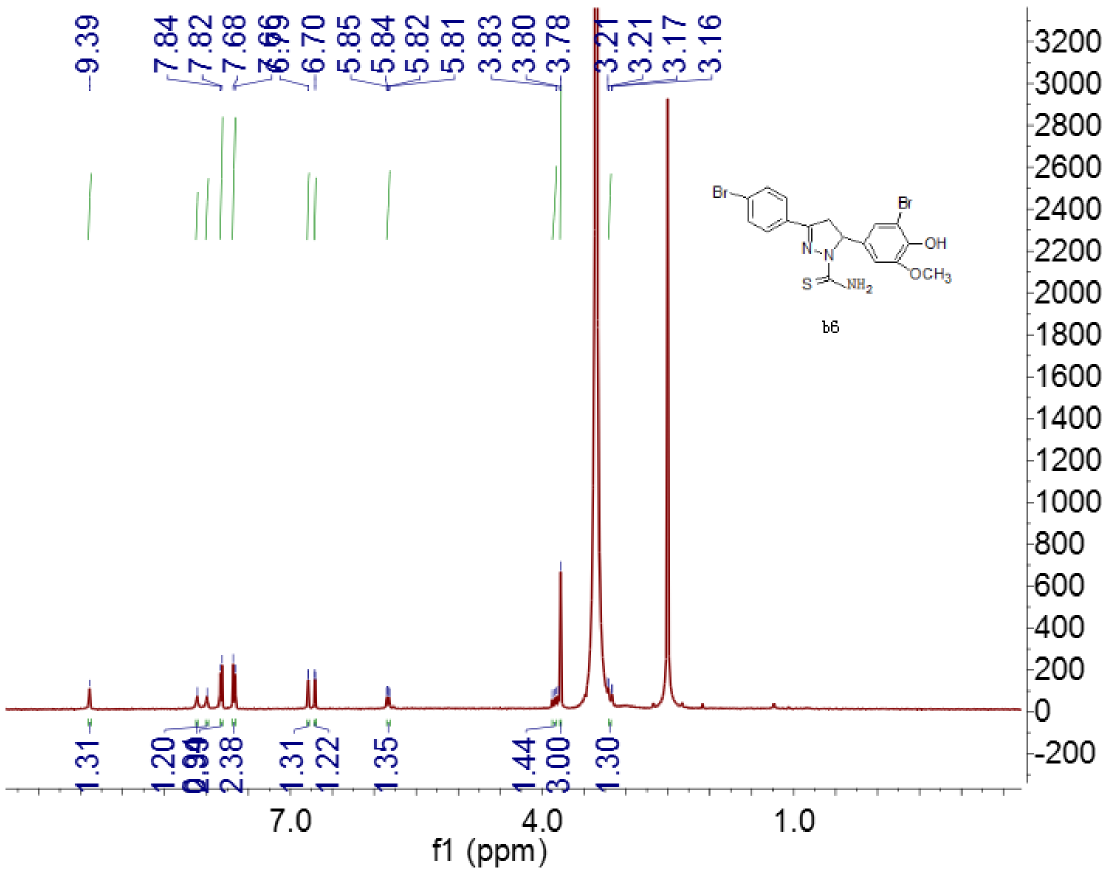
9.39
7.84 7.82 7.68 7.66 6.79 6.70 5.85 5.84 5.82 5.81 3.83 3.80 3.78 3.21 3.21 3.17 3.16
Br
Br
OH
N
N
OCH3
S
NH2
b6
1.31 1.20 0.94 2.39 2.38 1.31 1.22 1.35 1.44 3.00 1.30
7.0
4.0
1.0
f1 (ppm)
3200
3000
2800
2600
2400
2200
2000
1800
1600
1400
1200
1000
800
600
400
200
0
-200

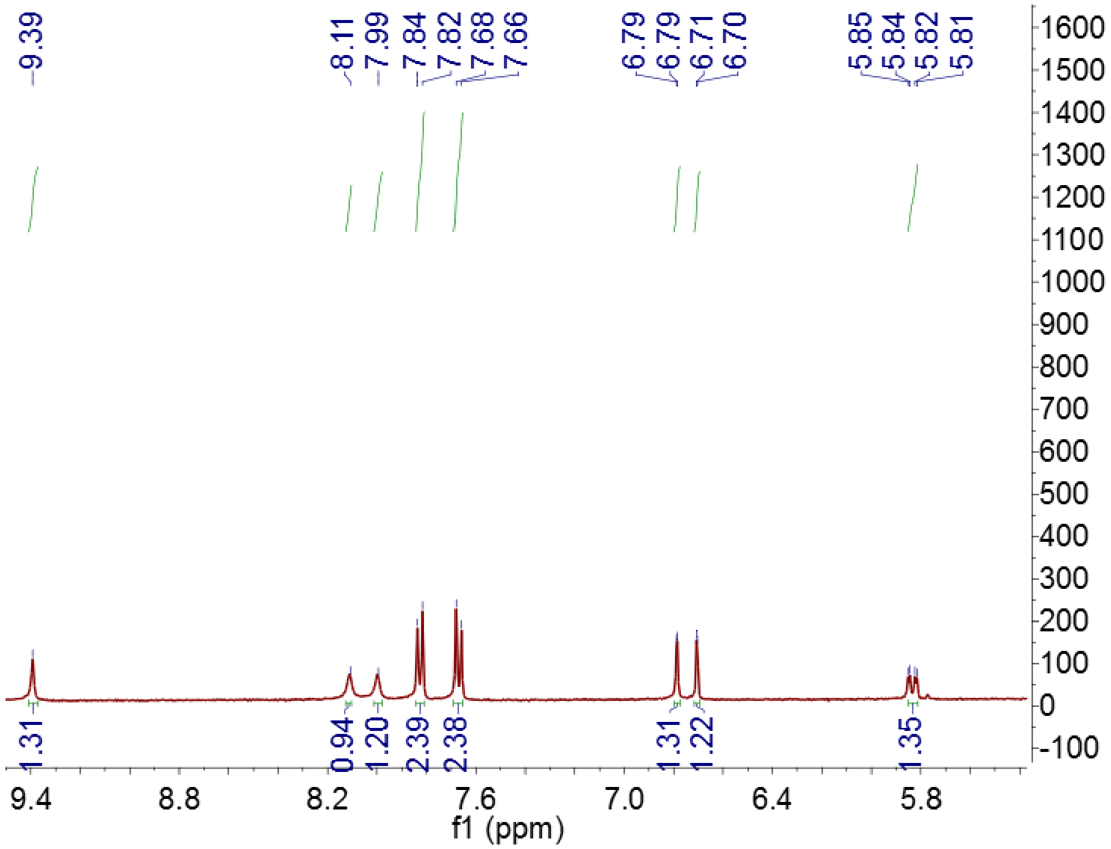
9.39
8.11 7.99 7.84 7.82 7.68 7.66
6.79 6.79 6.71 6.70
5.85 5.84 5.82 5.81
1.31 0.94 1.20 2.39 2.38 1.31 1.22 1.35
9.4
8.8
8.2
7.6
7.0
6.4
5.8
f1 (ppm)
1600
1500
1400
1300
1200
1100
1000
900
800
700
600
500
400
300
200
100
0
-100

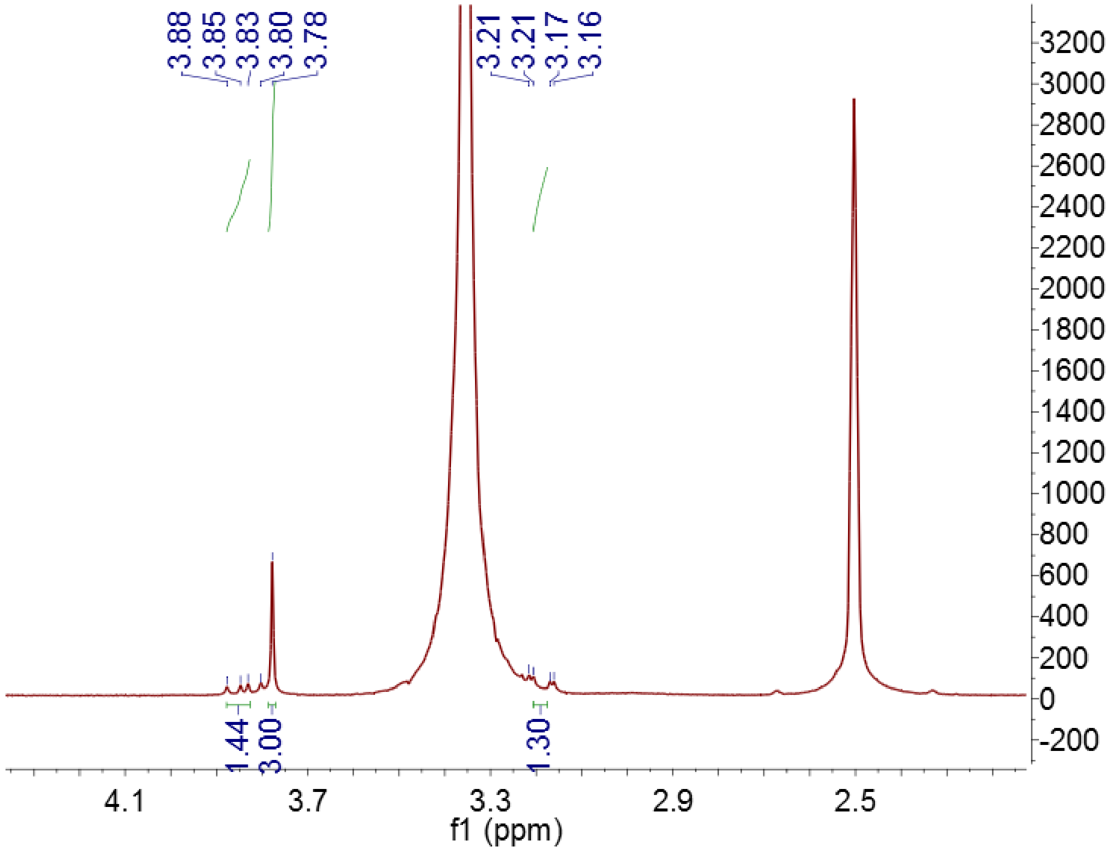

7b

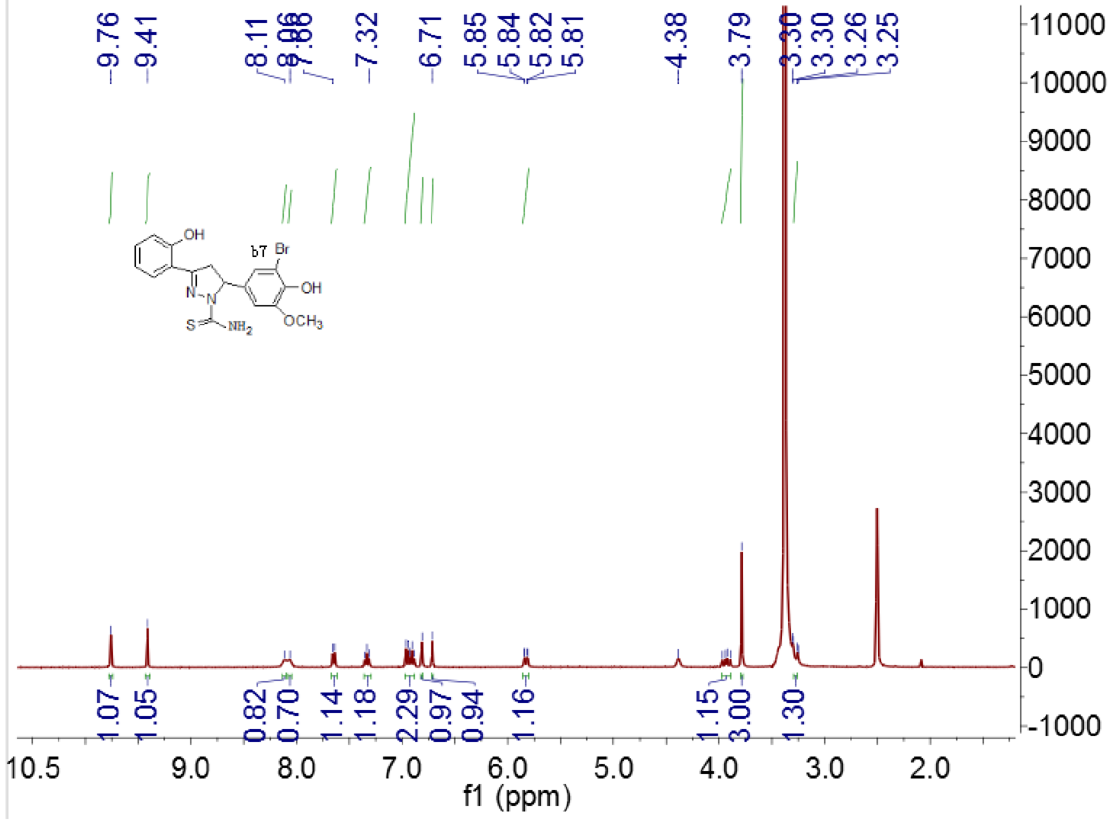

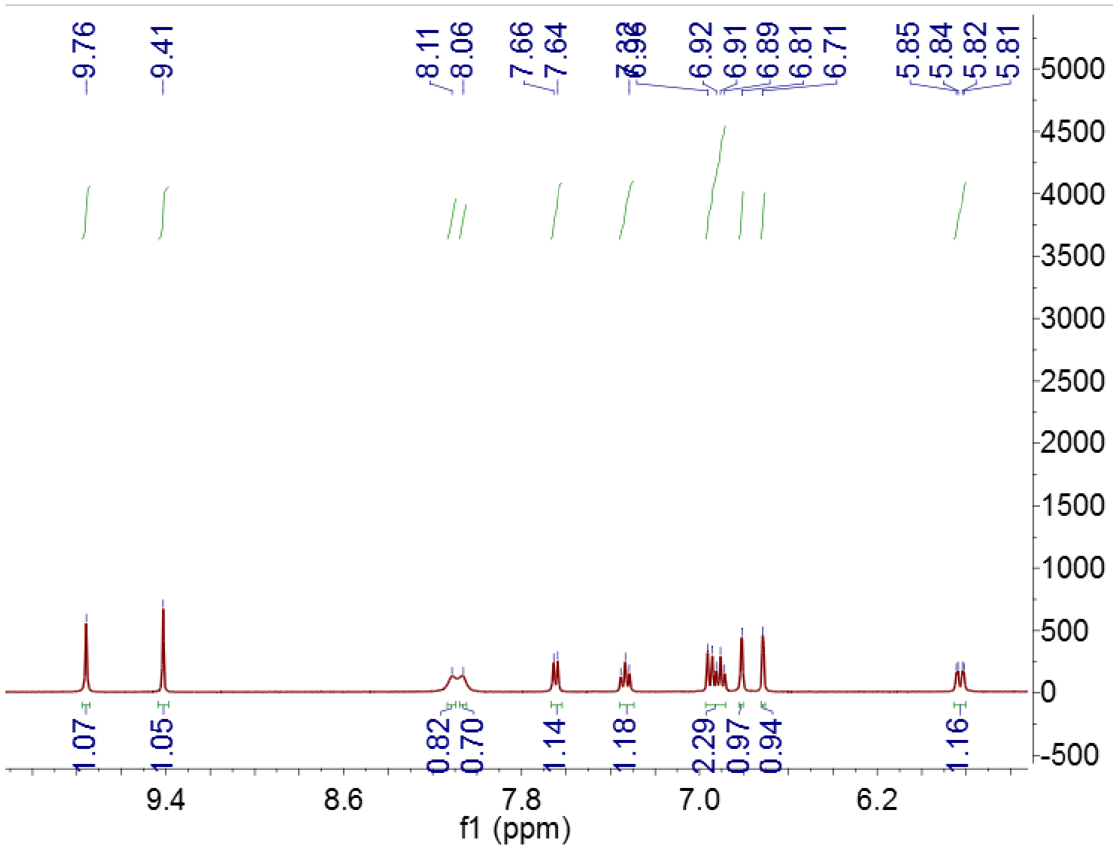

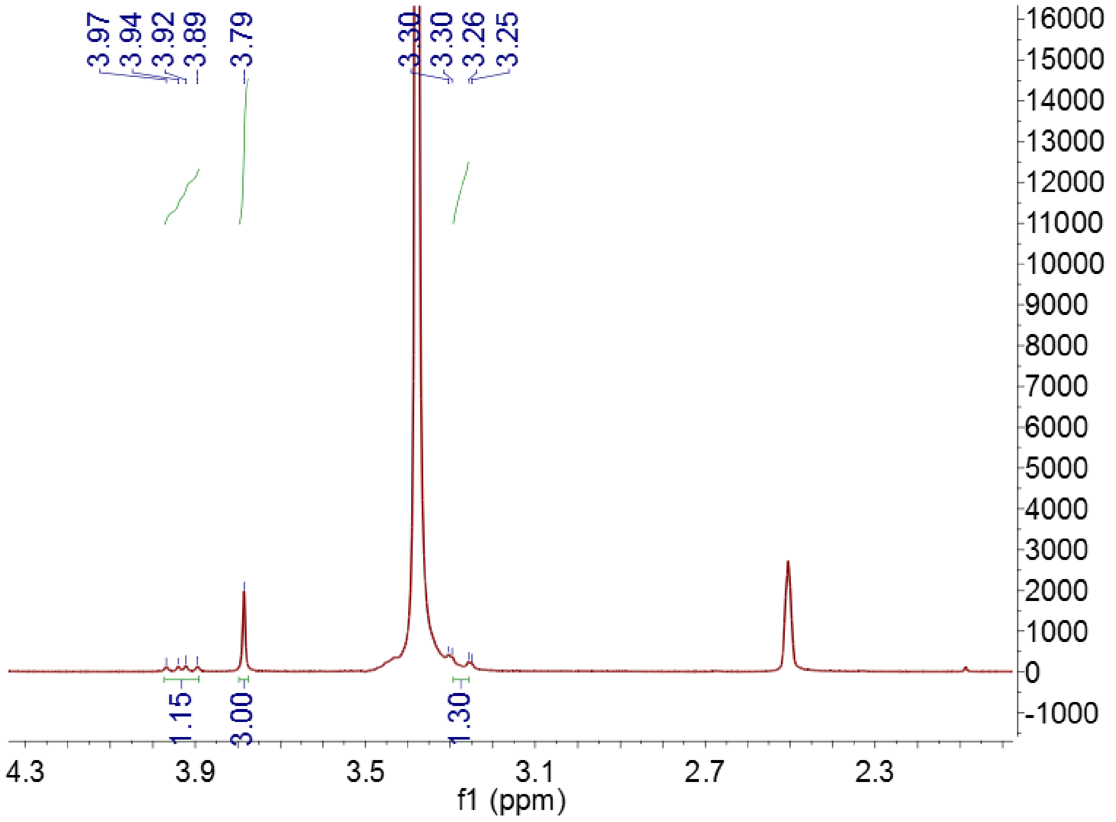

8b

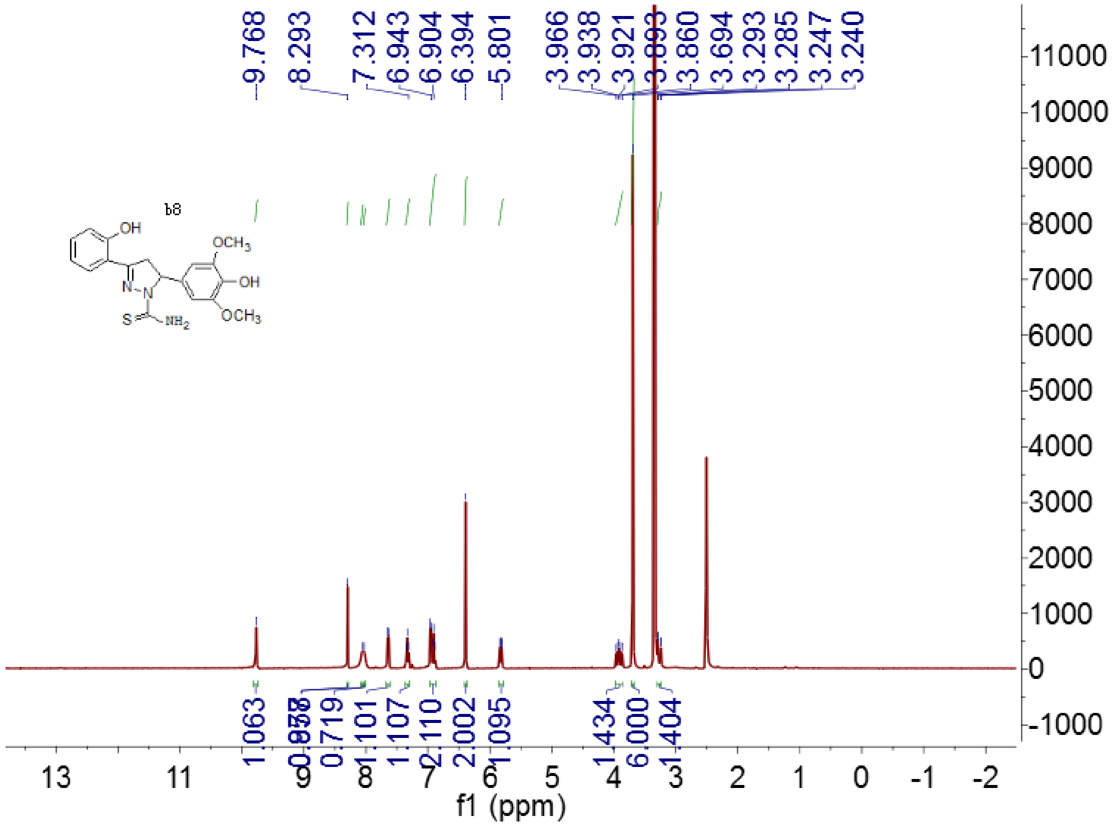
b8
OH
OCH3
OH
OCH3
N
N
S
NH2
9.768
8.293
7.312
6.943
6.904
6.394
5.801
3.966
3.938
3.921
3.860
3.694
3.293
3.285
3.247
3.240
1.063
0.719
1.101
1.107
2.110
2.002
1.095
1.434
6.000
1.404
f1 (ppm)

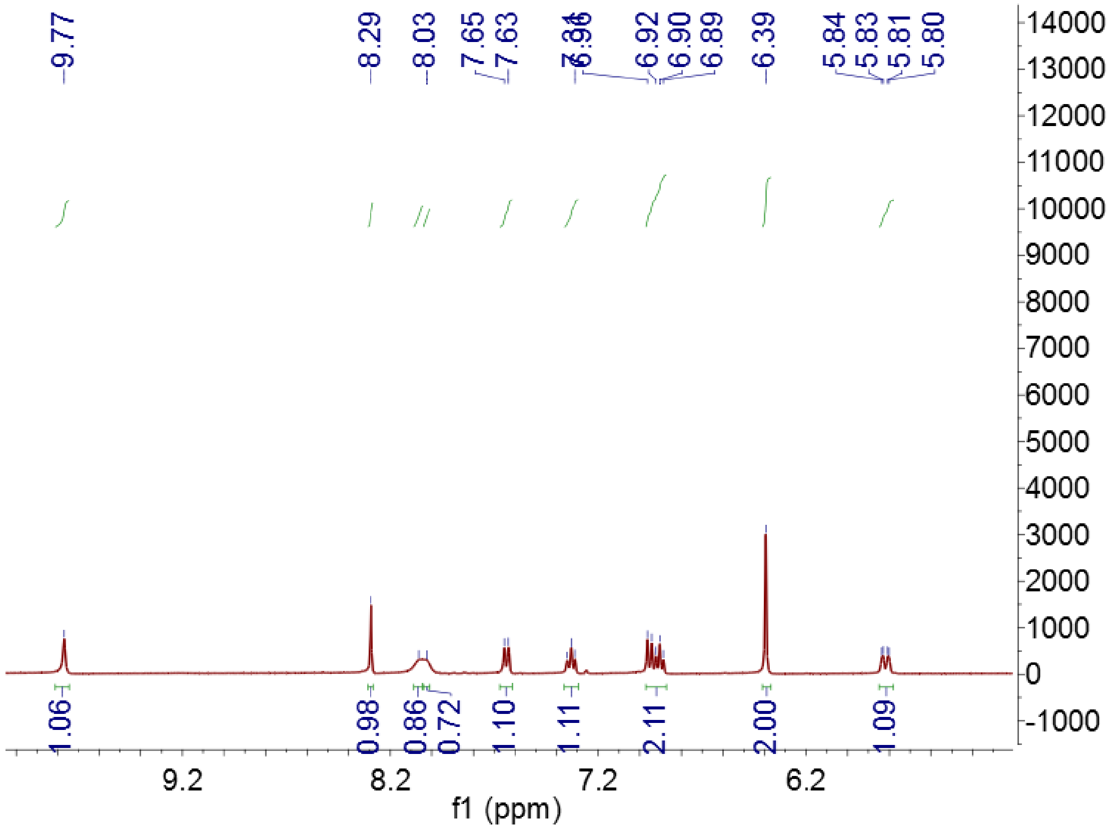
9.77
8.29
8.03
7.65
7.63
6.92
6.90
6.89
6.39
5.84
5.83
5.81
5.80
1.06
0.98
0.86
0.72
1.10
1.11
2.11
2.00
1.09
f1 (ppm)

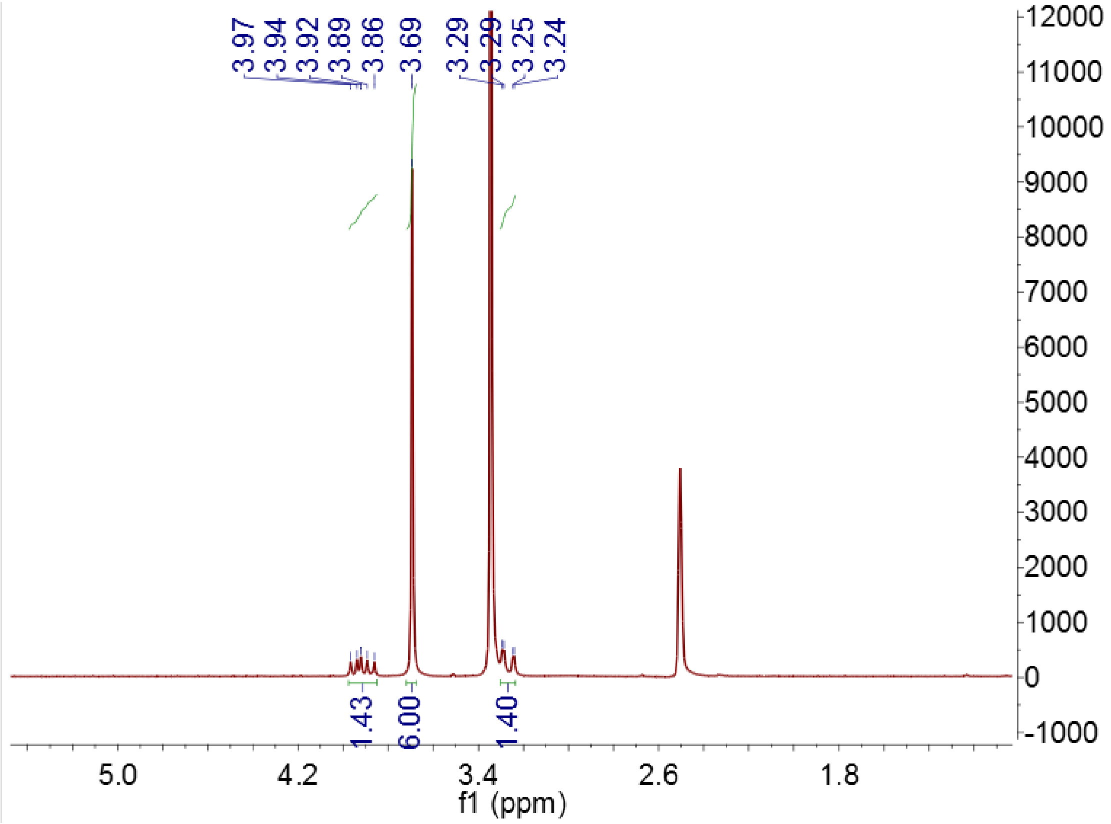
3.97
3.94
3.92
3.89
3.86
3.69
3.29
3.29
3.25
3.24
1.43
6.00
1.40
f1 (ppm)

9b

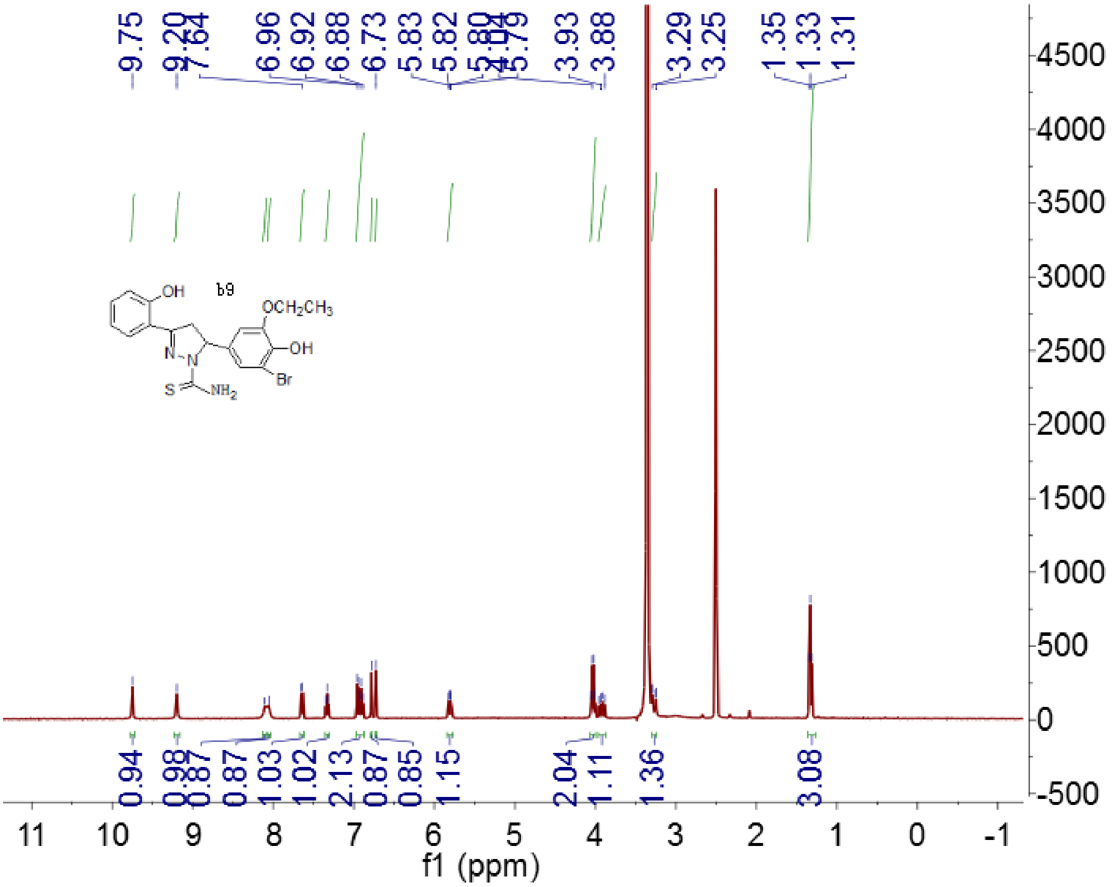
9.75
9.20
7.64
6.96
6.92
6.88
6.73
5.83
5.82
5.80
5.79
3.93
3.88
3.29
3.25
1.35
1.33
1.31
OH
b9
OCH2CH3
OH
Br
N
N
S
NH2
0.94
0.98
0.87
0.87
1.03
1.02
2.13
0.87
0.85
1.15
2.04
1.11
1.36
3.08
f1 (ppm)

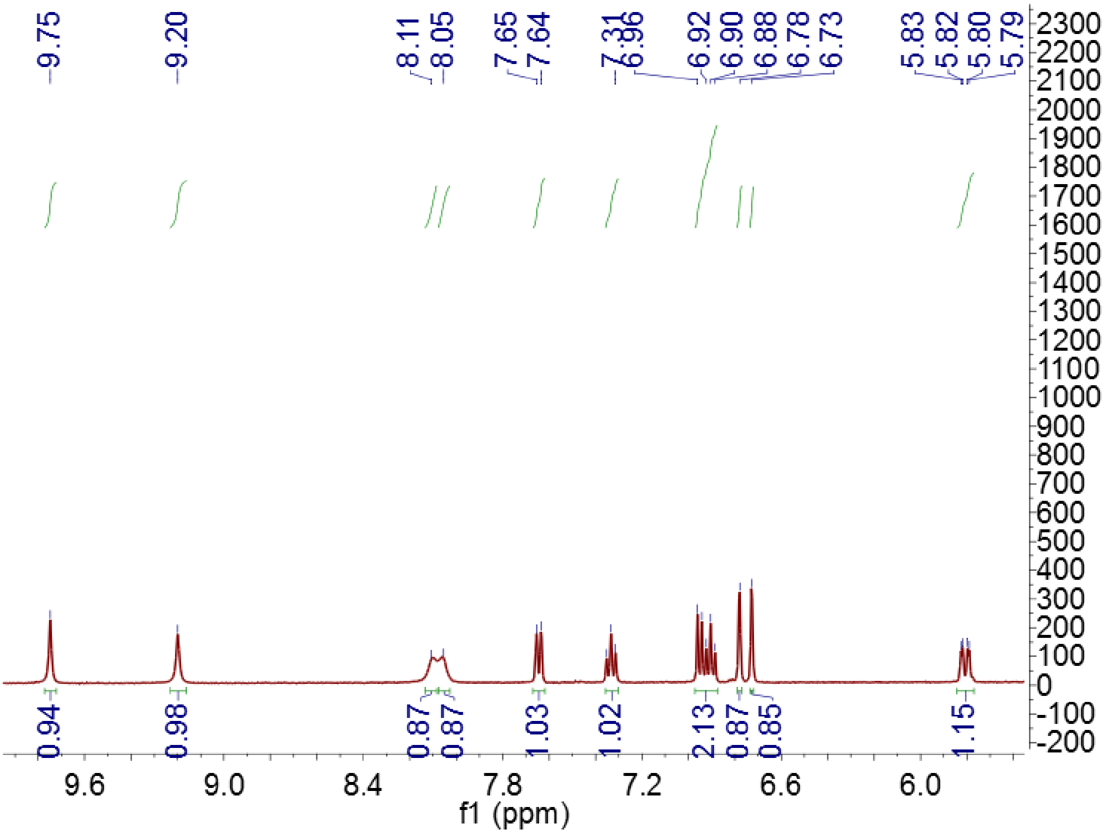
9.75
9.20
8.11
8.05
7.65
7.64
7.31
6.96
6.92
6.90
6.88
6.78
6.73
5.83
5.82
5.80
5.79
0.94
0.98
0.87
0.87
1.03
1.02
2.13
0.87
0.85
1.15
9.6
9.0
8.4
7.8
7.2
6.6
6.0
f1 (ppm)

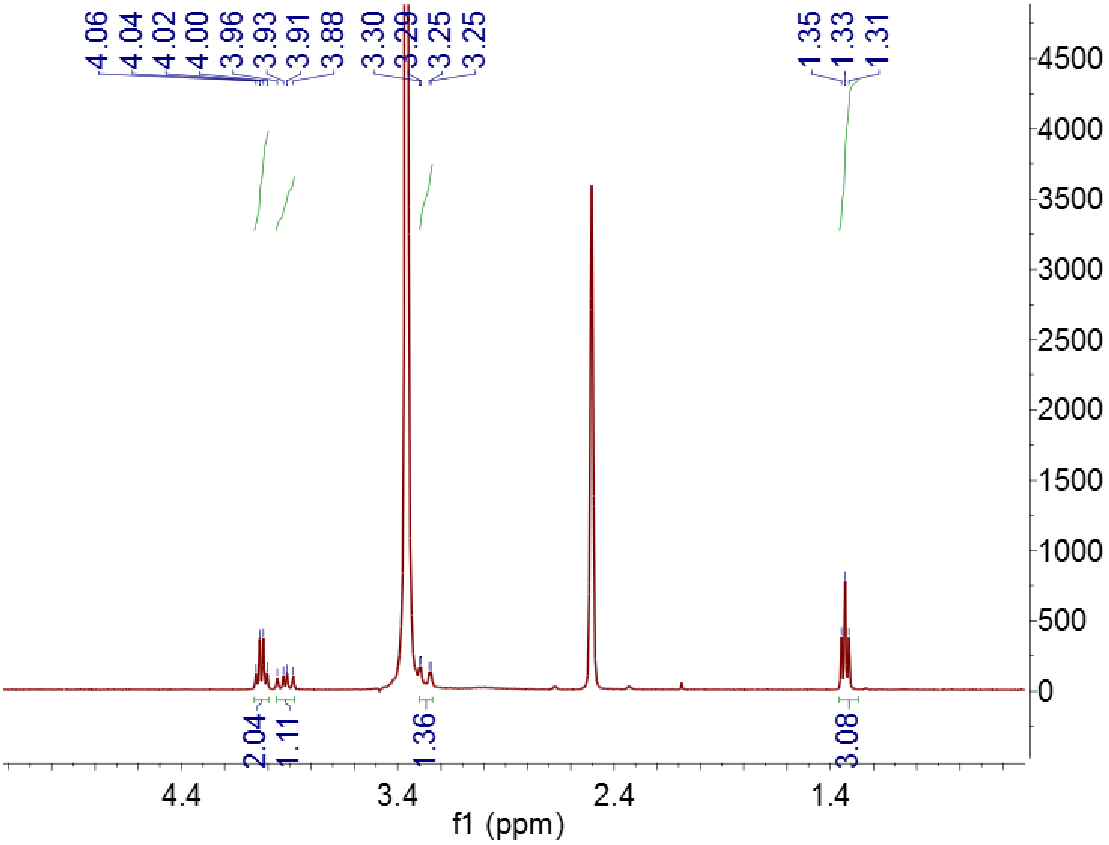
4.06
4.04
4.02
4.00
3.96
3.93
3.91
3.88
3.30
3.29
3.25
3.25
1.35
1.33
1.31
2.04
1.11
1.36
3.08
4.4
3.4
2.4
1.4
f1 (ppm)

10b

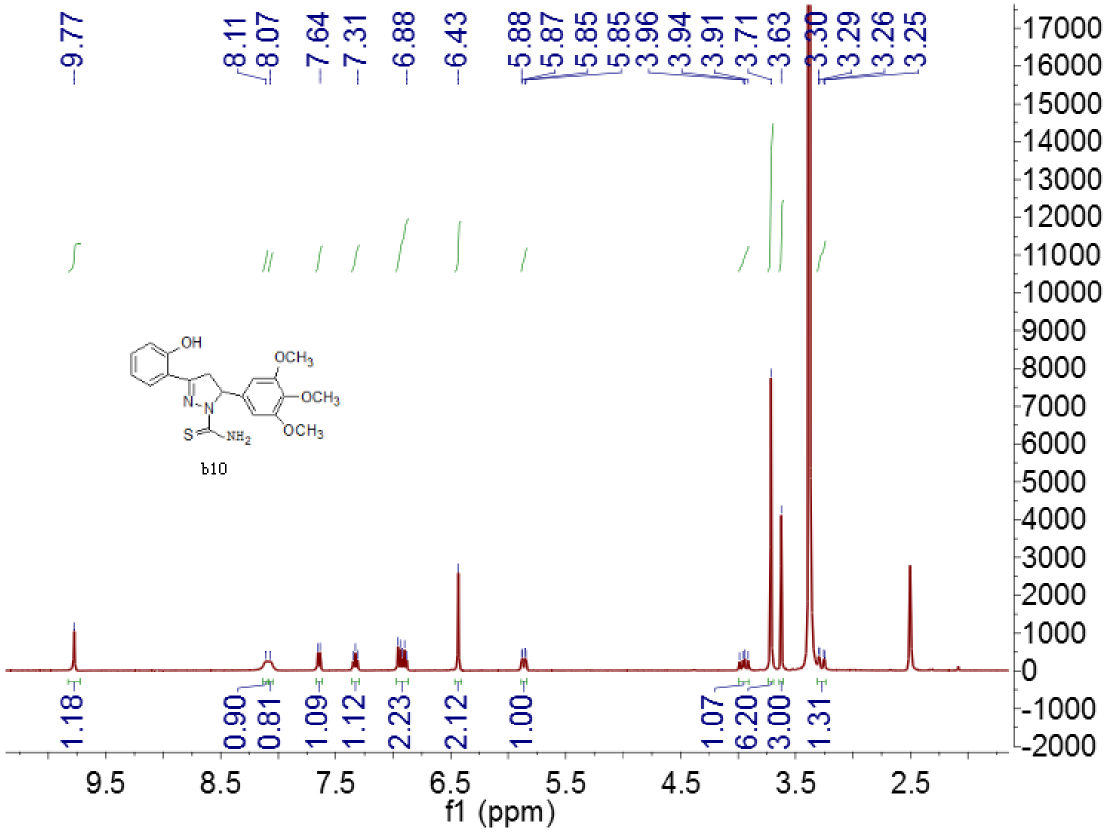
9.77
8.11
8.07
7.64
7.31
6.88
6.43
5.88
5.87
5.85
5.85
3.96
3.94
3.91
3.71
3.63
3.30
3.29
3.26
3.25
OH
OCH3
OCH3
OCH3
N
N
S
NH2
b10
1.18
0.90
0.81
1.09
1.12
2.23
2.12
1.00
1.07
6.20
3.00
1.31
17000
16000
15000
14000
13000
12000
11000
10000
9000
8000
7000
6000
5000
4000
3000
2000
1000
0
-1000
-2000
9.5
8.5
7.5
6.5
5.5
4.5
3.5
2.5
f1 (ppm)

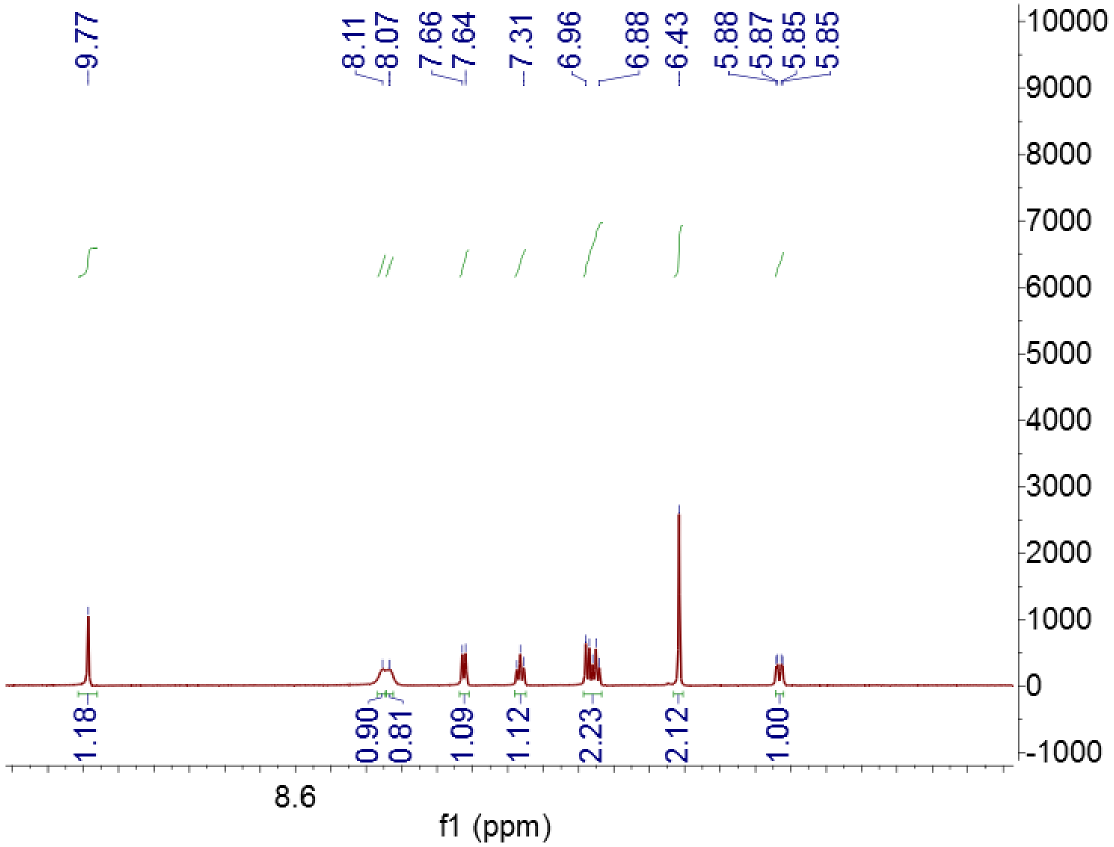
9.77
8.11
8.07
7.66
7.64
7.31
6.96
6.88
6.43
5.88
5.87
5.85
5.85
1.18
0.90
0.81
1.09
1.12
2.23
2.12
1.00
10000
9000
8000
7000
6000
5000
4000
3000
2000
1000
0
-1000
8.6
f1 (ppm)

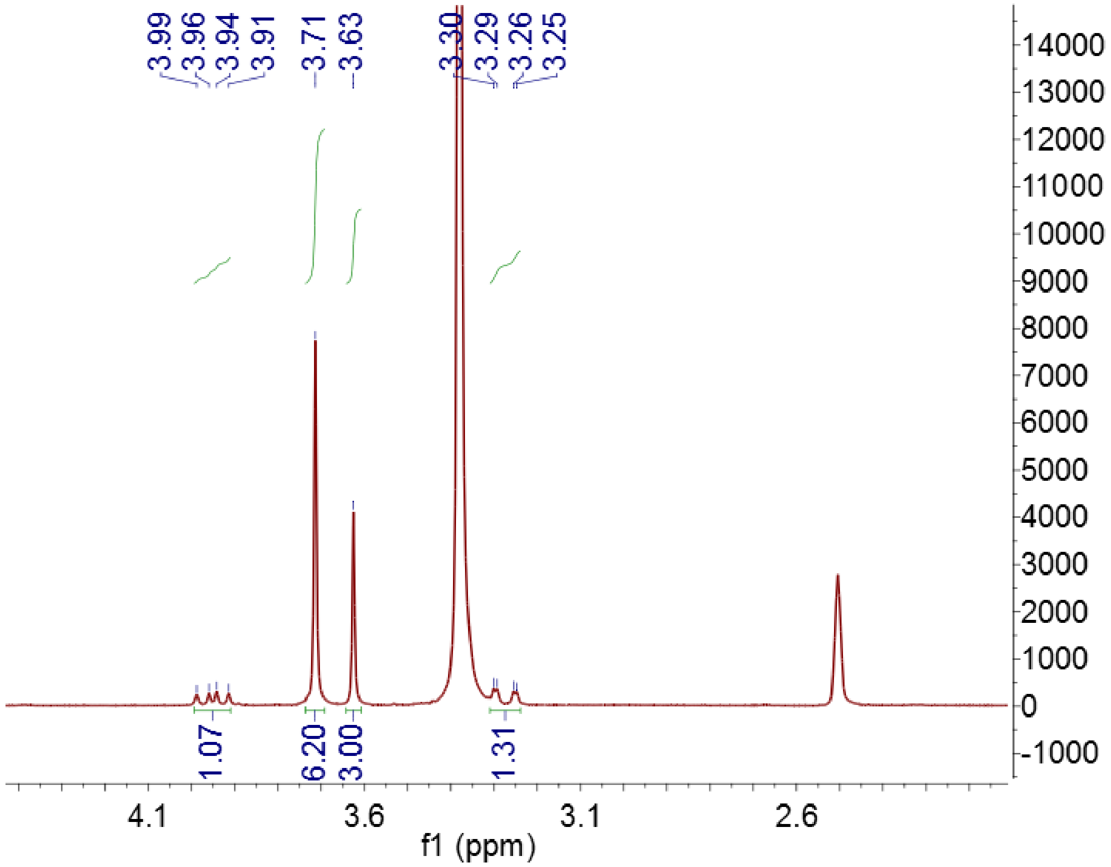
3.99
3.96
3.94
3.91
3.71
3.63
3.30
3.29
3.26
3.25
1.07
6.20
3.00
1.31
f1 (ppm)

11b

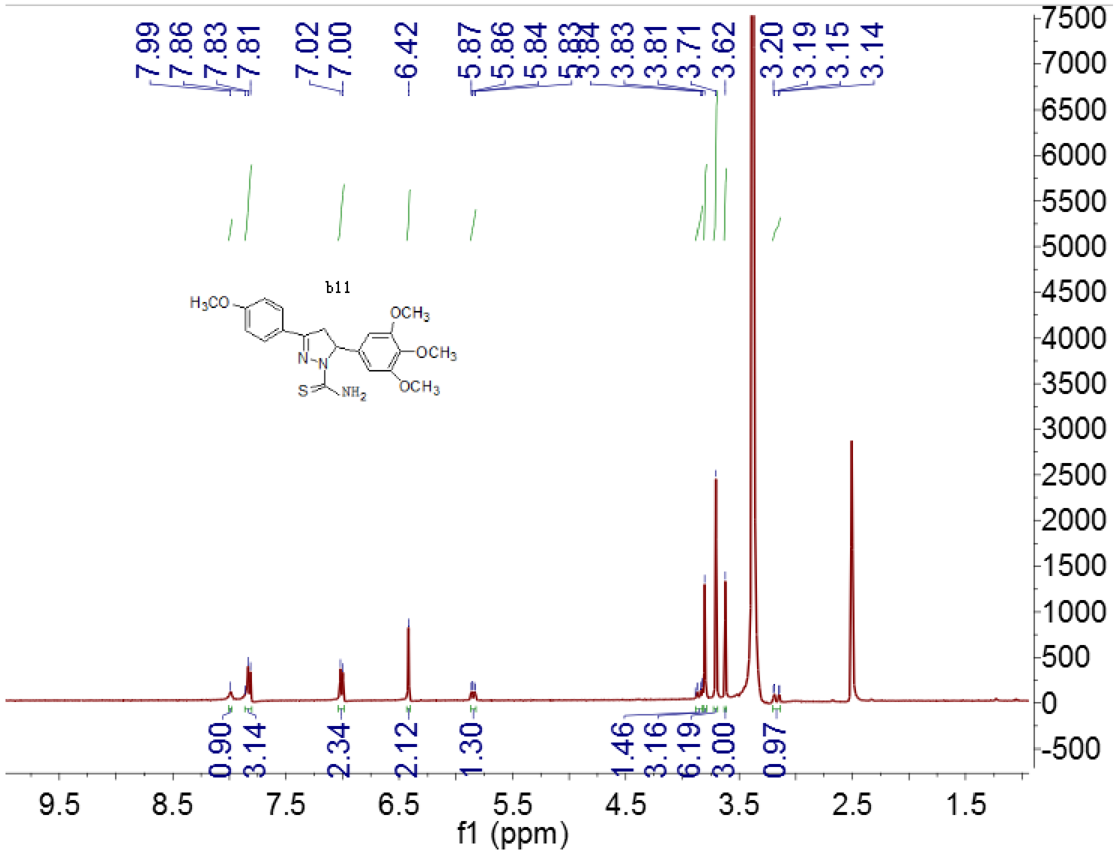
7.99
7.86
7.83
7.81
7.02
7.00
6.42
5.87
5.86
5.84
5.83
3.84
3.83
3.81
3.71
3.62
3.20
3.19
3.15
3.14
b11
0.90
3.14
2.34
2.12
1.30
1.46
3.16
6.19
3.00
0.97
f1 (ppm)

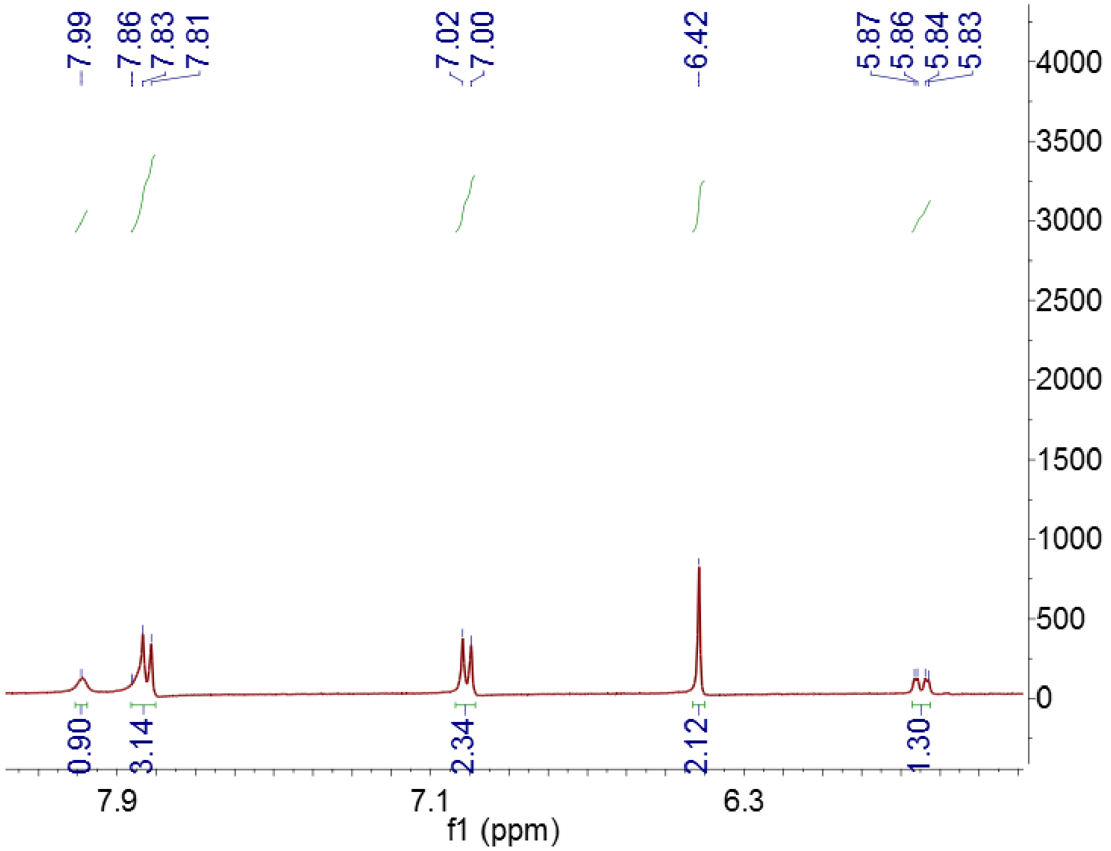
7.99
7.86
7.83
7.81
7.02
7.00
6.42
5.87
5.86
5.84
5.83
0.90
3.14
2.34
2.12
1.30
7.9
7.1
6.3
f1 (ppm)

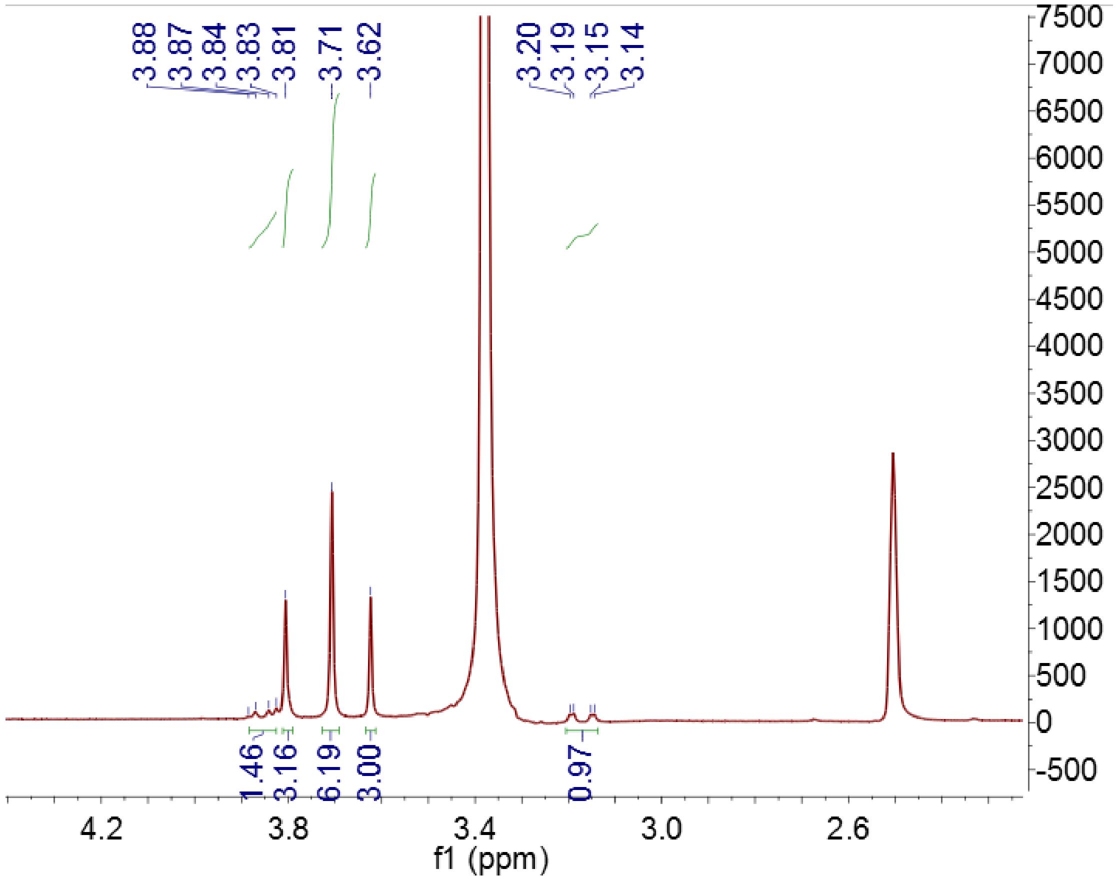
3.88
3.87
3.84
3.83
3.81
3.71
3.62
3.20
3.19
3.15
3.14
1.46
3.16
6.19
3.00
0.97
4.2
3.8
3.4
3.0
2.6
f1 (ppm)

12b

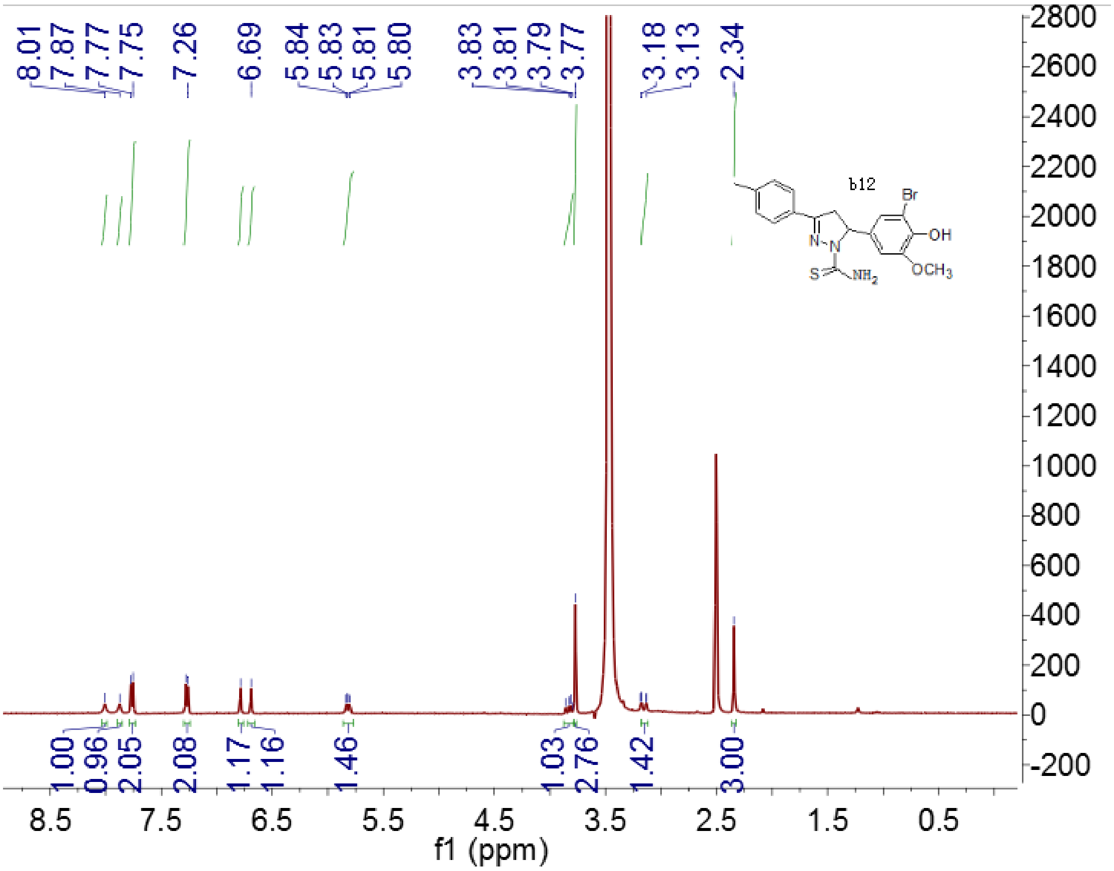
8.01 7.87 7.77 7.75 7.26 6.69 5.84 5.83 5.81 5.80 3.83 3.81 3.79 3.77 3.18 3.13 2.34
b12
Br
OH
OCH3
S
NH2
1.00 0.96 2.05 2.08 1.17 1.16 1.46 1.03 2.76 1.42 3.00
f1 (ppm)

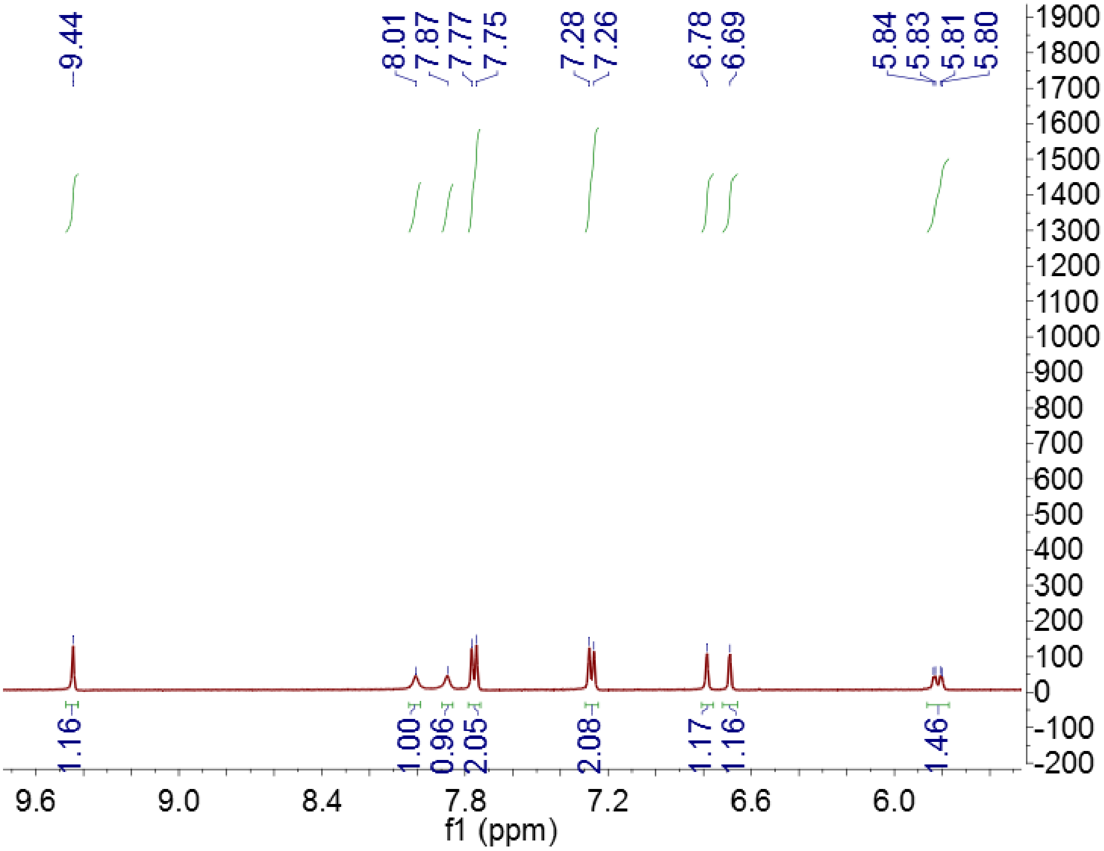
9.44 8.01 7.87 7.77 7.75 7.28 7.26 6.78 6.69 5.84 5.83 5.81 5.80
1.16 1.00 0.96 2.05 2.08 1.17 1.16 1.46
f1 (ppm)

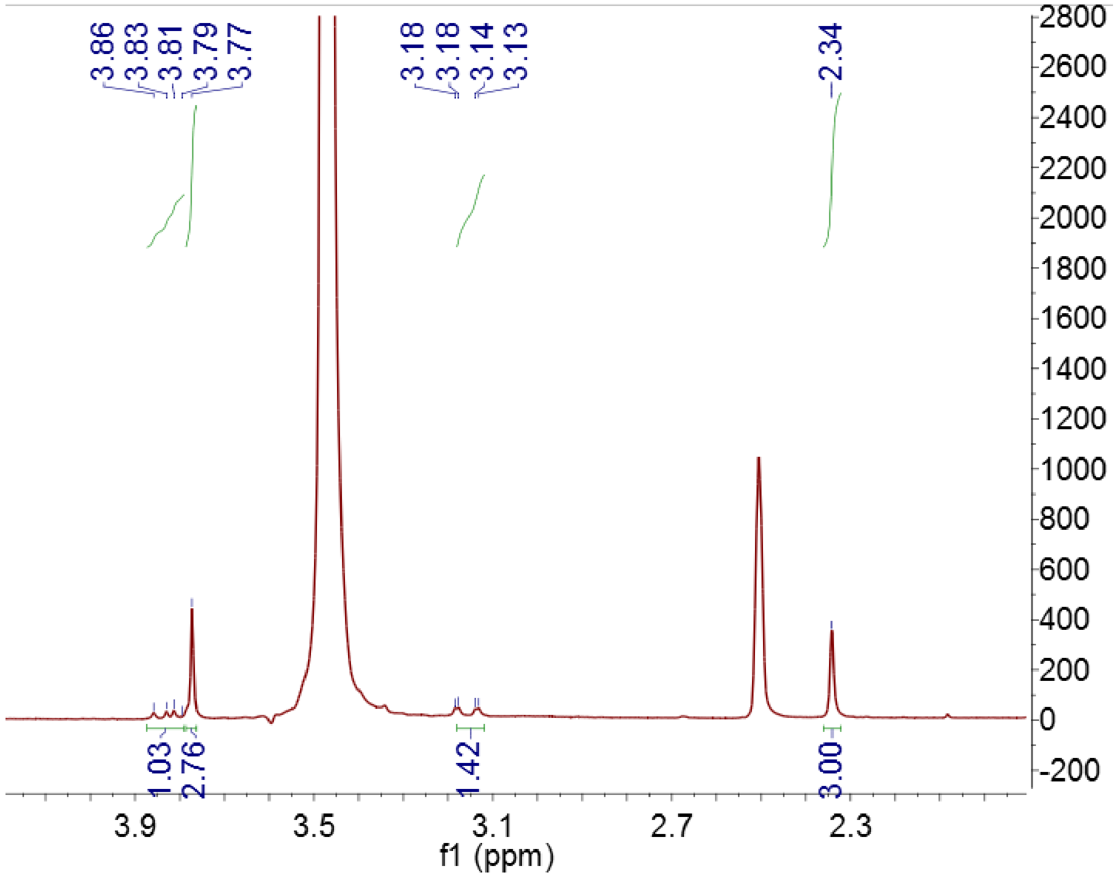

Figure S2. The representative ^{13}C NMR of compound **1b** and compound **2b**.

^{13}C NMR spectrum of compound 1b and 2b (150 MHz, DMSO)

1b

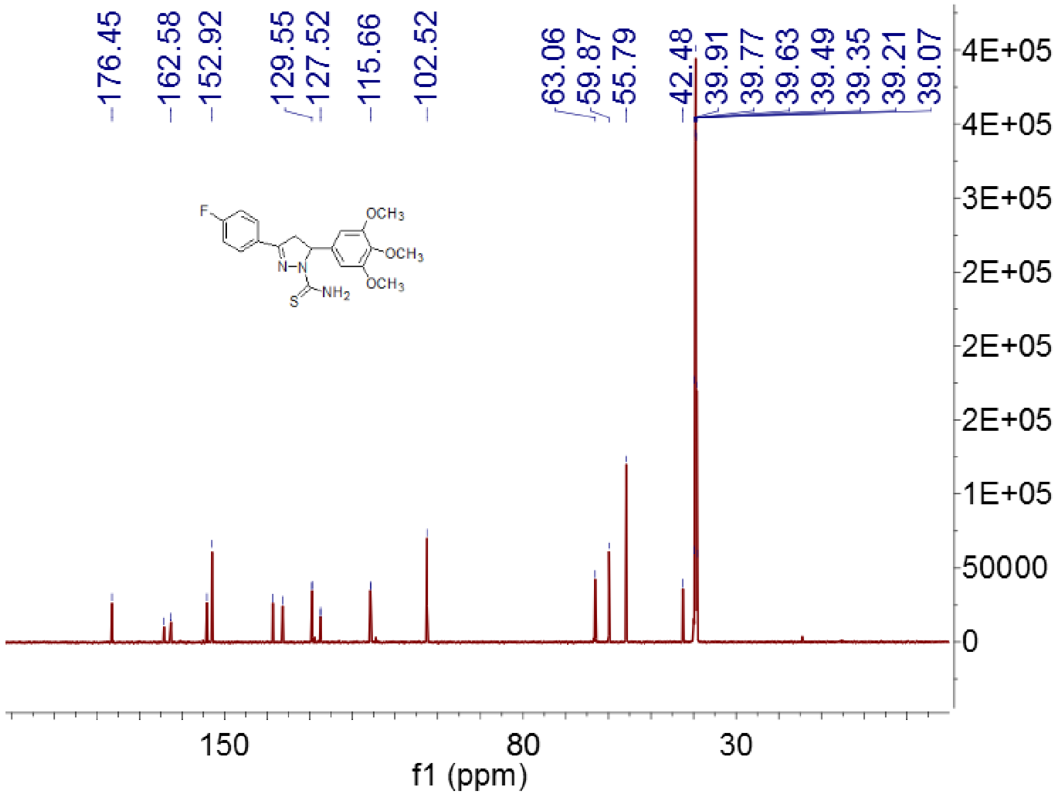

2b

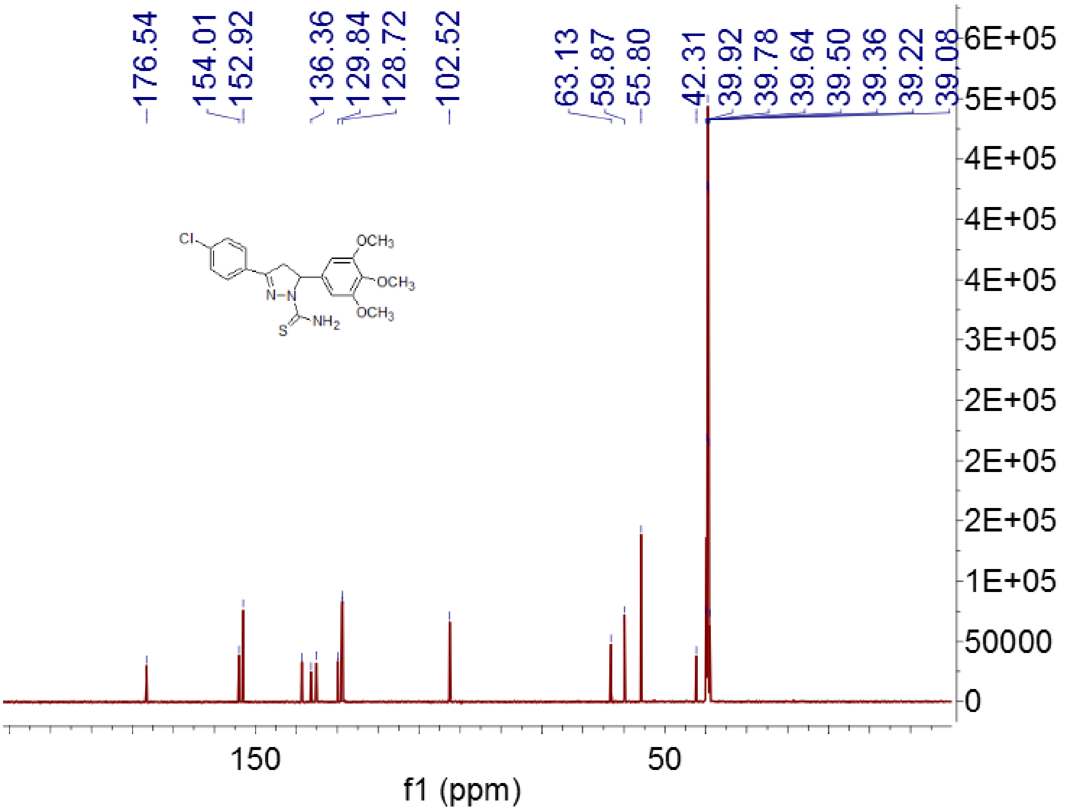

176.54
154.01
152.92
136.36
129.84
128.72
102.52
63.13
59.87
55.80
42.31
39.92
39.78
39.64
39.50
39.36
39.22
39.08
Cl
OCH3
OCH3
OCH3
N-N
S
NH2
6E+05
5E+05
4E+05
4E+05
4E+05
3E+05
2E+05
2E+05
2E+05
1E+05
50000
0
150
50
f1 (ppm)

3b

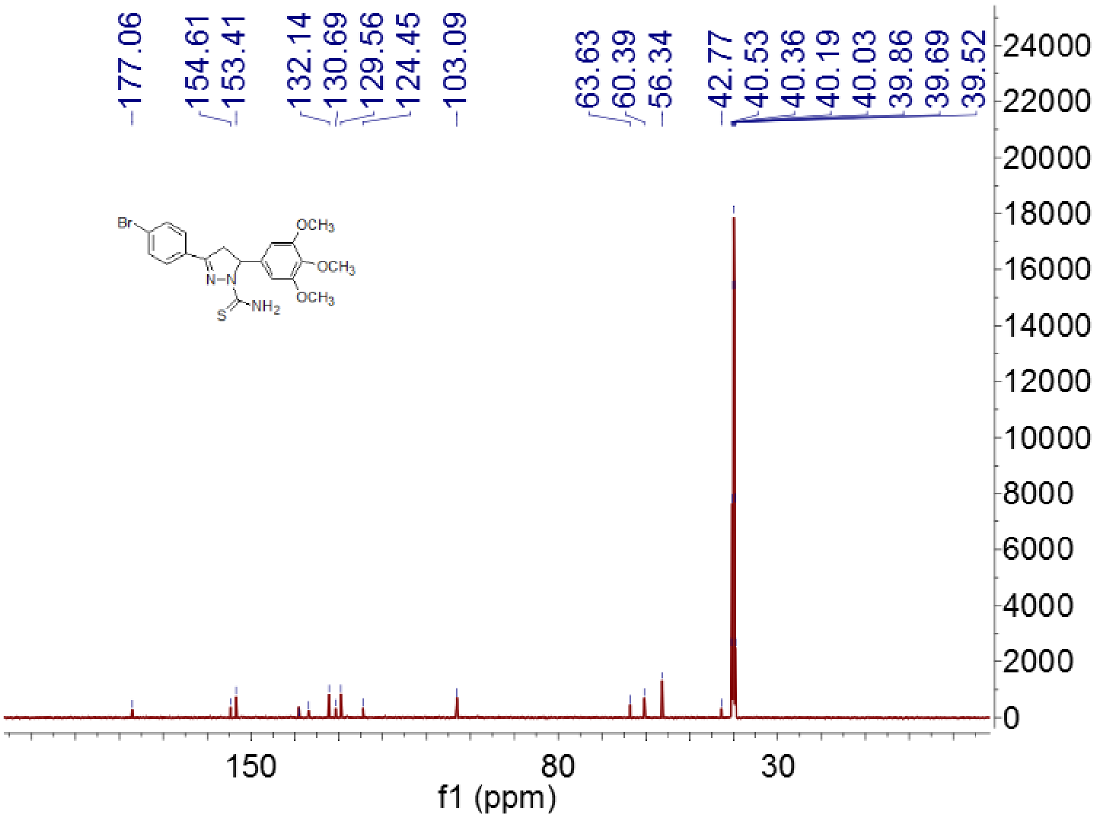

177.06
154.61
153.41
132.14
130.69
129.56
124.45
103.09
63.63
60.39
56.34
42.77
40.53
40.36
40.19
40.03
39.86
39.69
39.52
Br
OCH3
OCH3
OCH3
N-N
S
NH2
24000
22000
20000
18000
16000
14000
12000
10000
8000
6000
4000
2000
0
150
80
30
f1 (ppm)

4b

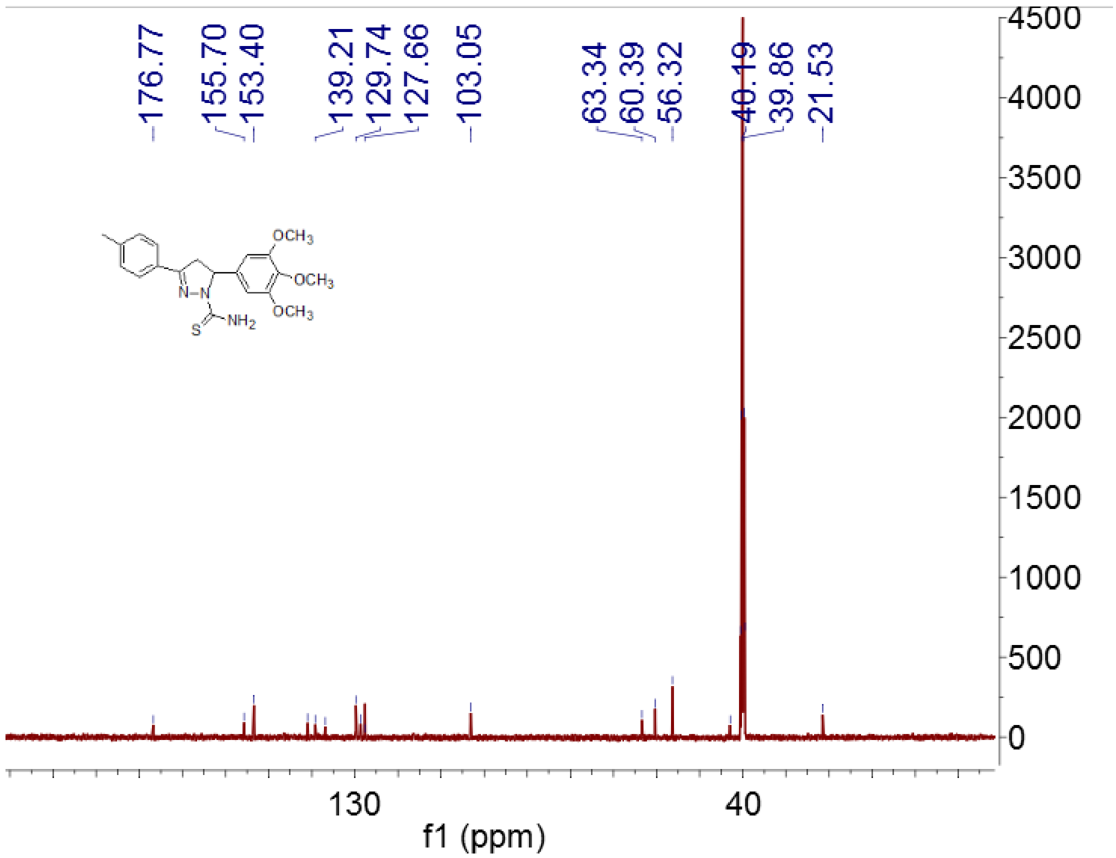
176.77
155.70
153.40
139.21
129.74
127.66
103.05
63.34
60.39
56.32
40.19
39.86
21.53
OCH3
OCH3
OCH3
N–N
NH2
S
4500
4000
3500
3000
2500
2000
1500
1000
500
0
130
40
f1 (ppm)

5b

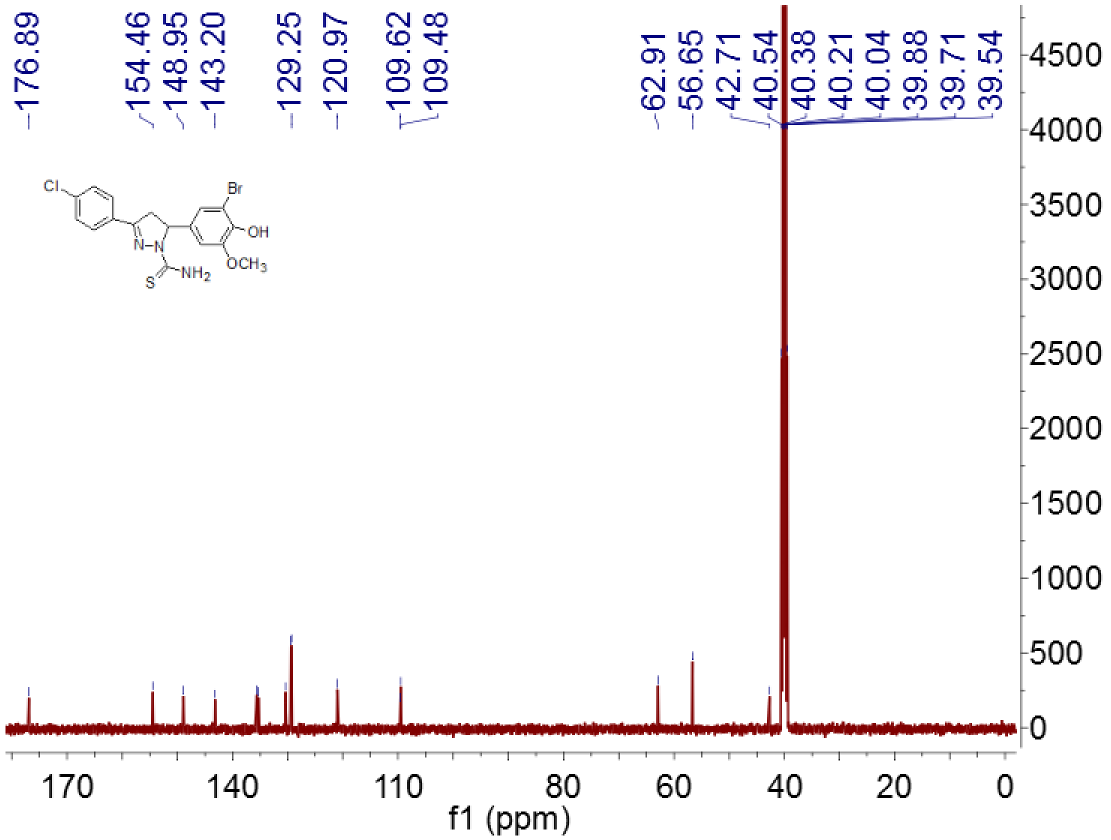
176.89
154.46
148.95
143.20
129.25
120.97
109.62
109.48
62.91
56.65
42.71
40.54
40.38
40.21
40.04
39.88
39.71
39.54
Cl
Br
OH
OCH3
N–N
NH2
S
4500
4000
3500
3000
2500
2000
1500
1000
500
0
170
140
110
80
60
40
20
0
f1 (ppm)

7b

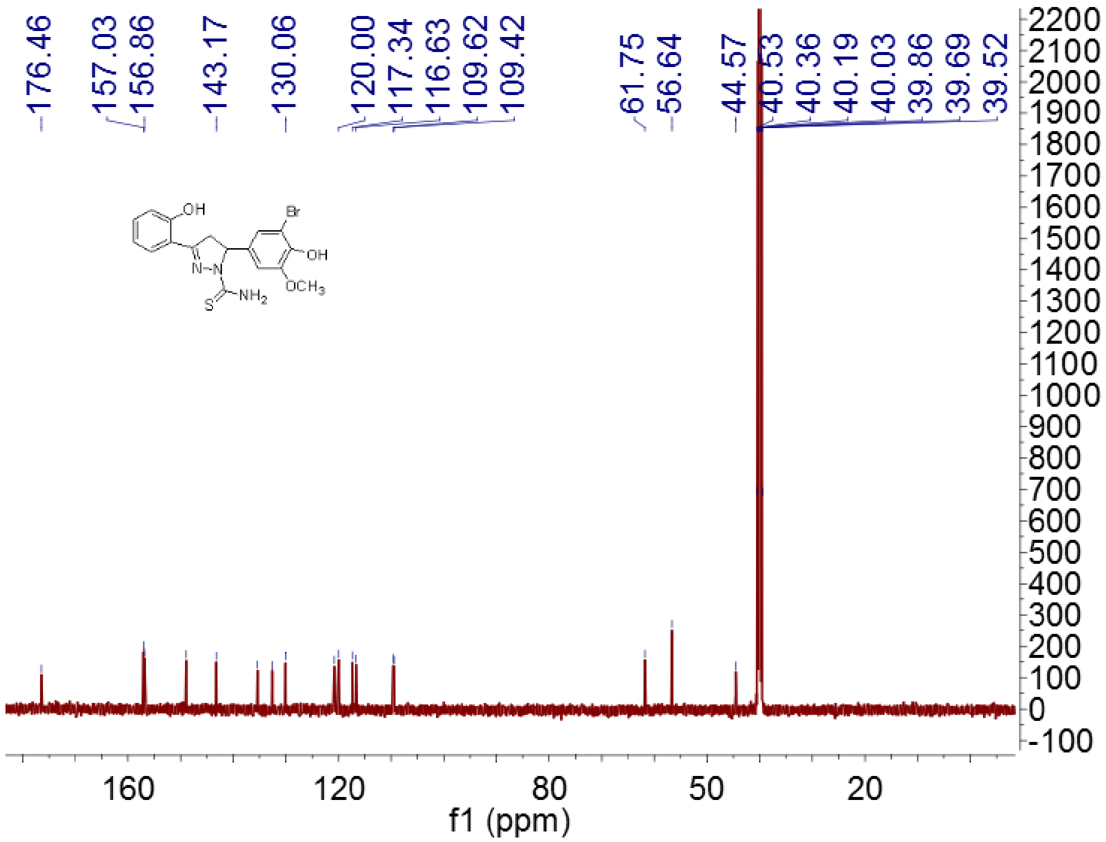
176.46
157.03
156.86
143.17
130.06
120.00
117.34
116.63
109.62
109.42
61.75
56.64
44.57
40.53
40.36
40.19
40.03
39.86
39.69
39.52
OH
Br
OH
N-N
OCH3
S
NH2
2200
2100
2000
1900
1800
1700
1600
1500
1400
1300
1200
1100
1000
900
800
700
600
500
400
300
200
100
0
-100
160
120
80
50
20
f1 (ppm)

8b

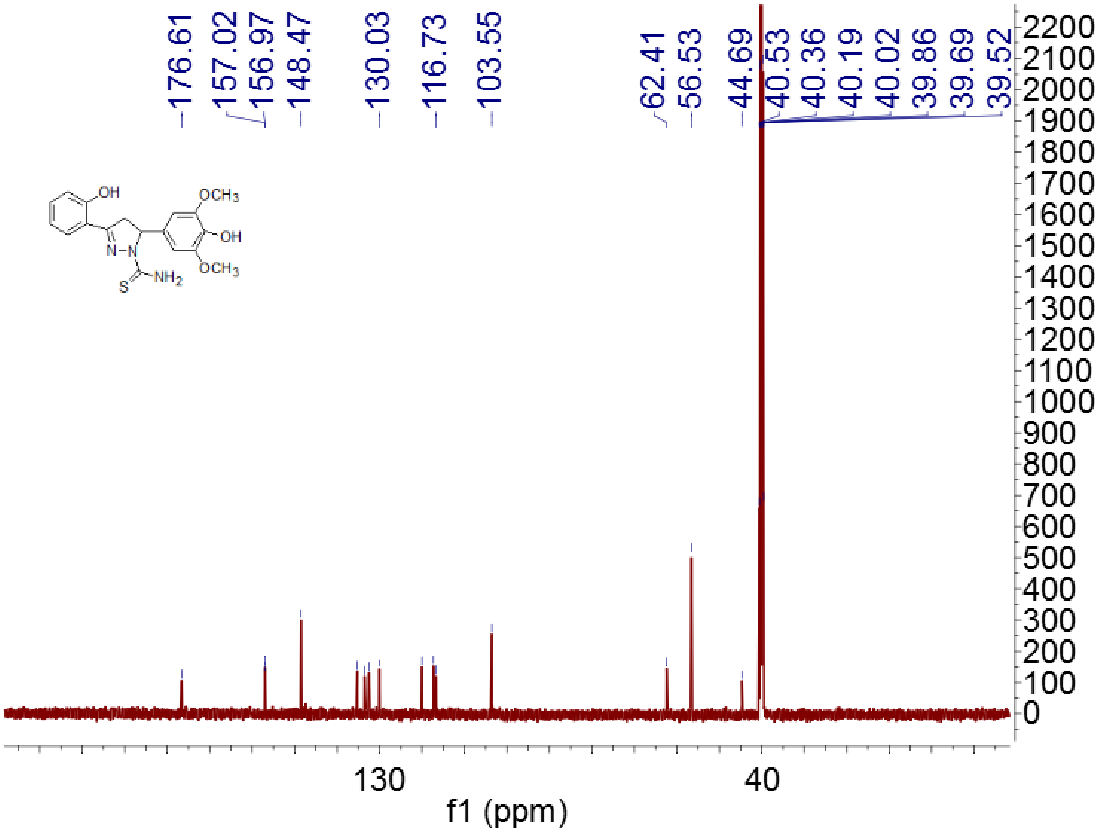
176.61
157.02
156.97
148.47
130.03
116.73
103.55
62.41
56.53
44.69
40.53
40.36
40.19
40.02
39.86
39.69
39.52
OH
OCH3
OH
N-N
OCH3
S
NH2
2200
2100
2000
1900
1800
1700
1600
1500
1400
1300
1200
1100
1000
900
800
700
600
500
400
300
200
100
0
130
40
f1 (ppm)

9b

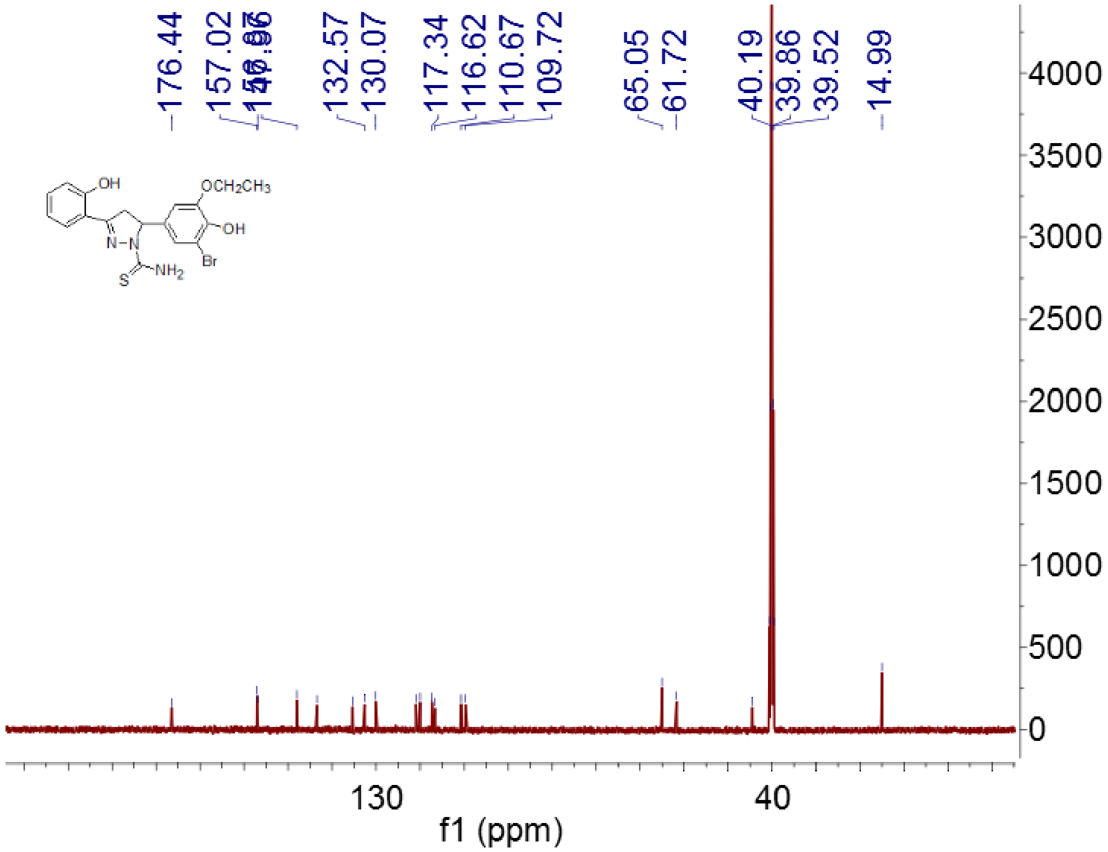
176.44
157.02
132.57
130.07
117.34
116.62
110.67
109.72
65.05
61.72
40.19
39.86
39.52
14.99
OH
OCH2CH3
OH
Br
N–N
S
NH2
4000
3500
3000
2500
2000
1500
1000
500
0
130
40
f1 (ppm)

10b

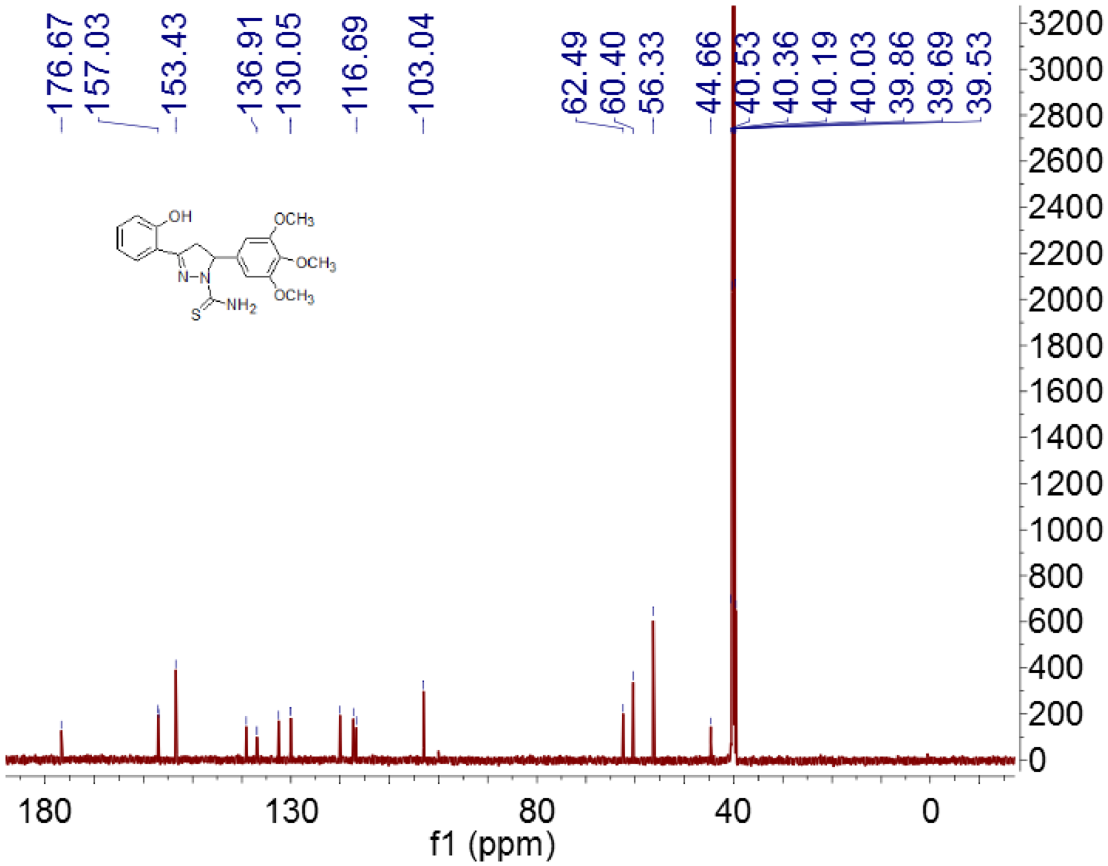
176.67
157.03
153.43
136.91
130.05
116.69
103.04
62.49
60.40
56.33
44.66
40.53
40.36
40.19
40.03
39.86
39.69
39.53
OH
OCH3
OCH3
OCH3
N–N
S
NH2
3200
3000
2800
2600
2400
2200
2000
1800
1600
1400
1200
1000
800
600
400
200
0
180
130
80
40
0
f1 (ppm)

11b

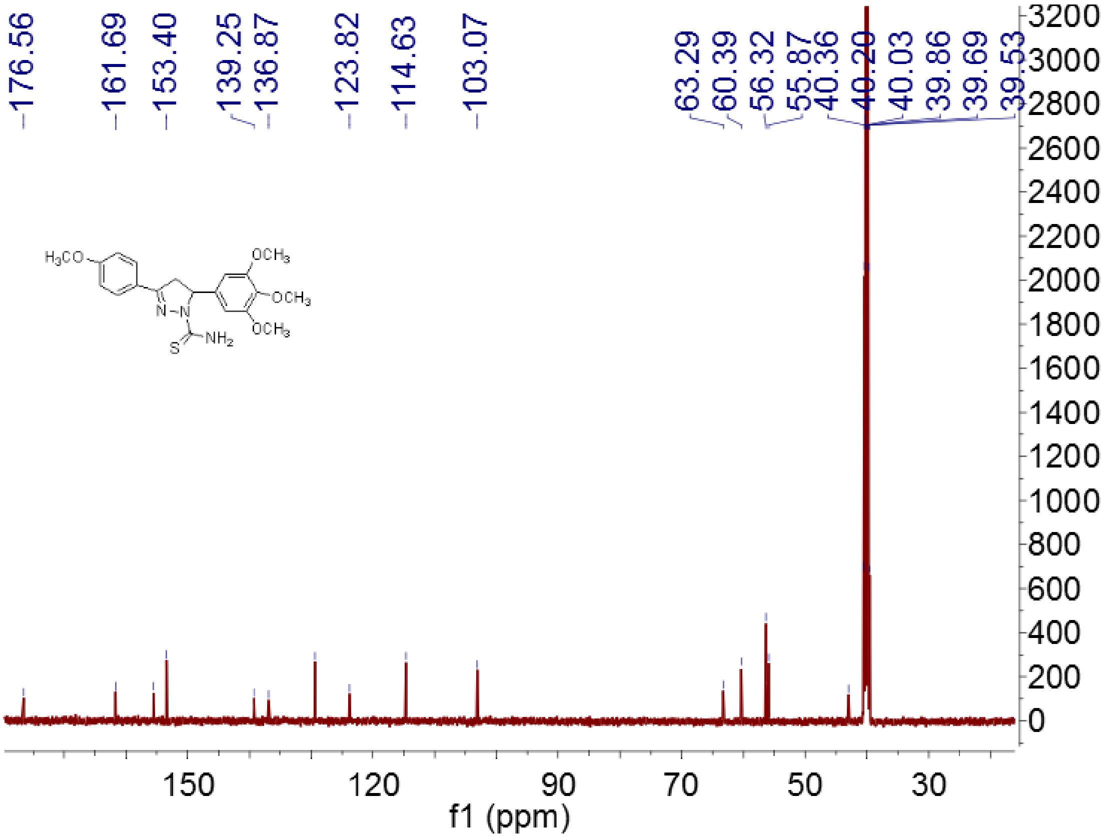

12b

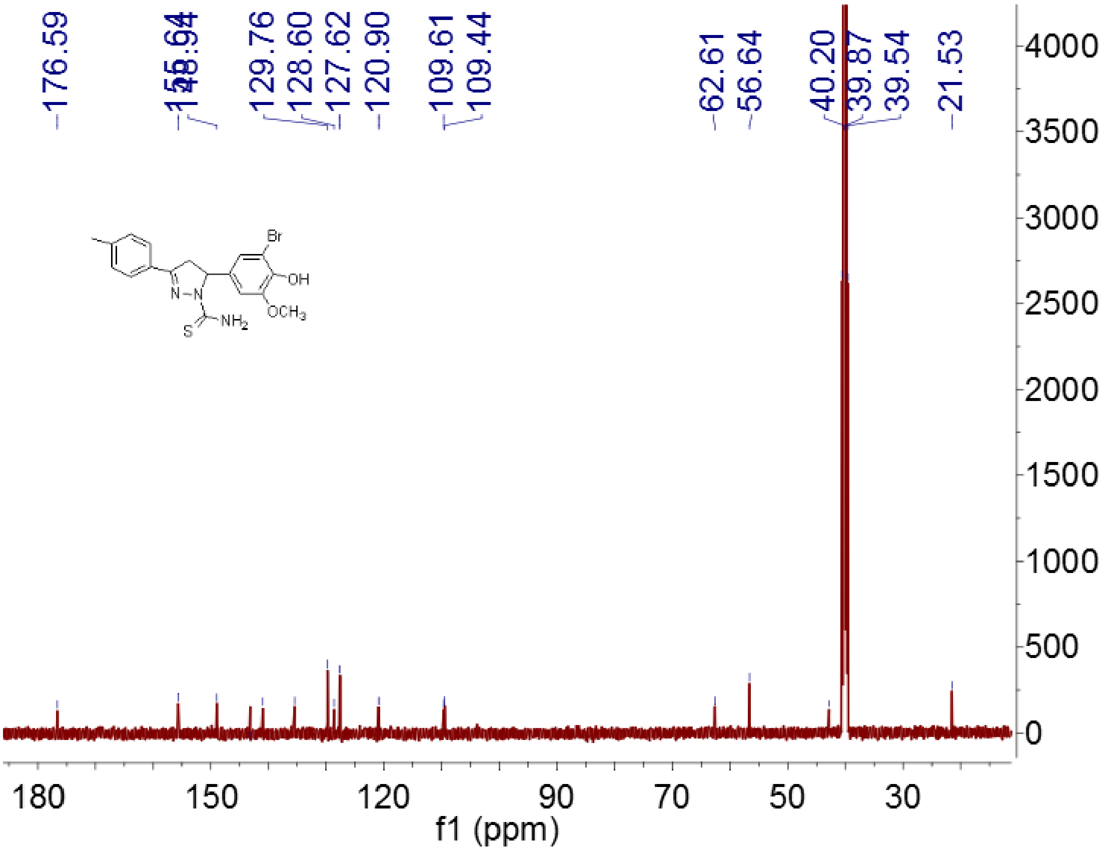

Figure S3. The representative HRMS (ESI) spectra of compound **1b-12b**.

HRMS (ESI) spectrum of compound 1b

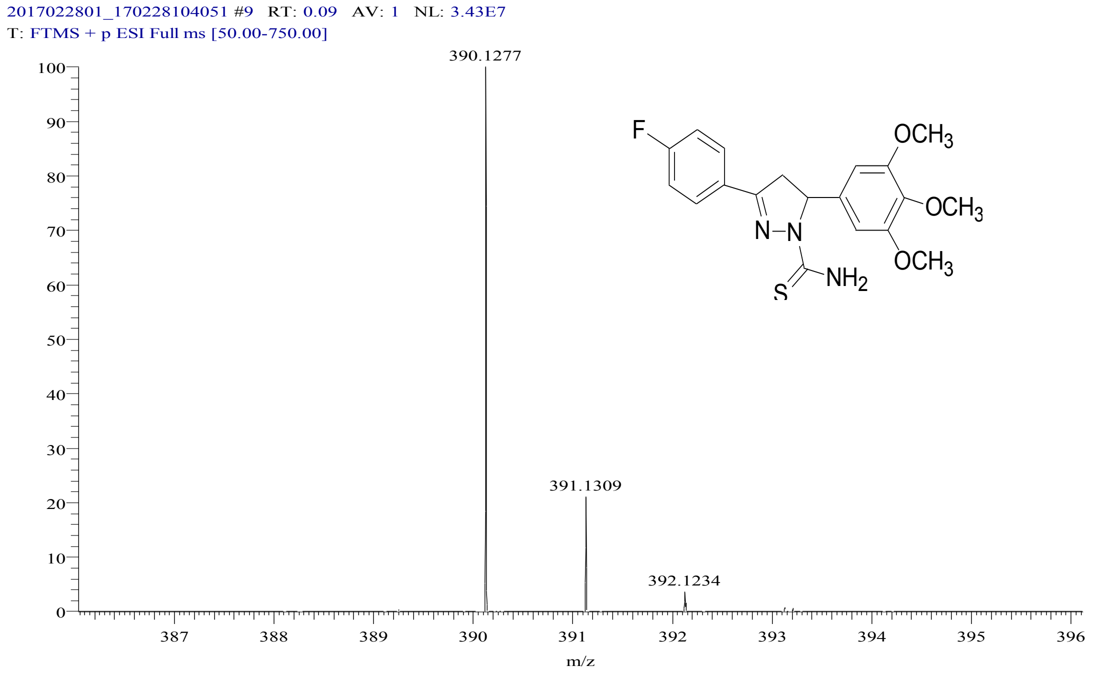

Elemental Composition calculator

Target M/Z	390.1277	Result type:	Positive	Species:	$[M+H]^+$
Elements:		C(0-40); H(0-80); O(0-10); N(0-10); F(0-1); S(0-1)			
Ion Formula		Calculated M/Z		PPM error	
$C_{19}H_{21}N_3O_3FS$		390.1282		-1.28	

HRMS (ESI) spectrum of compound 2b

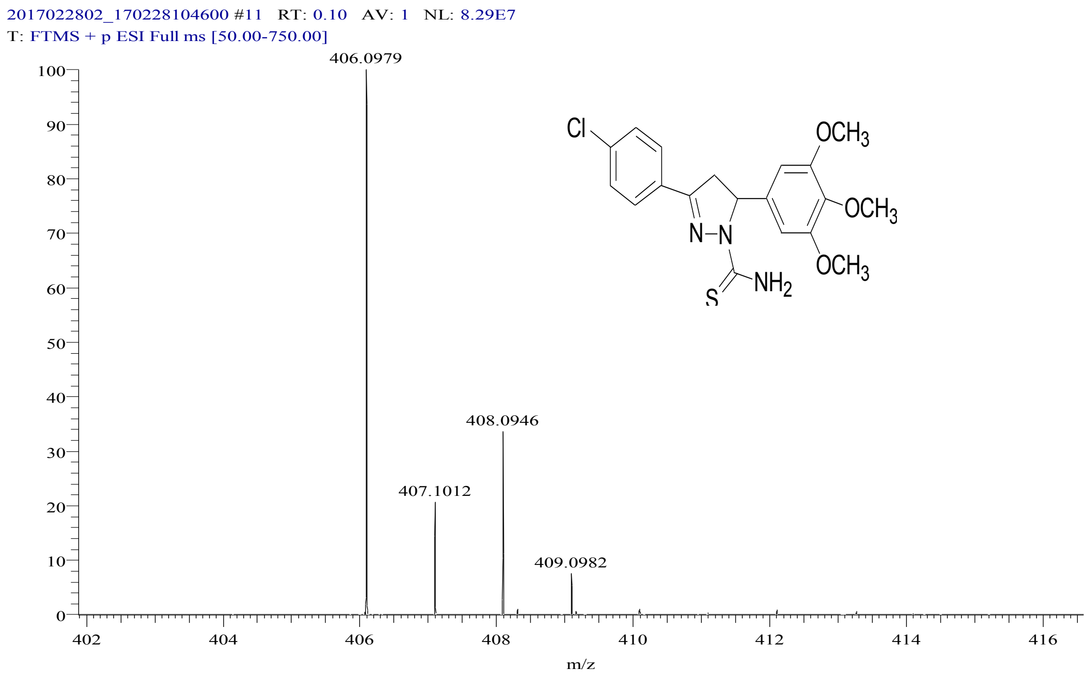

Elemental Composition calculator

Target M/Z	406.0979	Result type:	Positive	Species：	$[M+H]^+$
Elements:		C(0-40); H(0-80); O(0-10); N(0-10); Cl(0-1); S(0-1)			
Ion Formula		Calculated M/Z		PPM error	
$C_{19}H_{21}N_3O_3SCl$		406.0987		-1.97	

HRMS (ESI) spectrum of compound 3b

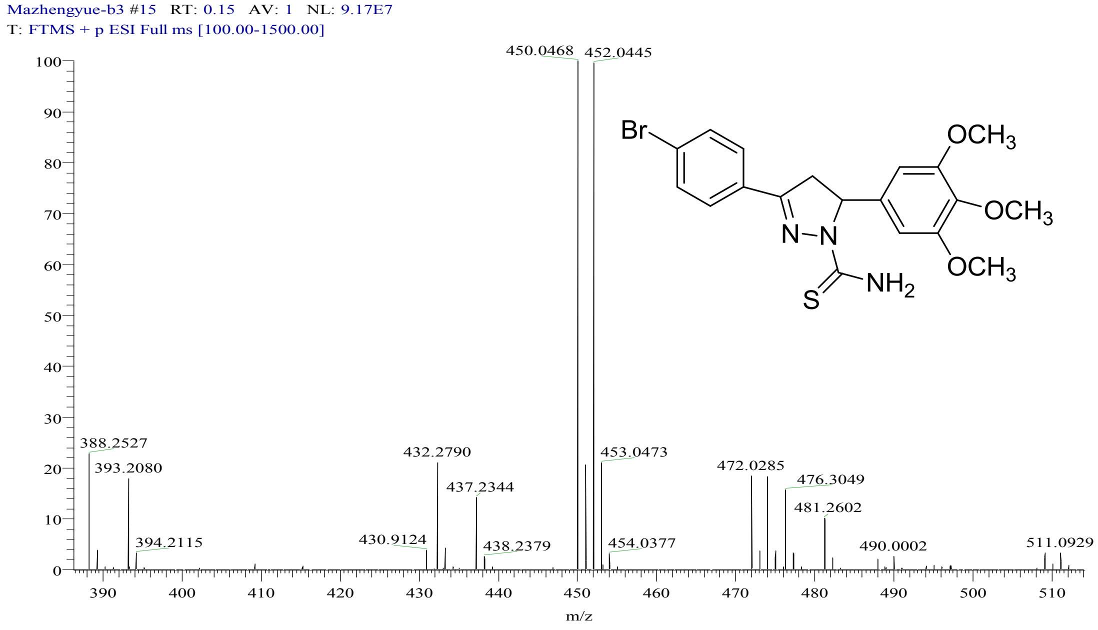

Elemental Composition calculator

Target M/Z	450.0468	Result type:	Positive	Species:	$[M+H]^+$
Elements:		C(0-40); H(0-80); O(0-10); N(0-10); Br(0-1); S(0-1)			
Ion Formula		Calculated M/Z		PPM error	
$C_{19}H_{20}BrN_3O_3S$		450.0487		-4.22	

HRMS (ESI) spectrum of compound 4b

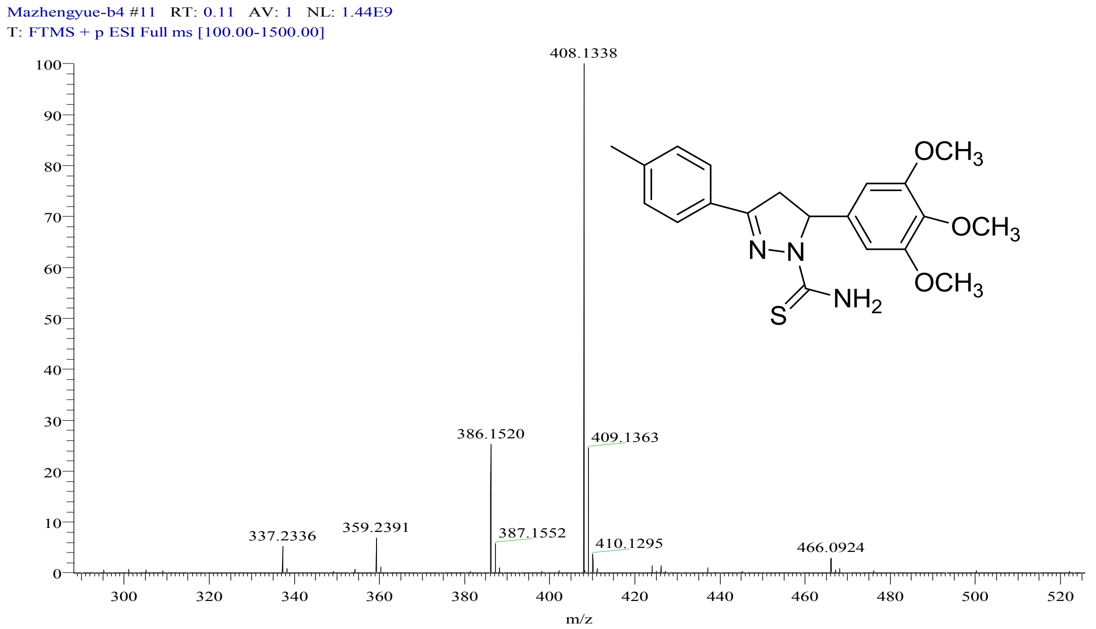

Elemental Composition calculator

Target M/Z	408.1338	Result type:	Positive	Species:	$[M+Na]^+$
Elements:		C(0-40); H(0-80); O(0-10); N(0-10); S(0-1)			
Ion Formula		Calculated M/Z		PPM error	
$C_{20}H_{23}N_3O_3S$		408.1358		-4.9	

HRMS (ESI) spectrum of compound 5b

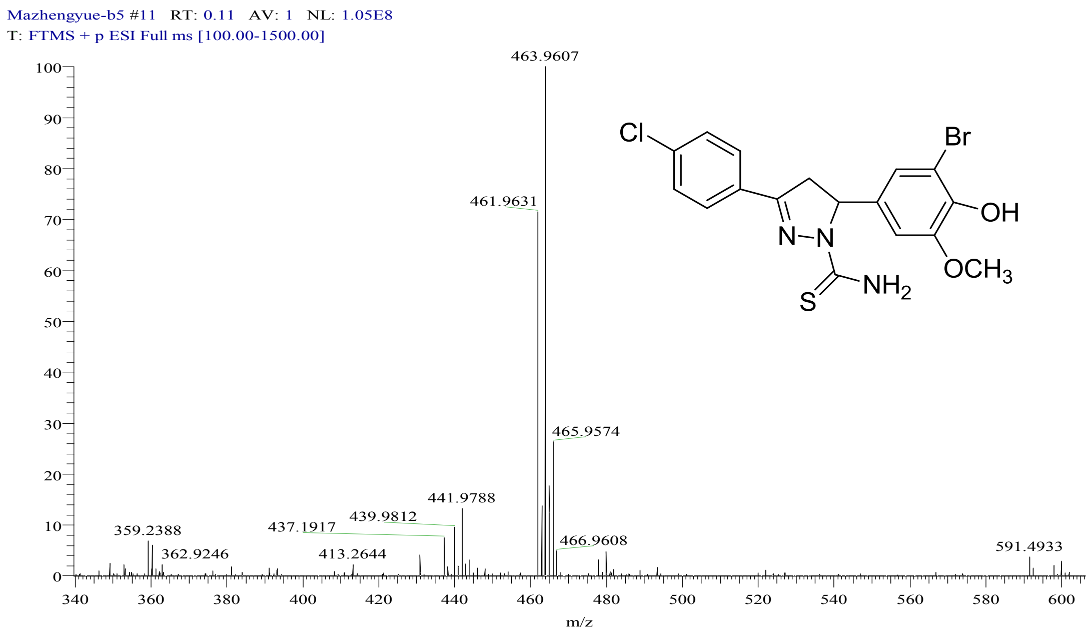

Elemental Composition calculator

Target M/Z	461.9631	Result type:	Positive	Species：	$[M+Na]^+$
Elements:		C(0-40); H(0-80); O(0-10); N(0-10); Br(0-1); Cl(0-1); S(0-1)			
Ion Formula		Calculated M/Z		PPM error	
$C_{17}H_{15}BrClN_3O_2S$		461.9634		-0.6	

HRMS (ESI) spectrum of compound 6b

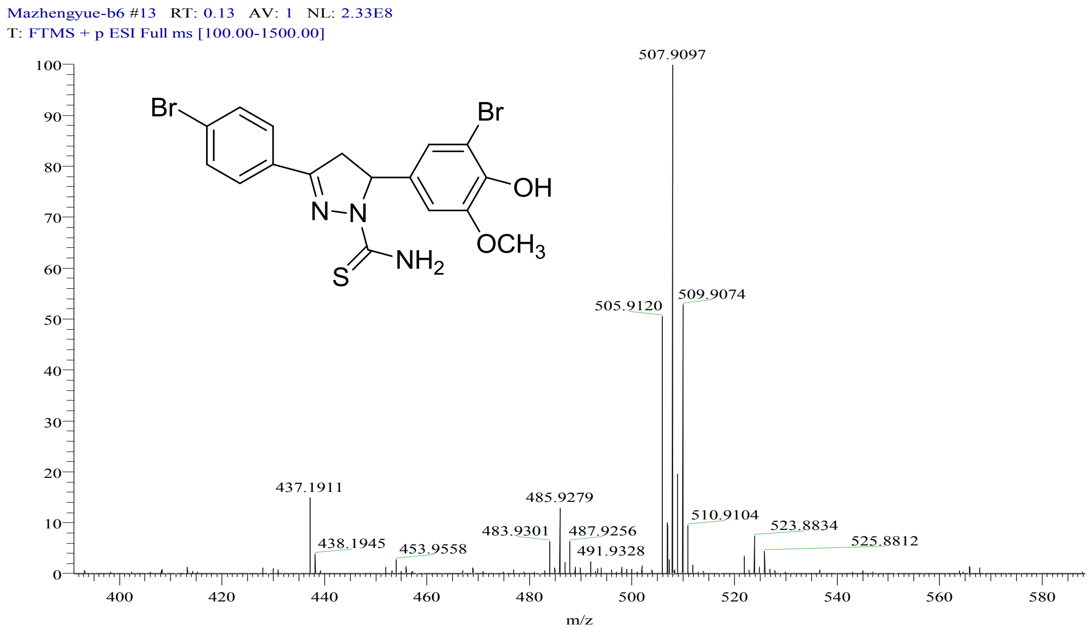

Elemental Composition calculator

Target M/Z	507.9097	Result type:	Positive	Species:	$[M+Na]^+$
Elements:		C(0-40); H(0-80); O(0-10); N(0-10); Br(0-1); S(0-1)			
Ion Formula		Calculated M/Z		PPM error	
$C_{17}H_{15}Br_2N_3O_2S$		507.9107		-1.96	

HRMS (ESI) spectrum of compound 7b

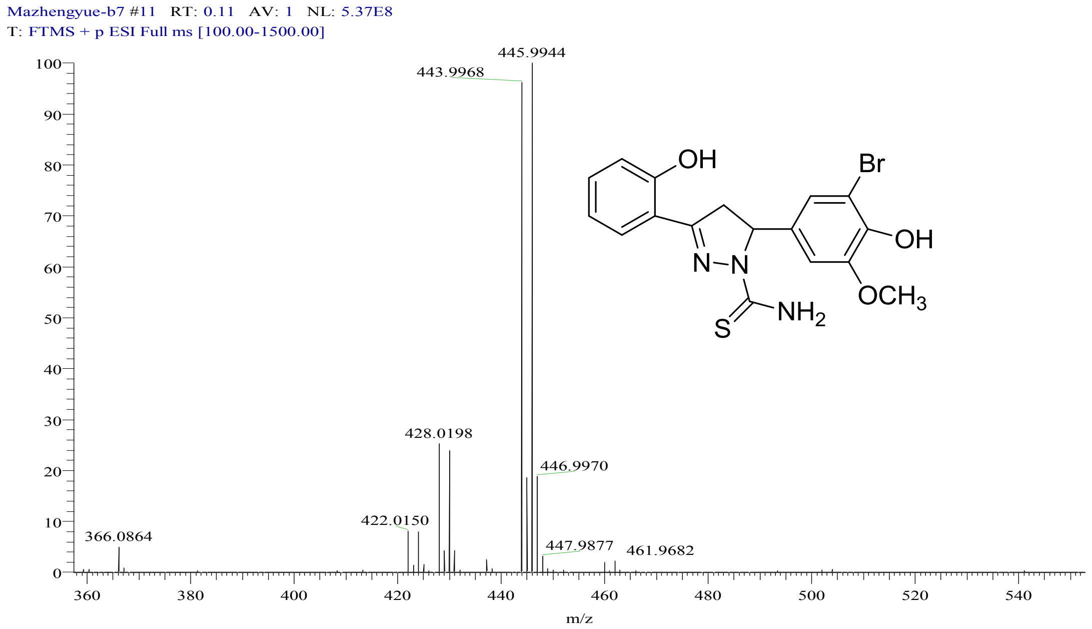

Elemental Composition calculator

Target M/Z	443.9968	Result type:	Positive	Species:	$[M+Na]^+$
Elements:		C(0-40); H(0-80); O(0-10); N(0-10); Br(0-1); S(0-1)			
Ion Formula		Calculated M/Z		PPM error	
$C_{17}H_{16}BrN_3O_3S$		443.9973		-1.1	

HRMS (ESI) spectrum of compound 8b

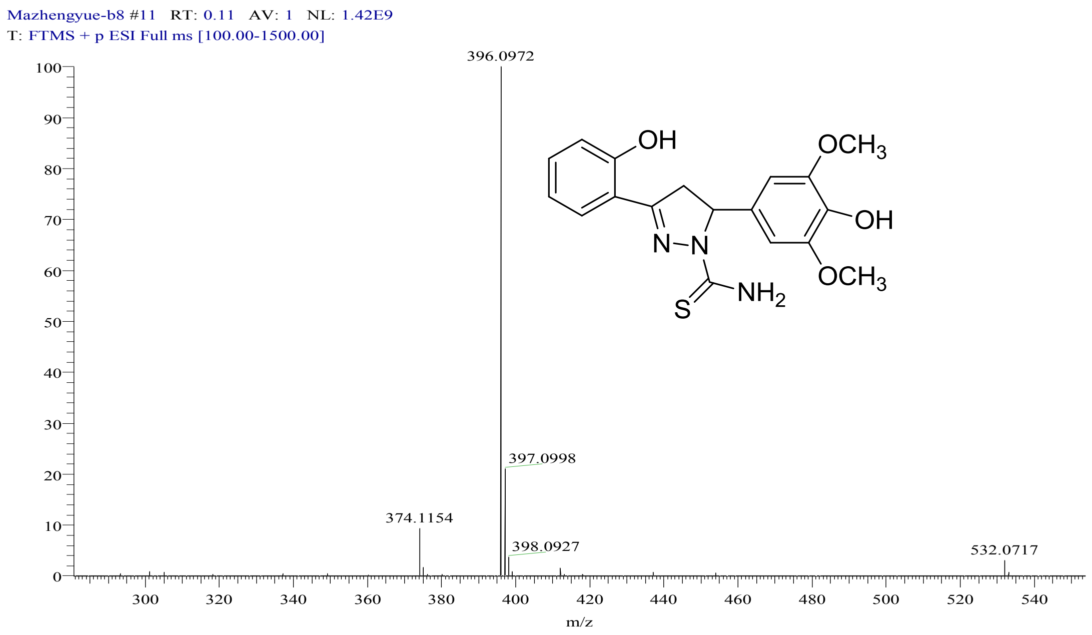

Elemental Composition calculator

Target M/Z	396.0972	Result type:	Positive	Species：	$[M+Na]^+$
Elements:		C(0-40); H(0-80); O(0-10); N(0-10); S(0-1)			
Ion Formula		Calculated M/Z		PPM error	
$C_{18}H_{19}N_3O_4S$		396.0984		-3.0	

HRMS (ESI) spectrum of compound 9b

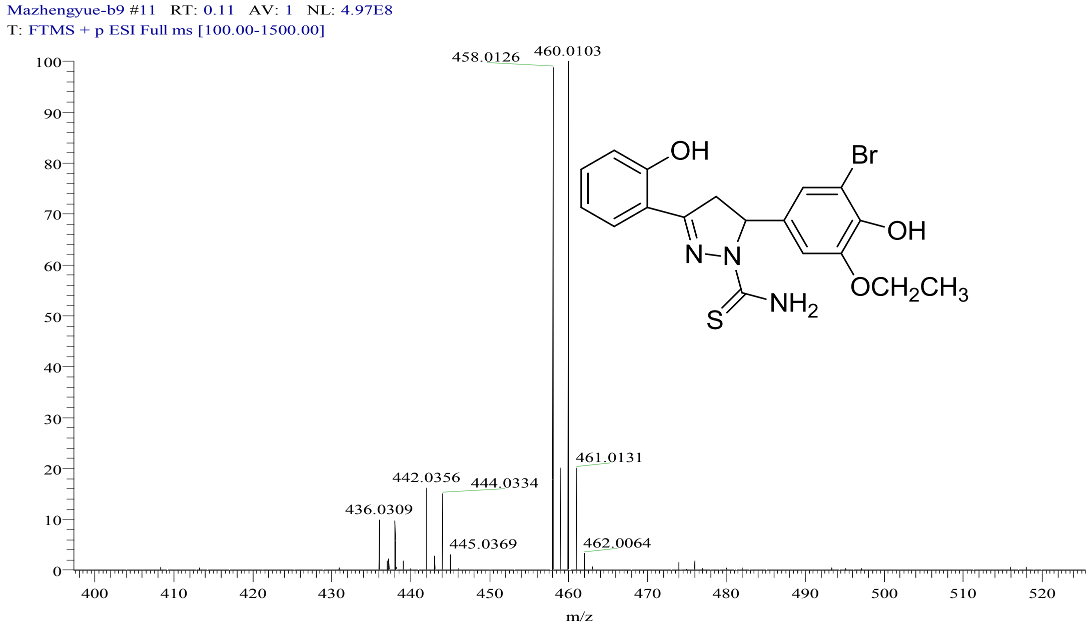

Elemental Composition calculator

Target M/Z	460.0108	Result type:	Positive	Species:	$[M+Na]^+$
Elements:		C(0-40); H(0-80); O(0-10); N(0-10); Br(0-1); S(0-1)			
Ion Formula		Calculated M/Z		PPM error	
$C_{18}H_{18}BrN_3O_3S$		460.0129		-4.5	

HRMS (ESI) spectrum of compound 10b

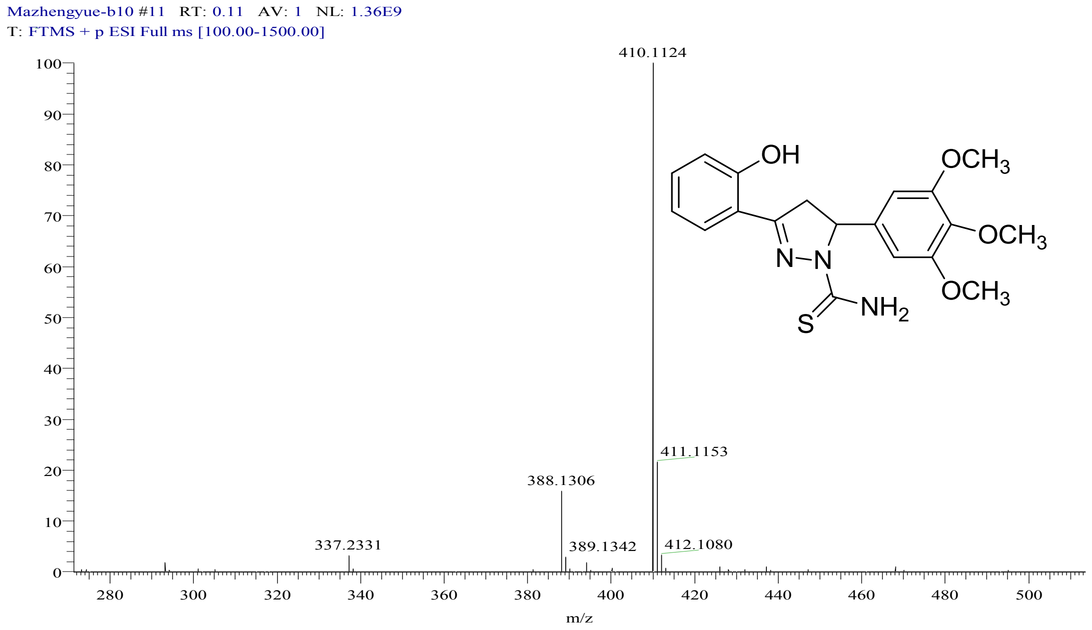

Elemental Composition calculator

Target M/Z	410.1124	Result type:	Positive	Species:	$[M+Na]^+$
Elements:		C(0-40); H(0-80); O(0-10); N(0-10); S(0-1)			
Ion Formula		Calculated M/Z		PPM error	
$C_{19}H_{21}N_3O_4S$		410.1140		-3.9	

HRMS (ESI) spectrum of compound 11b

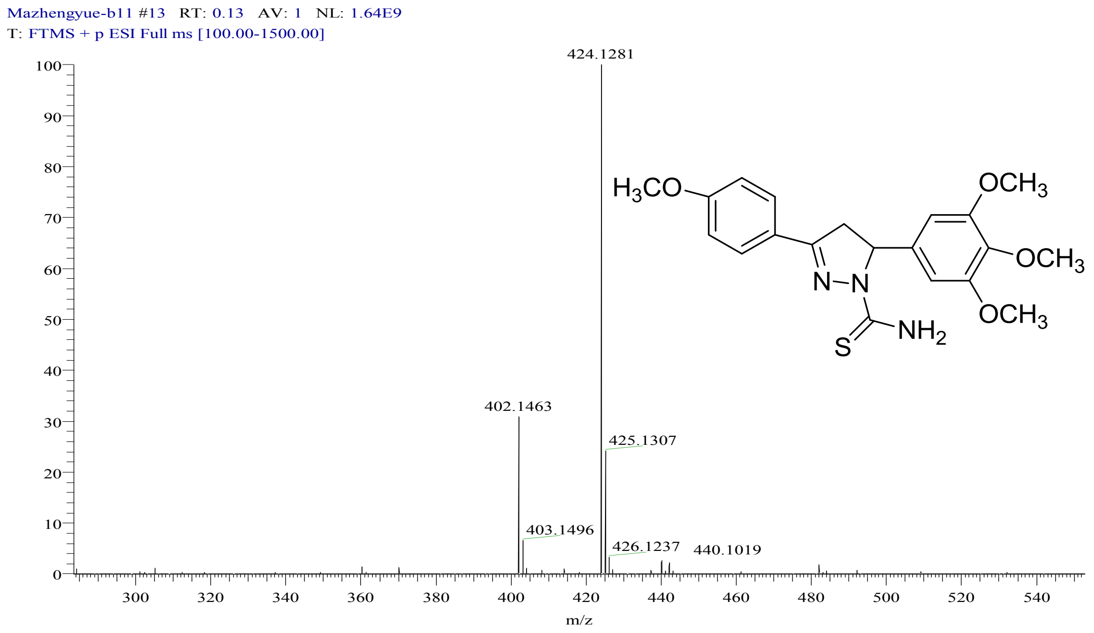

Elemental Composition calculator

Target M/Z	424.1281	Result type:	Positive	Species:	$[M+Na]^+$
Elements:		C(0-40); H(0-80); O(0-10); N(0-10); S(0-1)			
Ion Formula		Calculated M/Z		PPM error	
$C_{20}H_{23}N_3O_4S$		424.1301		-4.7	

HRMS (ESI) spectrum of compound 12b

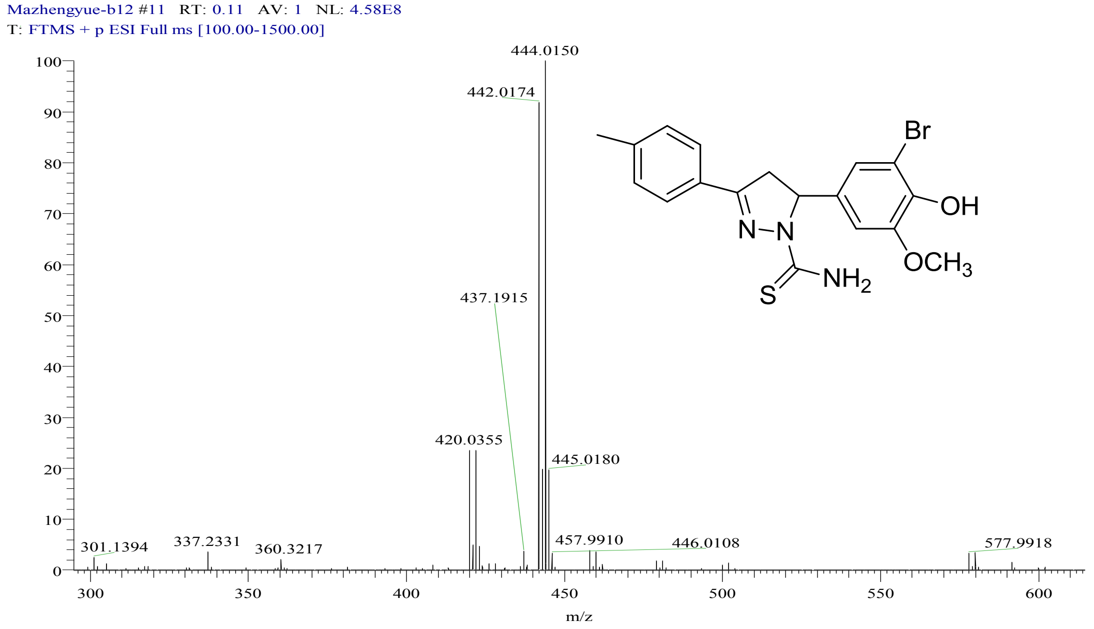

Elemental Composition calculator

Target M/Z	444.0150	Result type:	Positive	Species:	$[M+Na]^+$
Elements:		C(0-40); H(0-80); O(0-10); N(0-10); Br(0-1); S(0-1)			
Ion Formula		Calculated M/Z		PPM error	
$C_{18}H_{18}BrN_3O_2S$		444.0170		-4.5	

Figure S4. The representative IR of compound **1b-12b**.

1b

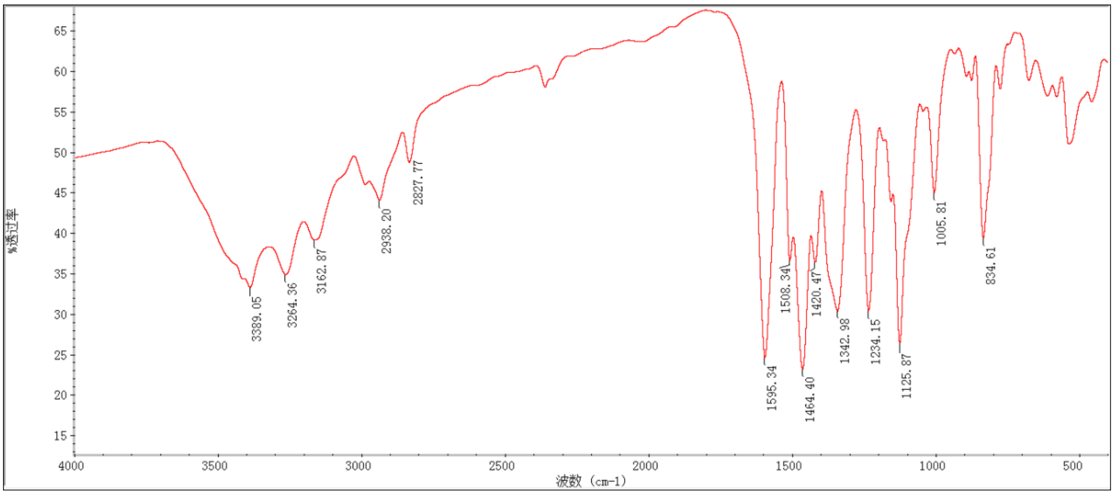

2b

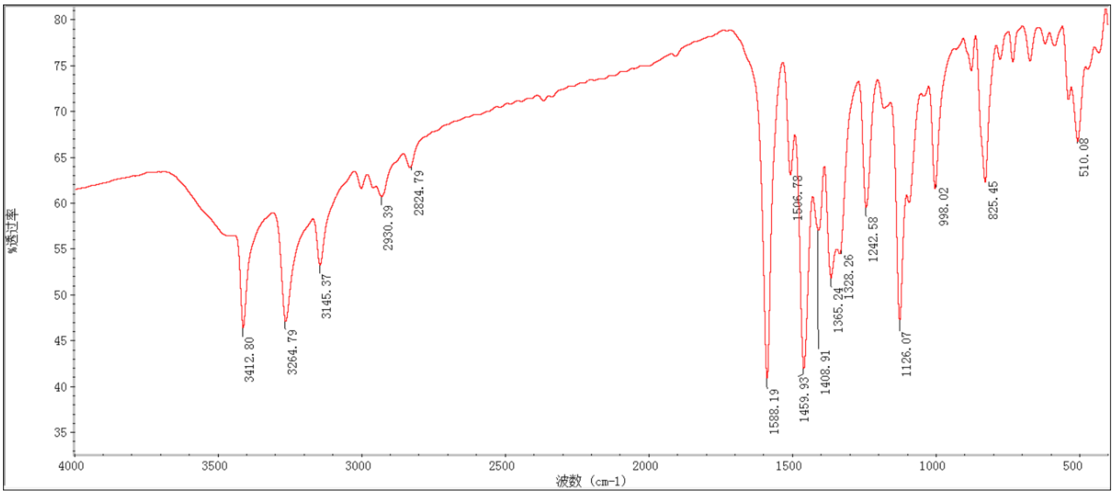

3b

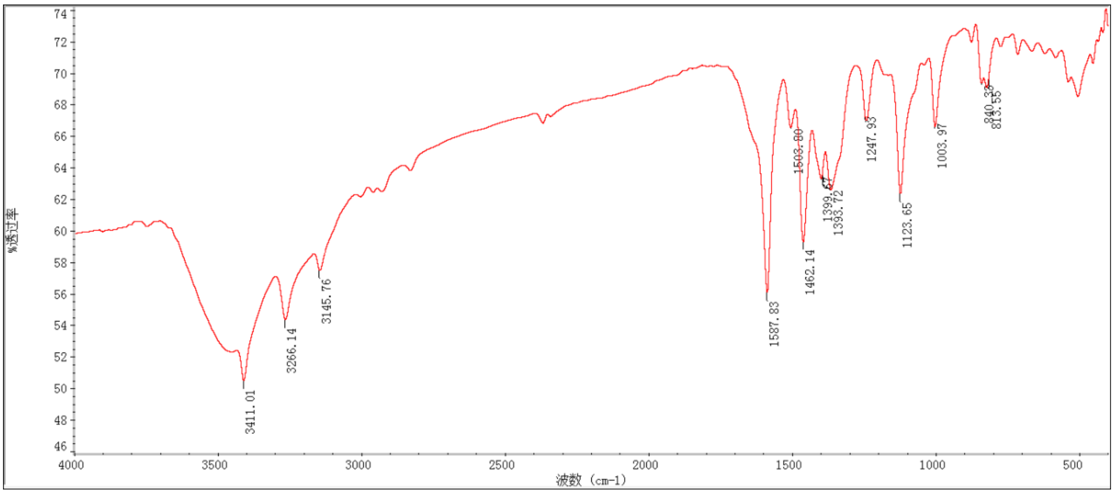
%透过率
波数 (cm-1)
3411.01
3266.14
3145.76
1587.83
1503.80
1462.14
1393.72
1247.93
1123.65
1003.97
813.55

4b

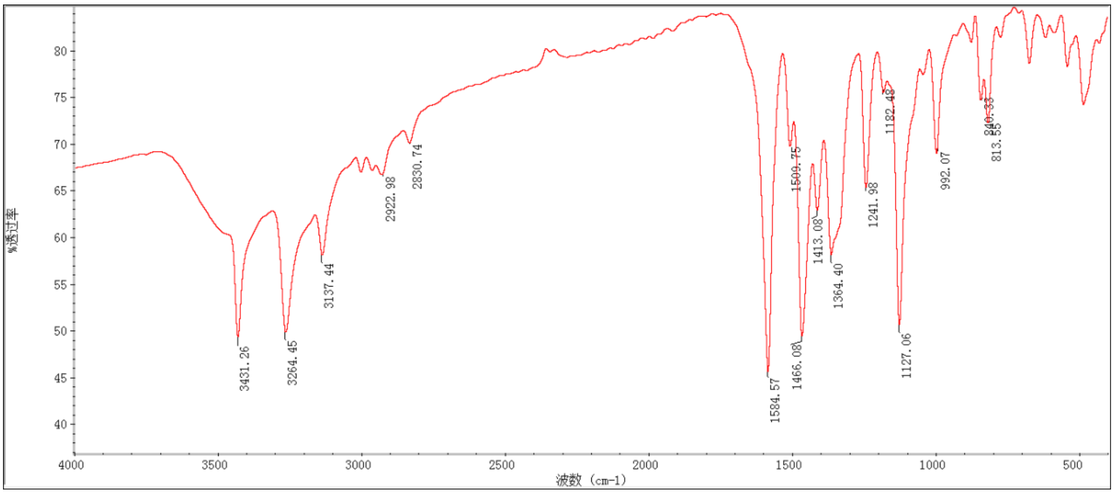
%透过率
波数 (cm-1)
3431.26
3264.45
3137.44
2922.98
2830.74
1584.57
1509.75
1466.08
1413.08
1364.40
1241.98
1182.48
1127.06
992.07
813.55

5b

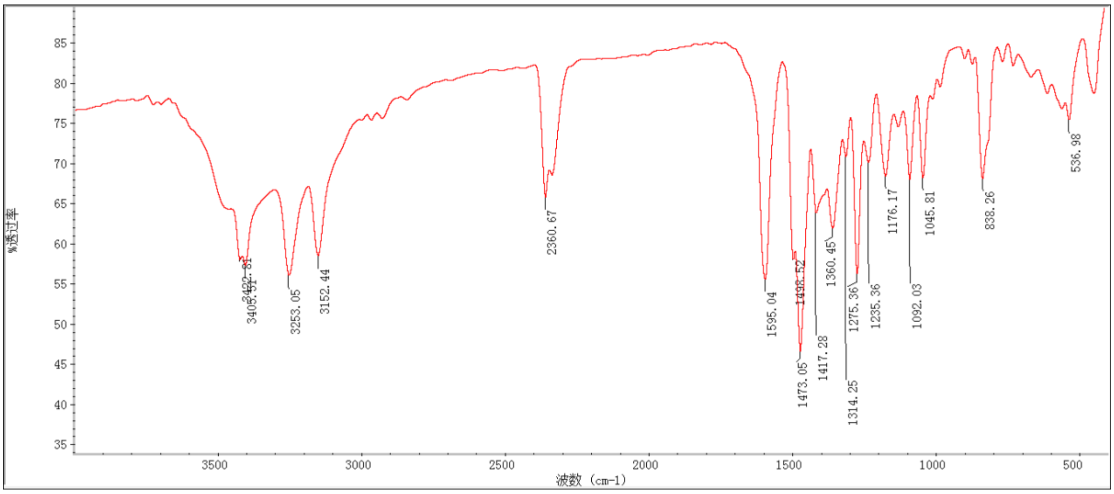
%透过率
波数 (cm-1)
3253.05
3152.44
2360.67
1595.04
1498.52
1473.05
1417.28
1360.45
1314.25
1275.36
1235.36
1176.17
1092.03
1045.81
838.26
536.98

6b

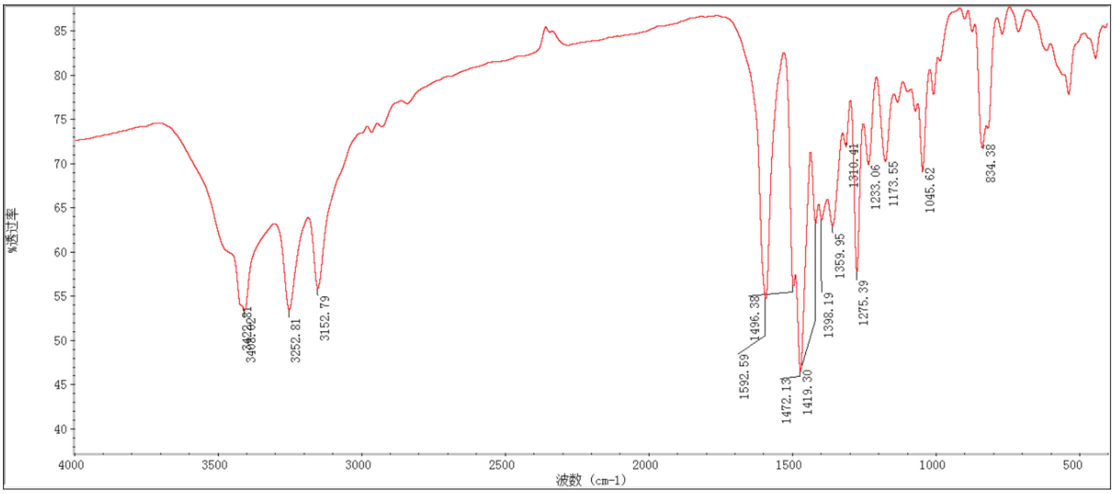
%透过率
波数（cm-1）
3252.81
3152.79
1592.59
1496.38
1472.13
1419.30
1398.19
1359.95
1310.41
1275.39
1233.06
1173.55
1045.62
834.38

7b

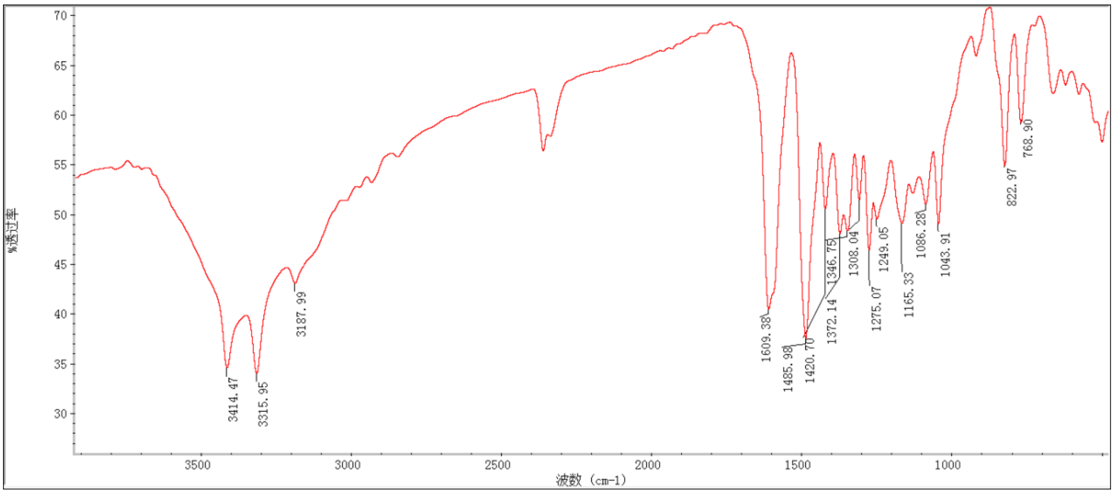
%透过率
波数（cm-1）
3414.47
3315.95
3187.99
1609.38
1485.98
1420.70
1372.14
1346.75
1308.04
1275.07
1249.05
1165.33
1086.28
1043.91
822.97
768.90

8b

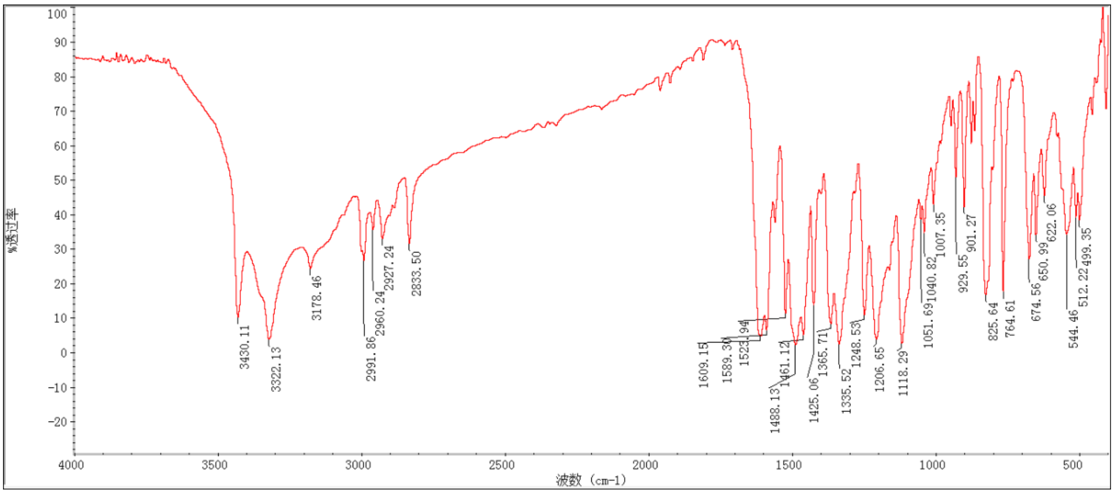
%透过率
波数（cm-1）
3430.11
3322.13
3178.46
2991.86
2960.24
2927.24
2833.50
1609.15
1589.30
1488.13
1461.12
1425.06
1365.71
1335.52
1248.53
1206.65
1118.29
1051.69
1040.82
1007.35
929.55
901.27
825.64
764.61
674.56
650.99
622.06
544.46
512.22
499.35

9b

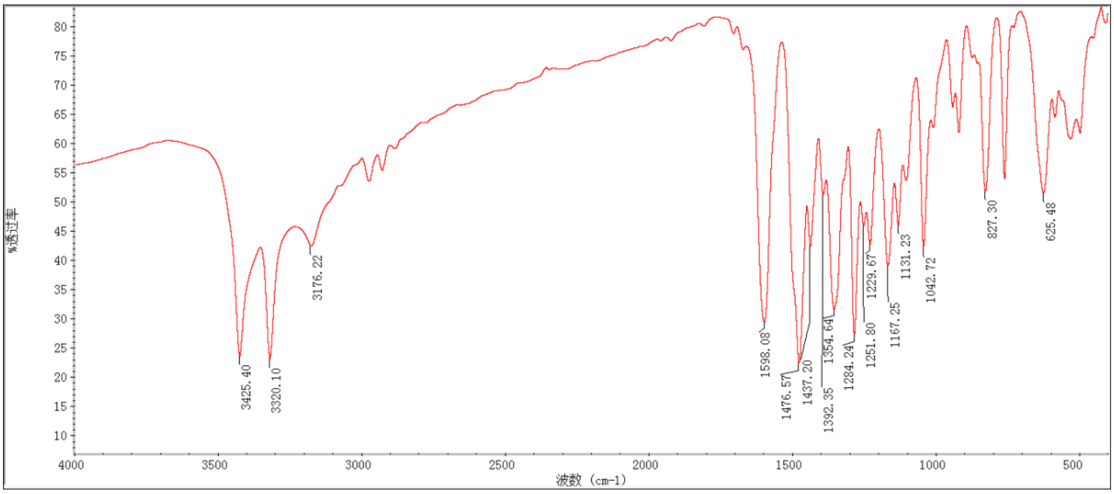
%透过率
波数（cm-1）
3425.40
3320.10
3176.22
1598.08
1476.57
1437.20
1392.35
1354.64
1284.24
1251.80
1229.67
1167.25
1131.23
1042.72
827.30
625.48

10b

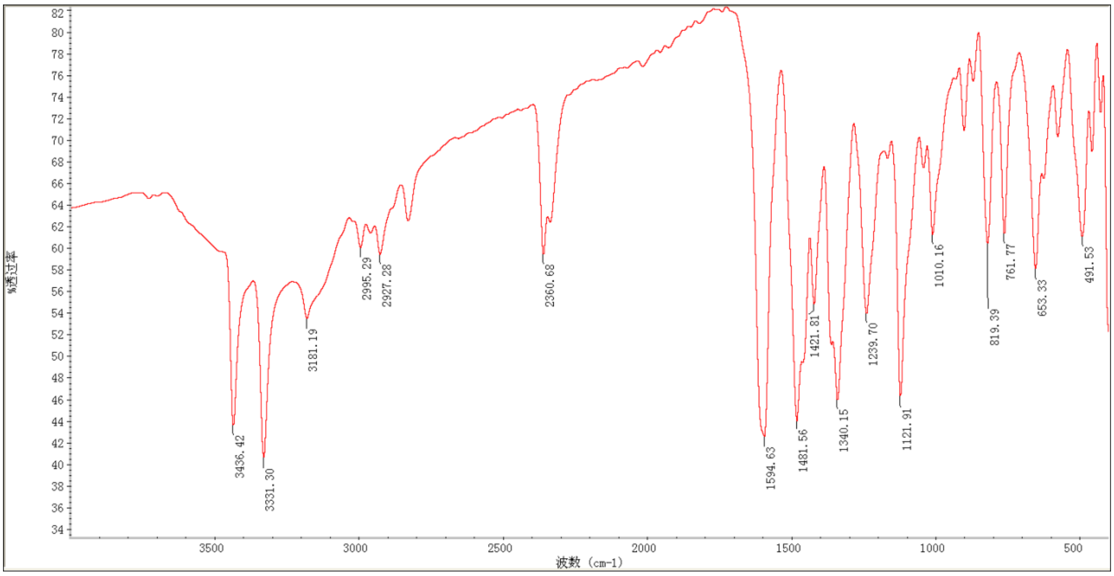
%透过率
波数（cm-1）
3436.42
3331.30
3181.19
2995.29
2927.28
2360.68
1594.63
1481.56
1421.81
1340.15
1239.70
1121.91
1010.16
819.39
761.77
653.33
491.53

11b

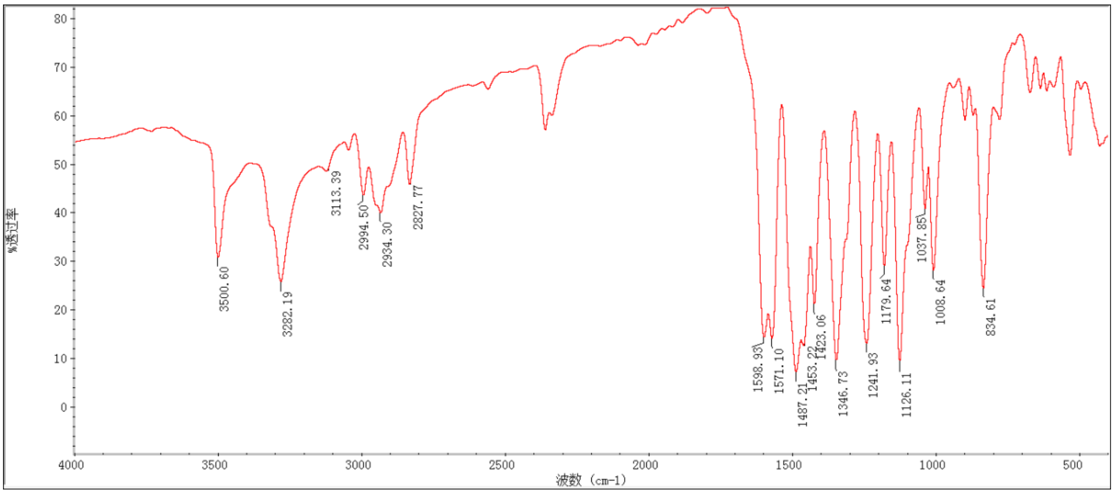
%透过率
波数（cm-1）
3500.60
3282.19
3113.39
2994.50
2934.30
2827.77
1598.93
1571.10
1487.21
1453.22
1423.06
1346.73
1241.93
1179.64
1126.11
1037.85
1008.64
834.61

12b

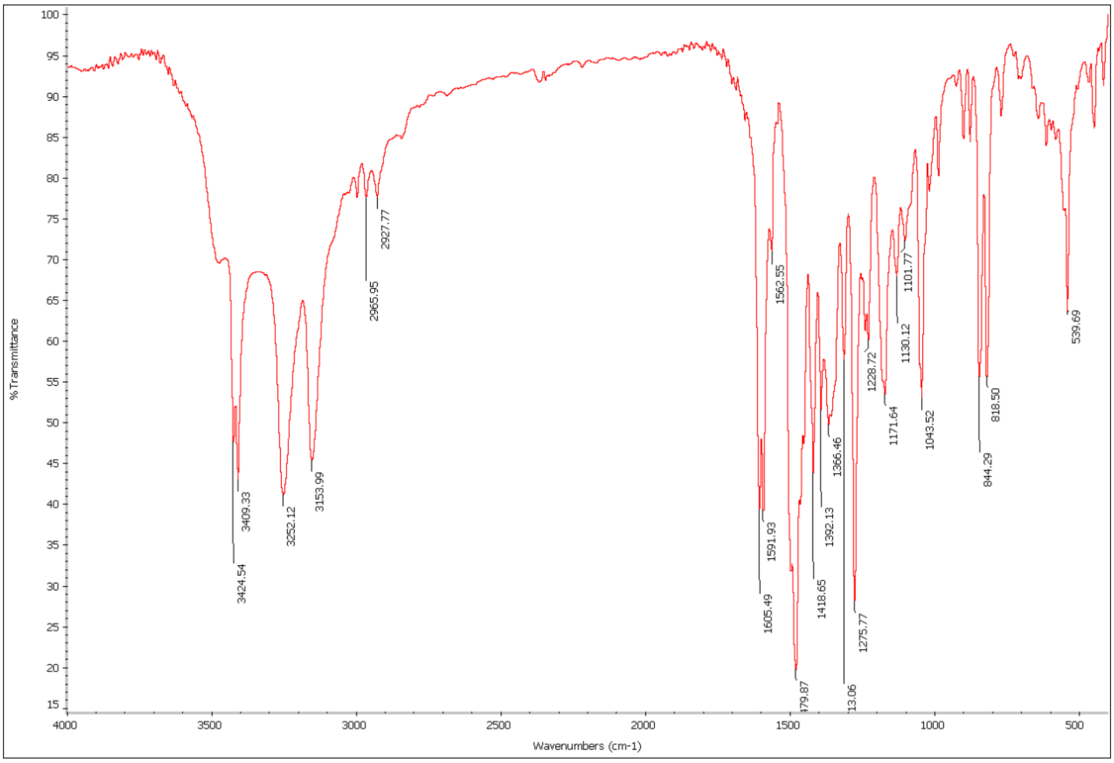

Figure S5. The representative HPLC chromatogram of compound **1b-12b**.

Purity determination of compound **1b**

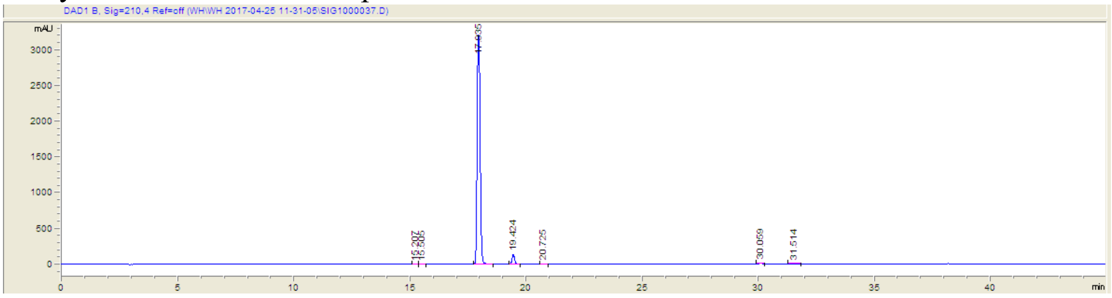

	RT	Area	Height	Peak width	Symmetry factor	%Area
1	15.207	19.4	2.7	0.1123	0.881	0.065
2	15.505	20.1	2.8	0.1119	0.884	0.067
3	17.935	28596.9	3203.1	0.1422	0.852	95.773
4	19.424	1057.7	136.3	0.1205	0.865	3.542
5	20.725	25.3	3.2	0.1201	0.85	0.085
6	30.059	113.4	13.5	0.13	0.928	0.380
7	31.514	26.3	1.9	0.2063	0.56	0.088

Purity determination of compound **2b**

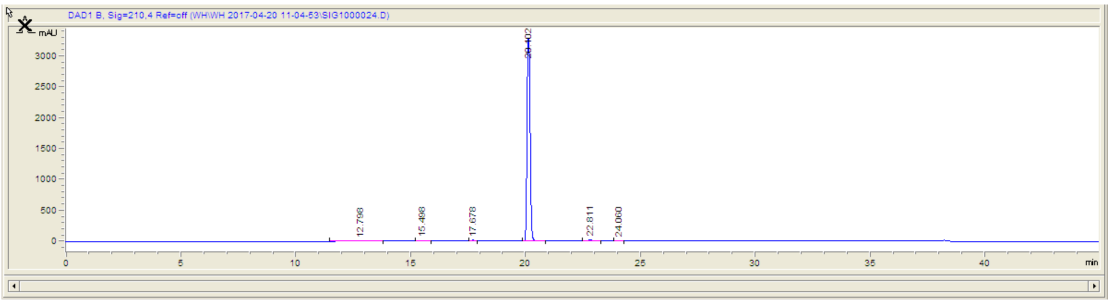

	RT	Area	Height	Peak width	Symmetry factor	%Area
1	12.798	574.2	7.3	1.0407	1.437	1.792
2	15.498	120.6	11.4	0.1514	0.816	0.376
3	17.678	147.3	16.3	0.1336	0.787	0.460
4	20.102	30824.5	3289.7	0.1493	0.875	96.218
5	22.811	261.7	24.4	0.1571	0.793	0.817
6	24.06	107.7	9.5	0.1636	0.957	0.336

Purity determination of compound **3b**

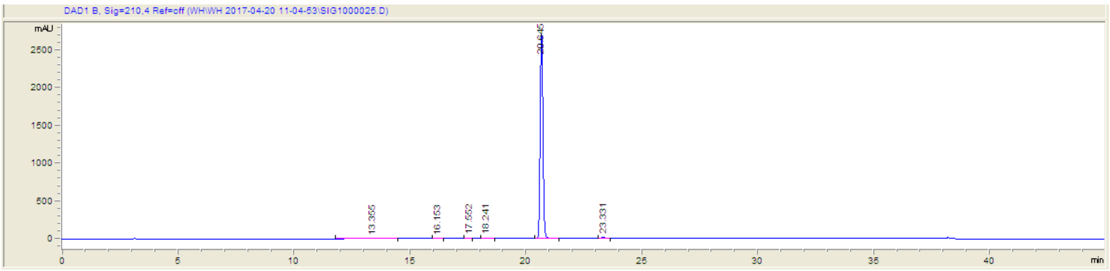

	RT	Area	Height	Peak width	Symmetry factor	%Area
1	13.355	377.9	4.8	1.0586	1.452	1.630
2	16.153	45.5	4.4	0.1515	0.745	0.196
3	17.552	54.3	6.2	0.1349	0.938	0.234
4	18.241	102	10.2	0.1441	0.64	0.44
5	20.645	22429.1	2707	0.1287	0.88	96.768
6	23.331	169.5	18.1	0.1412	0.889	0.731

Purity determination of compound **4b**

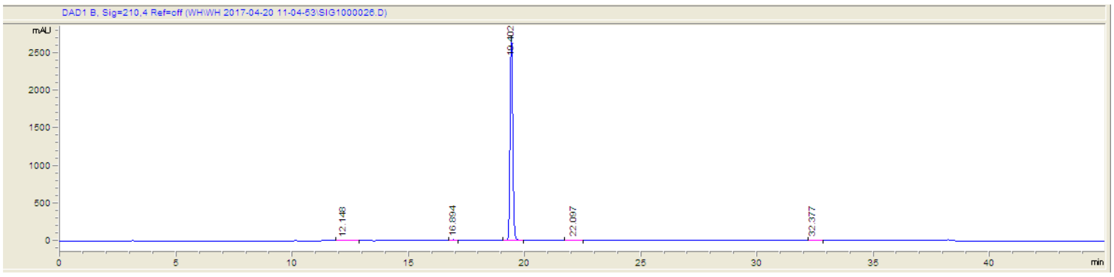

	RT	Area	Height	Peak width	Symmetry factor	%Area
1	12.148	141.6	3.5	0.5001	0.557	0.623
2	16.894	109.5	12.9	0.1267	0.82	0.482
3	19.402	22280.8	2721.3	0.1315	0.866	98.038
4	22.097	111.6	9.6	0.164	0.813	0.419
5	32.377	83.2	6.6	0.1824	0.61	0.366

Purity determination of compound **5b**

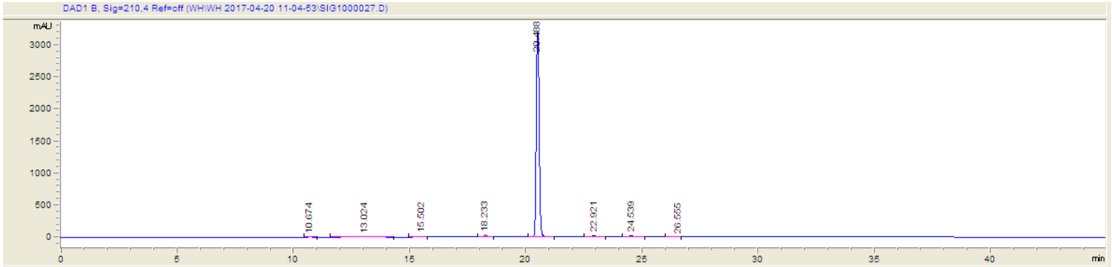

	RT	Area	Height	Peak width	Symmetry factor	%Area
1	10.674	47.9	5.3	0.1367	0.803	0.161
2	13.024	390	5.2	0.9833	1.326	1.313
3	15.502	136.5	15	0.1383	1.125	0.459
4	18.233	260.3	31.6	0.1281	0.785	0.876
5	20.488	28318.7	3207.4	0.143	0.857	95.319
6	22.921	280.2	27.4	0.1512	0.893	0.943
7	24.539	252	23.1	0.1592	0.632	0.848
8	26.555	23.7	8.1E-1	0.3747	3.118	0.080

Purity determination of compound **6b**

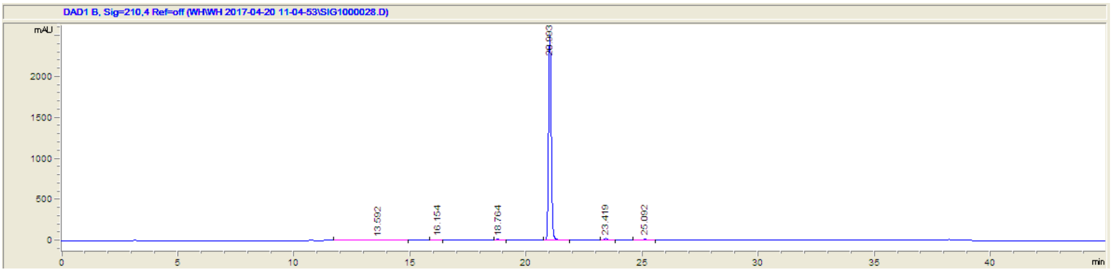

	RT	Area	Height	Peak width	Symmetry factor	%Area
1	13.592	295.8	3.9	0.9823	1.314	1.418
2	16.154	47.8	5.5	0.1337	1.022	0.229
3	18.764	120.7	14.1	0.128	0.708	0.579
4	20.993	20007.5	2498.1	0.1254	0.857	95.940
5	23.419	208.1	21.9	0.1431	0.789	0.998
6	25.092	174.4	14.1	0.1735	0.705	0.836

Purity determination of compound **7b**

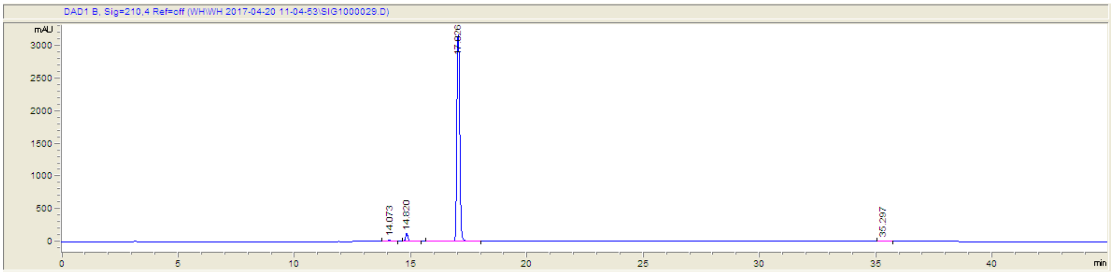

	RT	Area	Height	Peak width	Symmetry factor	%Area
1	14.073	184.7	23.8	0.1187	0.829	0.648
2	14.82	881.8	117.2	0.1158	0.822	3.095
3	17.026	27329.1	3157.9	0.1369	0.823	95.891
4	35.297	104.2	7.9	0.2067	0.937	0.366

Purity determination of compound **8b**

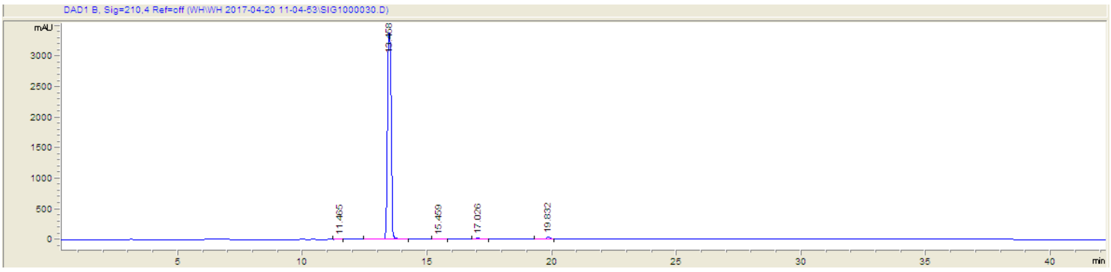

	RT	Area	Height	Peak width	Symmetry factor	%Area
1	11.465	114.1	14.2	0.1219	0.989	0.343
2	13.458	32450.4	3379.2	0.152	0.861	97.543
3	15.459	113.2	9	0.178	0.771	0.340
4	17.026	214.5	24.6	0.1296	0.781	0.645
5	19.832	375.7	43.6	0.1326	0.753	1.129

Purity determination of compound **9b**

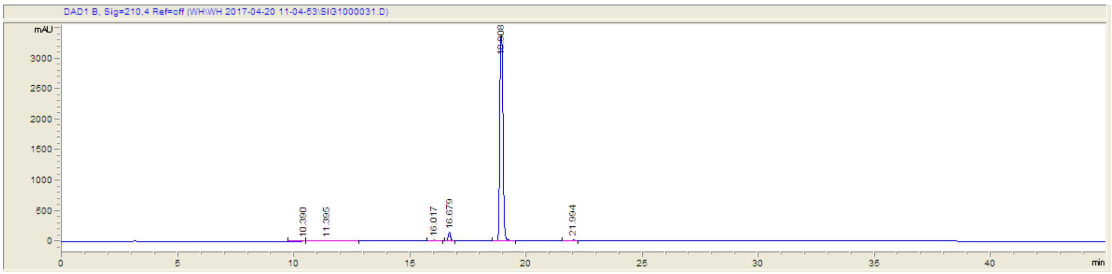

	RT	Area	Height	Peak width	Symmetry factor	%Area
1	10.39	26.8	2.9	0.1369	1.203	0.079
2	11.395	270.7	4.1	0.8545	1.013	0.795
3	16.017	134.3	15.5	0.1329	0.806	0.394
4	16.679	1037.6	137.1	0.1163	0.832	3.046
5	18.908	32395.2	3384.5	0.1517	0.836	95.103
6	21.994	198.8	21.5	0.14	1.017	0.584

Purity determination of compound **10b**

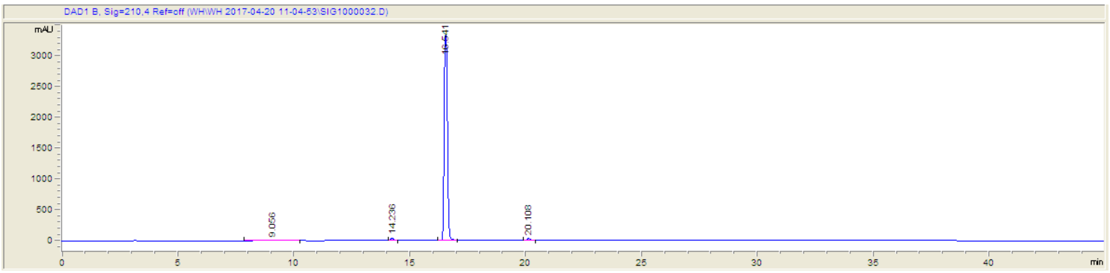

	RT	Area	Height	Peak width	Symmetry factor	%Area
1	9.056	407.1	6.6	0.8444	1.235	1.219
2	14.236	342.8	45.7	0.1155	0.841	1.027
3	16.541	32389.3	3344.9	0.157	0.817	96.980
4	20.108	258.6	29.9	0.1288	0.795	0.774

Purity determination of compound **11b**

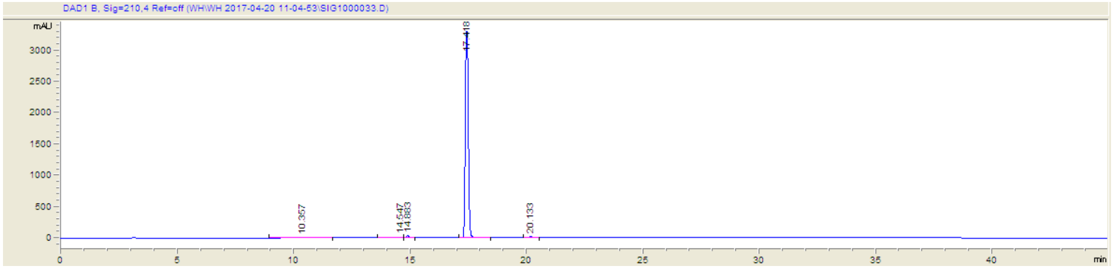

	RT	Area	Height	Peak width	Symmetry factor	%Area

1	10.357	408.7	6.3	0.8807	1.373	1.326
2	14.547	105.4	6.6	0.2139	1.438	0.342
3	14.883	266.6	32.8	0.1227	0.743	0.865
4	17.418	29855.2	3301	0.1455	0.849	96.835
5	20.133	194.9	19.6	0.1443	1.004	0.632

Purity determination of compound **12b**

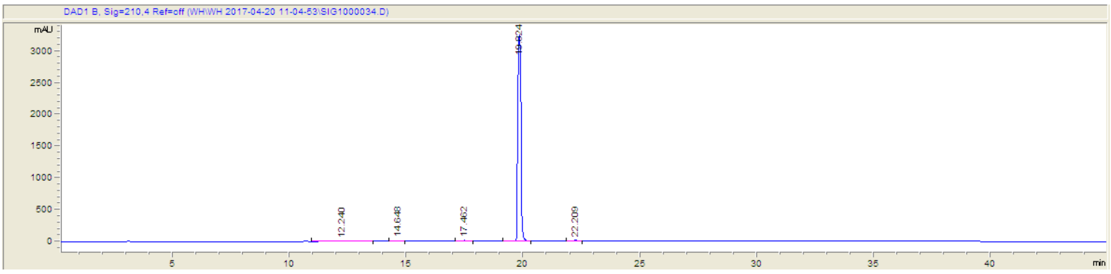

	RT	Area	Height	Peak width	Symmetry factor	%Area
1	12.24	385.1	4.8	1.0289	1.041	1.279
2	14.648	113.8	8.5	0.1886	1.032	0.378
3	17.462	225.8	19.1	0.1698	0.831	0.750
4	19.824	29074.3	3248.3	0.1444	0.855	96.604
5	22.209	297.4	20.1	0.2043	0.789	0.988
